# Modeling the Reaction of Carboxylic Acids and Isonitriles in a Self‐Assembled Capsule

**DOI:** 10.1002/chem.202001735

**Published:** 2020-07-22

**Authors:** Henrik Daver, Julius Rebek, Fahmi Himo

**Affiliations:** ^1^ Department of Organic Chemistry Arrhenius Laboratory Stockholm University 106 91 Stockholm Sweden; ^2^ The Skaggs Institute for Chemical Biology and Department of Chemistry The Scripps Research Institute 10550 North Torrey Pines Road La Jolla California 92037 USA; ^3^ Center for Supramolecular Chemistry and Catalysis Shanghai University Shanghai 200444 P.R. China; ^4^ Present address: Department of Drug Design and Pharmacology University of Copenhagen Universitetsparken 2 2100 Copenhagen Denmark

**Keywords:** density functional calculations, host–guest systems, molecular capsules, reaction mechanisms, self-assembly

## Abstract

Quantum chemical calculations were used to study the reaction of carboxylic acids with isonitriles inside a resorcinarene‐based self‐assembled capsule. Experimentally, it has been shown that the reactions between *p‐*tolylacetic acid and *n*‐butyl isonitrile or isopropyl isonitrile behave differently in the presence of the capsule compared both with each other and also with their solution counterparts. Herein, the reasons for these divergent behaviors are addressed by comparing the detailed energy profiles for the reactions of the two isonitriles inside and outside the capsule. An energy decomposition analysis was conducted to quantify the different factors affecting the reactivity. The calculations reproduce the experimental findings very well. Thus, encapsulation leads to lowering of the energy barrier for the first step of the reaction, the concerted α‐addition and proton transfer, which in solution is rate‐determining, and this explains the rate acceleration observed in the presence of the capsule. The barrier for the final step of the reaction, the 1,3 O→N acyl transfer, is calculated to be higher with the isopropyl substituent inside the capsule compared with *n*‐butyl. With the isopropyl substituent, the transition state and the product of this step are significantly shorter than the preceding intermediate, and this results in energetically unfavorable empty spaces inside the capsule, which cause a higher barrier. With the *n*‐butyl substituent, on the other hand, the carbon chain can untwine and hence uphold an appropriate guest length.

## Graphical abstract

**Filling the capsule**: Reactions of a carboxylic acid with *n*‐butyl and isopropyl isonitriles in a self‐assembled capsule were investigated by DFT. For R=*i*Pr, the transition state and the product of the final, acyl transfer step are significantly shorter than the preceding intermediate, and this results in energetically unfavorable empty spaces inside the capsule, which cause a higher barrier, whereas for R=*n*Bu the C_4_ chain can untwine and thus maintain an appropriate guest length.

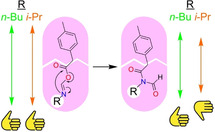

## Introduction

Molecular cages are host compounds with interior cavities in which guests are confined in small spaces and shielded from interactions with the solvent. Such hosts have in many cases been found to act as reaction vessels, in which guests can react or undergo photochemical conversions.^1^, ^2^, ^3^, ^4^, ^5^, ^6^, ^7^, ^8^, ^9^, ^10^, ^11^, ^12^, ^13^, ^14^ A special case is the class of containers called capsules, which are hosts that offer no or limited contact with solvent for their guests. The microscopic environment inside a capsule thus differs from the bulk solvent. In the presence of such hosts, selectivity, rate acceleration, and even catalysis have been observed for a wide range of chemical transformations not readily attained in solution. Examples include cyclizations,^15^, ^16^, ^17^, ^18^ Diels–Alder reactions,^19^, ^20^, ^21^, ^22^ C−X activation,^23^, ^24^ rearrangements,^25^, ^26^, ^27^, ^28^ cycloadditions,^29^, ^30^ olefin oxidation and metathesis,^31^, ^32^ ring opening,^33^, ^34^ condensation,^35^, ^36^ hydrolysis,^37^, ^38^ and hydration.^39^ In short, confinement controls reactivity.

**Figure 1:**
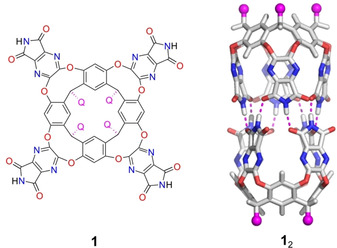
Resorcinarene‐based cavitand **1** and capsule **1**_2_. Q=C_11_H_23_ in the experiments is modeled by a methyl group in the calculations.

One such capsule host system is formed by self‐assembly of two resorcinarene‐based cavitands **1** into capsule **1**_2_ (Figure 1) in the presence of suitably sized guests.^40^ A number of interesting reactivities have been observed inside this host,^28^, ^29^, ^33^, ^39^ one particular case of which is the reaction of carboxylic acids and isonitriles.^27^

**Scheme 1:**
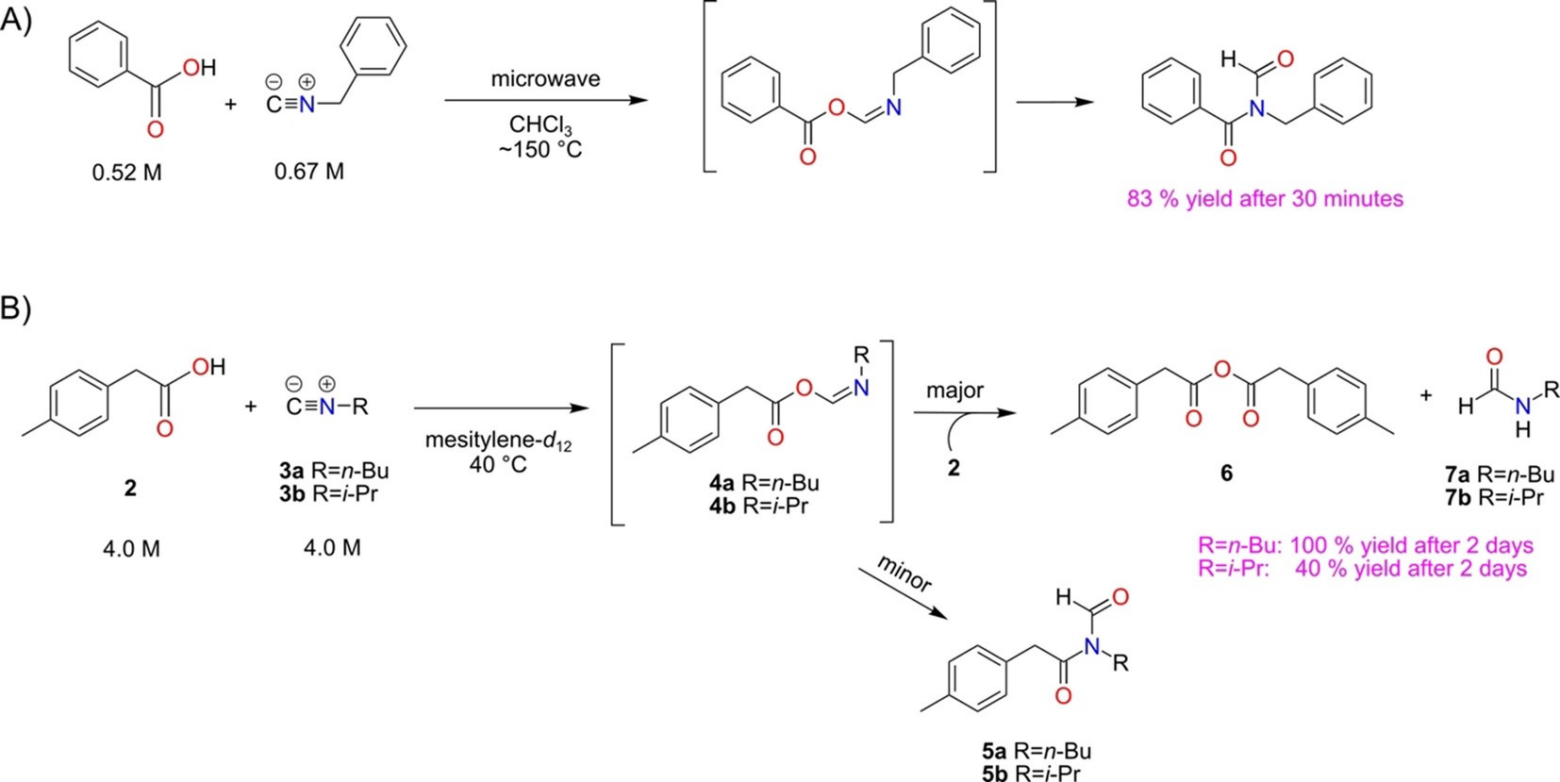
A) Reaction between benzoic acid and benzyl isonitrile yielding *N‐*benzyl‐*N*‐formylbenzamide under microwave irradiation.^41^ B) Reaction between *p‐*tolylacetic acid **2** and isonitriles **3** yields anhydride **6** and formamides **7** as major products at lower temperatures.^27^

The reaction of carboxylic acids and isonitriles was earlier reported by Li and Danishefsky using microwave heating at 150 °C in CHCl_3_, which yielded *N‐*formyl amides (Scheme 1 A).^41^ The mechanism of this reaction was soon after investigated by DFT calculations, which confirmed that the reactants initially form an acyl imidate intermediate, which then undergoes a 1,3 O→N acyl transfer to form the *N‐*formyl amide product.^42^, ^43^

Rebek and co‐workers studied the same reaction in mesitylene, both in the absence and in the presence of capsule **1**_2_.^27^ Without the capsule, and at 40 °C and 4.0 m of reagents, *p‐*tolylacetic acid (**2**) and *n*‐butyl isonitrile (**3 a**) or isopropyl isonitrile (**3 b**) yielded anhydride **6** and formamide **7 a** or **7 b** as major products. This outcome was attributed to the reaction between the proposed acyl imidate intermediate **4** and a second equivalent of carboxylic acid **2** (Scheme 1 B).^27^

**Scheme 2:**
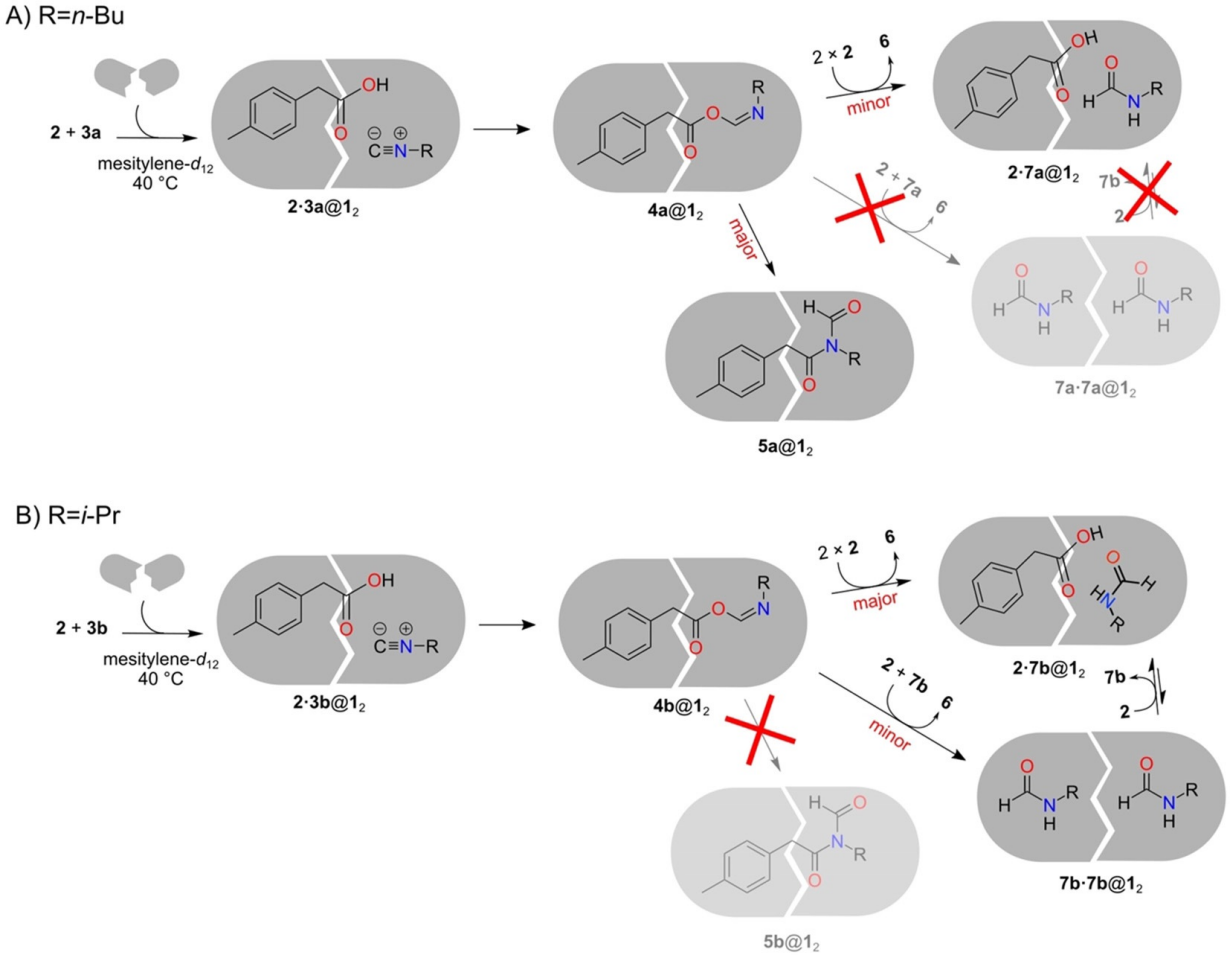
A) Reaction between *p‐*tolylacetic acid (**2**) and *n*‐butyl isonitrile (**3 a**) in the presence of capsule **1**_2_ yields **5 a**@**1**_2_ as the major product. B) Reaction between *p‐*tolylacetic acid (**2**) and isopropyl isonitrile (**3 b**) in the presence of the capsule yields encapsulation complexes with **7 b**, that is, **2⋅7 b**@**1**_2_ and **7 b⋅7 b**@**1**_2_.^27^ Note that **7 b** is depicted as a *cis* amide in **2⋅7 b**@**1**_2_, as this form was found to be more stable inside the capsule in that complex (see Supporting Information for discussion).

Interestingly, in the presence of capsule **1**_2_ and at the same temperature, but with millimolar concentrations of reagents, the reactions between **2** and **3 a** or **3 b** were found to take different courses compared with their solution counterparts and also with each other (Scheme 2).^27^ With **3 a**, *N‐*formyl amide **5 a** becomes the major product and is observed bound inside the capsule (complex **5 a**@**1**_2_). NMR signals of the coencapsulation complex **2⋅7 a**@**1**_2_ were also observed but were smaller in magnitude compared with those of **5 a**@**1**_2_. With **3 b**, on the other hand, the reaction outcome was different. In the absence of **1**_2_, small amounts of **5 b** (1 %) were observed, but in the presence of the capsule no signals corresponding to **5 b**@**1**_2_ could be detected. Instead, only the products of anhydride formation were obtained, in the form of coencapsulation complexes **2⋅7 b**@**1**_2_ and **7 b⋅7 b**@**1**_2_.^27^ In addition, the tentative acyl imidate intermediate **4 b** could be observed transiently for the first time, in the form of host–guest complex **4 b**@**1**_2_.

For both **3 a** and **3 b**, it was proposed that the initial α‐addition reaction occurs inside the capsule, and that the resulting intermediate **4** can leak out to the solution, where it can react with **2** to yield formamide **7**, which then can bind to the capsule.^27^ Importantly, the reactions of both isonitriles were found to be accelerated in the presence of the capsule compared with the solution. At millimolar concentrations, all of the reagents were consumed after 20 h in the presence of **1**_2_, whereas in its absence the reactions went to completion after 2 d or more at molar concentrations.^27^

Herein, the mechanisms of the reactions of **2** with **3 a** and **3 b** were studied inside and outside **1**_2_, in order to evaluate the influence of the capsule on the reactions. In particular, we sought rationalization for two experimental observation: 1) that the capsule accelerates the reactions, and 2) the divergent reactivities of **3 a** and **3 b**, in that product **5** is more favored in presence of the capsule for the case of the longer *n*‐butyl substituent, but less favored with the bulkier isopropyl substituent. To this end, quantum chemical calculations were carried out with dispersion‐corrected DFT. We have previously employed the same techniques to study two different reactions inside the same capsule, namely the cycloaddition between azide and acetylene^44^ and the decomposition of *N‐*nitrosoamides.^45^ A number of other computational studies in recent years have used similar methodologies to investigate various aspects of reactions in confined spaces.^46^, ^47^, ^48^, ^49^, ^50^, ^51^, ^52^, ^53^, ^54^, ^55^, ^56^, ^57^, ^58^

Capsule **1**_2_ has no endohedral functionalization, and thus provides a mainly nonpolar and relatively static void for molecules to bind and react in. In general, several ideas have been put forward to account for the rate acceleration achieved by such complexes. These include 1) favorable binding of transition states over reactants,^15^, ^16^, ^44^, ^46^ 2) binding of substrates in a favorable (preorganized) conformation for reaction,^15^, ^23^, ^30^ 3) reduction of the entropic penalty,^22^, ^26^, ^44^, ^51^ 4) increased concentration of reacting species inside the capsule compared to outside,^19^, ^29^, ^59^ 5) reactive conformations that are longer‐lived inside the capsule than in solution,^59^ 6) electrostatic stabilization of transition states,^3^, ^35^, ^60^ and 7) elimination of solvent reorganization during the reaction.^59^ By using various decomposition schemes, it is possible to partition the energies obtained by the calculations to evaluate the validity of these scenarios, and also to examine sources of various selectivities, as has been done for a number of reactions in confined spaces recently.^21^, ^22^, ^30^, ^31^, ^44^, ^45^, ^48^, ^58^, ^61^

## Computational Methods

All DFT calculations were performed with the B3LYP‐D3(BJ) functional,^62^, ^63^, ^64^, ^65^, ^66^, ^67^, ^68^ as implemented in the Gaussian 09 software.^69^ Full geometry optimizations were carried out for all studied species with the 6‐31G(d,p) basis set. Conformational searches were performed by manually setting up and evaluating at least ten conformers per complex, in order to make sure that the lowest‐energy geometry was obtained. On the basis of the most stable structures, single‐point energy calculations were performed with the 6‐311+G(2d,2p) basis set. The energies were further corrected with the three‐body term of the DFT‐D3 method,^66^ which is not included in the Gaussian 09 implementation. Solvation effects in mesitylene at 40 °C were calculated with the COSMO‐RS model,^70^ as implemented in the COSMOtherm software,^71^ at the BP86/TZVP level of theory.^62^, ^72^, ^73^ To account for the change in reference state from gas phase to solution, a correction of *RT* ln(24.5 L mol^−1^×1 mol L^−1^)=+2.0 kcal mol^−1^ was added for all species. At the same level of theory as the geometry optimizations, vibrational eigenmodes and entropic corrections were calculated at 313.15 K and 1 atm, according to the quasi‐rigid rotor‐harmonic oscillator (qRRHO) model.^74^ In this approach the inherent overestimation of entropic contributions from low‐frequency vibrational modes in the standard RRHO method is corrected.

## Results and Discussion

In the present study, the reactions between tolylacetic acid **2** and *n*‐butyl isonitrile **3 a** or isopropyl isonitrile **3 b** were investigated both in the presence and in the absence of capsule **1**_2_. Comparisons between the two cases are made, and similarities and differences between the reactions with the two isonitriles are highlighted.

### Reaction outside the capsule

**Figure 2:**
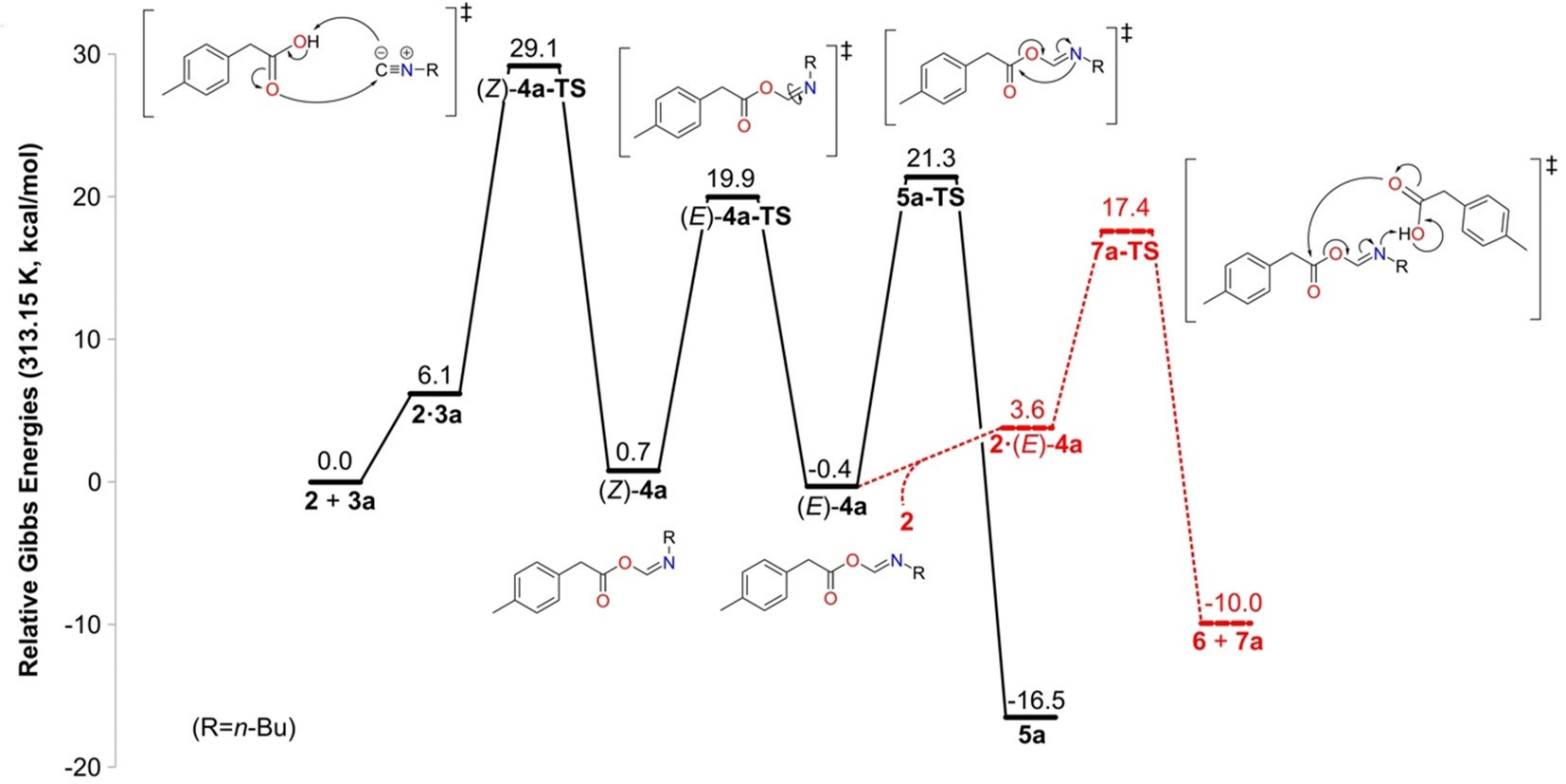
Calculated energy profile for the reaction between carboxylic acid **2** and isonitrile **3 a** in the absence of capsule.

To assess the influence of the capsule on the mechanism and the energetics of the reaction, a detailed understanding of the reaction in absence of the capsule is necessary first. As shown in Scheme 1 B, reaction of **2** with **3** can lead to either the major products anhydride **6** and formamide **7**, or to the minor product *N‐*formyl amide **5**. The latter reaction has been considered computationally previously.^42^, ^43^ Here, we present in detail the results concerning the reaction between **2** and **3 a**, and the reaction with **3 b** will be briefly compared at the end of the section. The obtained Gibbs energy profile for all steps of the reaction between **2** and **3 a** is given in Figure 2, and the optimized geometries of the intermediates and transition states (TSs) are given in the Supporting Information.

The two reactants assemble first into complex **2⋅3 a**, in which a hydrogen bond is formed between the carboxylic acid and the terminal carbon atom of **3 a**. Interestingly, there is also a weak interaction between the butyl and tolyl substituents of the reactants (see the Supporting Information). Formation of this complex is calculated to be endergonic by 6.1 kcal mol^−1^. Next, a proton transfer takes place from **2** to the carbon center of **3 a** concertedly with α addition of **2** to **3 a**. The TS for this step, denoted (*Z*)‐**4 a‐TS**, is calculated to be 29.1 kcal mol^−1^ higher in energy than the separate reactants (Figure 2), and the resulting intermediate is the *Z* isomer of **4 a**, (*Z*)‐**4 a**, which is calculated to lie at +0.7 kcal mol^−1^ relative to the reactants.

For the rearrangement step to occur, an isomerization around the C=N bond must first take place.^43^ In the TS for this step, denoted (*E*)‐**4 a‐TS**, the C=N−C bond angle is close to linear, and the energy is calculated to be 19.2 kcal mol^−1^ relative to (*Z*)‐**4 a** (Figure 2). The resulting (*E*)‐**4 a** is slightly more stable than the *Z* isomer, by 1.1 kcal mol^−1^.

From (*E*)‐**4 a**, the 1,3 O→N acyl transfer reaction takes place to yield the *N‐*formyl amide product **5 a**. In the corresponding TS, **5 a‐TS**, the nitrogen atom attacks the carbonyl carbon atom, and the C−O bond is cleaved concertedly. The barrier is calculated to be 21.3 kcal mol^−1^ relative to the separate reactants, that is, 21.7 kcal mol^−1^ relative to (*E*)‐**4 a**. This step is very exergonic, and product **5 a** is 16.5 kcal mol^−1^ lower than the reactants (Figure 2). For the acyl transfer to take place, rotation around the O−C single bond in (*E*)‐**4 a** must take place to bring the carbonyl carbon atom closer to the nitrogen atom. This rotation was found to occur as a part of the transition state **5 a‐TS** and not as a separate step.

The results so far are quite similar to the previous computational studies on related substrates.^42^, ^43^ The major difference is that in the current calculations both entropy and dispersion corrections are included in the final energies, whereas in the previous studies only solution‐phase enthalpies were reported. Thus, the separate reactants (**2**+**3 a**) are now calculated to be 6.1 kcal mol^−1^ lower in energy than the reactant supercomplex **2⋅3 a**, and this leads to a higher overall barrier for the first step of the reaction compared with the previous studies. The obtained rate‐determining barrier of 29.1 kcal mol^−1^ (Figure 2) agrees well with the experimental observation that the reaction occurs on the order of days.^27^

Interestingly, in the experiments with **2** and **3 a**, no rearrangement product **5 a** was observed. Instead, anhydride **6** and formamide **7 a** were obtained at 40 °C.^27^ As discussed in the Introduction, this was attributed to the reaction between the imidate intermediate **4 a** and **2** (Scheme 1 B). This part of the reaction has, to the best of our knowledge, not been studied computationally before. A conformational analysis revealed that the reaction between **4 a** and **2** can only occur from the *E* isomer of **4 a**. The complex between these two compounds, **2⋅**(*E*)‐**4 a**, is calculated to be 4.0 kcal mol^−1^ higher in energy than the separate molecules. In **2⋅**(*E*)‐**4 a**, the carboxyl group of **2** forms a hydrogen bond with the nitrogen atom of (*E*)‐**4 a**, and the O−C single bond of the latter is now rotated in anticipation of the following step (see structure in Supporting Information). From the complex, the carbonyl oxygen atom of **2** performs a nucleophilic attack at the carbonyl carbon atom of (*E*)‐**4 a**, and concertedly a proton transfer takes place from **2** to the nitrogen atom of (*E*)‐**4 a**, and the ester C−O bond in (*E*)‐**4 a** is cleaved (Figure 2). The TS for this reaction, denoted **7 a‐TS**, is calculated to be 17.8 kcal mol^−1^ higher in energy than **2**+(*E*)‐**4 a**, and the resulting products **6** and **7 a** are 9.6 kcal mol^−1^ lower. This barrier is thus 3.9 kcal mol^−1^ lower than that of **5 a‐TS**, which is consistent with the experimental observation of **6** and **7 a** being the products, and not **5 a**.^27^

In the reaction of **2** with isonitrile **3 b**, both the geometries and the energies were found to be very similar to those obtained with **3 a** (see Supporting Information). The formation of formamide **7 b** is also here favored compared with *N‐*formyl amide **5 b**. The energy difference between **7 b‐TS** and **5 b‐TS** was calculated to be 4.2 kcal mol^−1^, which is in agreement with the experimental observation of a **7 b**:**5 b** ratio of 99:1.^27^

In summary, the details of the reaction mechanisms in the absence of the capsule were elucidated and the calculations are fully in agreement with the experimental results in that the formamide products **7 a**/**7 b** are formed with lower barriers compared to the rearrangement products **5 a**/**5 b**.

### Reaction of tolylacetic acid and *n*‐butyl isonitrile inside the capsule

**Figure 3:**
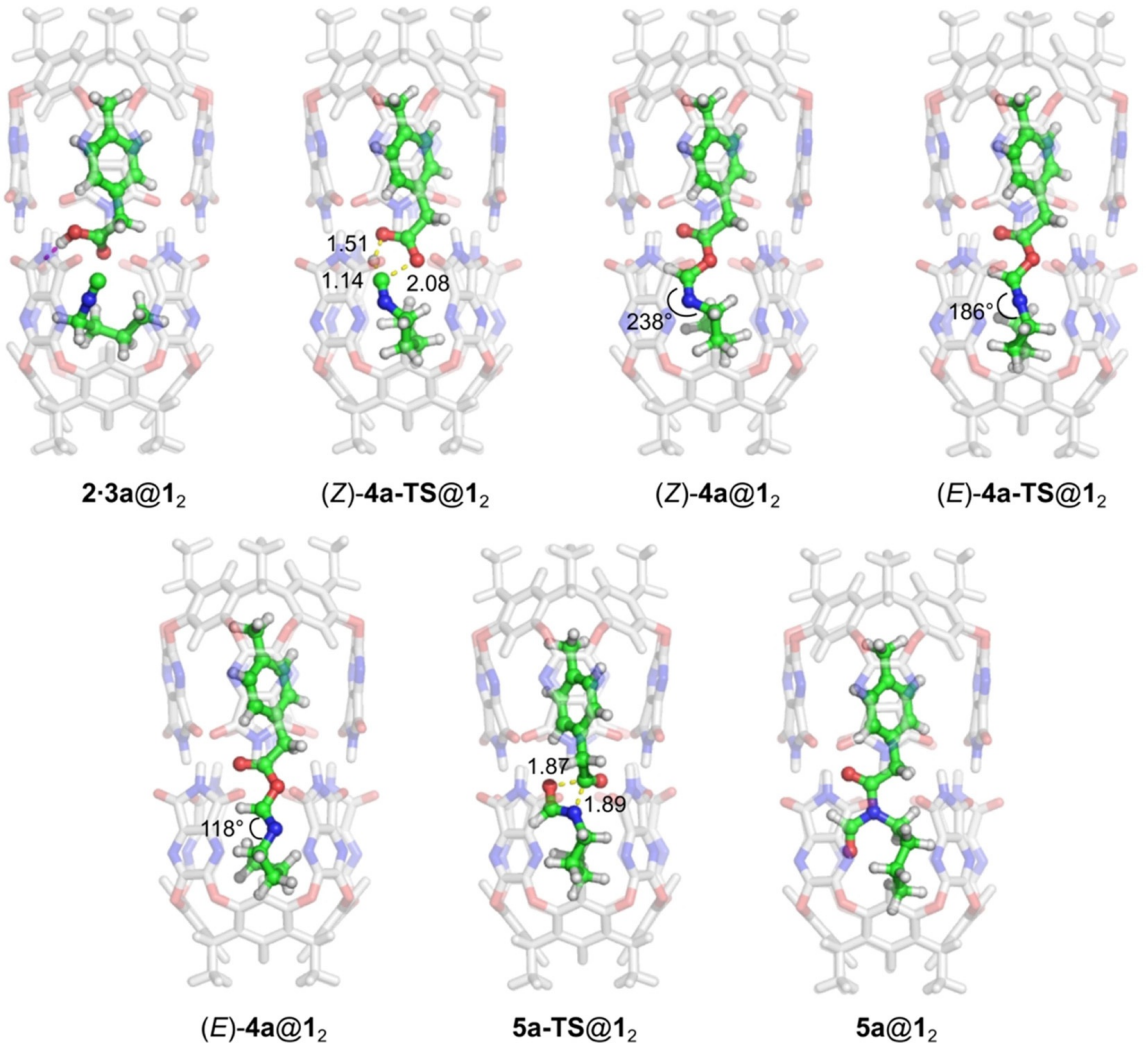
Optimized geometries of intermediates and transition states in the reaction between **2** and **3 a** inside capsule **1_2_**.

In the experimental study, the encapsulated reactant complex **2⋅3 a**@**1**_2_ could be observed initially.^27^ Computationally, the most stable geometry of such a complex shows the two guests occupying one half of the capsule each (Figure 3). Consequently, the weak interaction between the butyl and tolyl moieties of the reactants found in the **2⋅3 a** complex outside the capsule (see above) is not present inside the capsule. Interestingly, the carboxylic acid forms a hydrogen bond to a carbonyl group of the capsule rim, as shown in Figure 3. However, several other binding complexes could be located that are close in energy, for example, one in which a hydrogen bond is formed between the two encapsulated molecules. This structure is calculated to be only 0.2 kcal mol^−1^ higher in energy, and thus from the calculations, the guests can be expected to have some conformational freedom in this reactant complex. In all of the low‐energy complexes, the substrates bind inside the capsule such that the polar groups are oriented toward the middle of the capsule and the nonpolar groups toward the ends. The reacting fragments are thus close to each other, poised for reaction. To fit into the capsule, the *n*‐butyl substituent of **3 a** must be contorted away from a linear conformation, such that the four carbon atoms bend upward in a *gauche* conformation (Figure 3) instead of the *trans* conformation that is more favorable in solution.

Next, the α‐addition step inside the capsule takes place via transition state (*Z*)‐**4 a‐TS**@**1**_2_, which is geometrically very similar to (*Z*)‐**4 a‐TS** outside the capsule. The step is calculated to have a barrier of 20.2 kcal mol^−1^ relative to **2⋅3 a**@**1**_2_, which is 2.8 kcal mol^−1^ lower than the energy difference between **2⋅3 a** and (*Z*)‐**4 a‐TS** in the absence of the capsule (23.0 kcal mol^−1^, Figure 2). The result of the α‐addition step is complex (*Z*)‐**4 a**@**1**_2_ (Figure 3), which is 6.9 kcal mol^−1^ lower in energy than **2⋅3 a**@**1**_2_.

**Figure 4:**
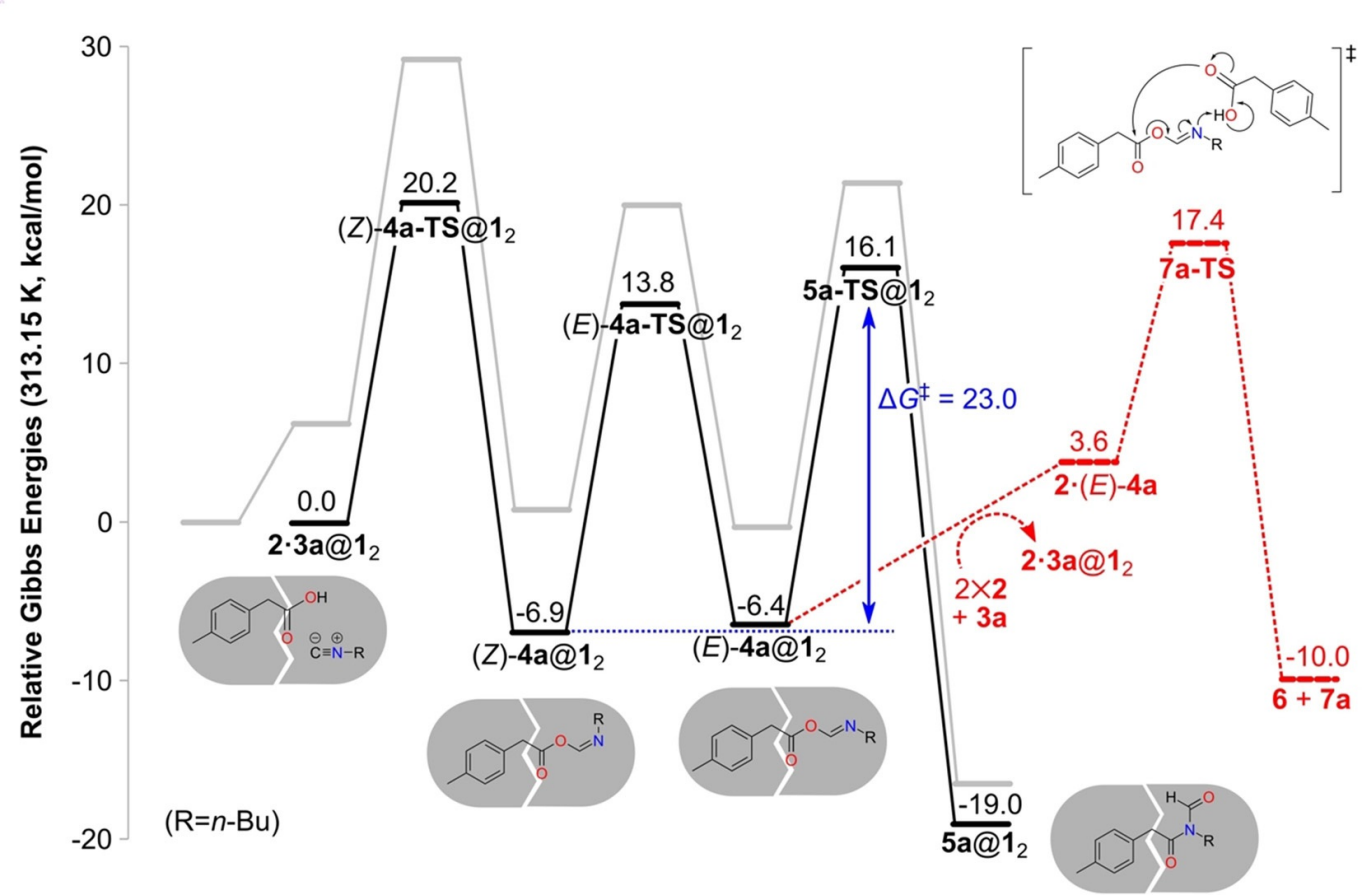
Calculated energy profile for the reaction between **2** and **3 a** inside capsule **1_2_** (black line). The energy profile for the reaction outside the capsule is given for comparison (gray line). The energies of the release of intermediate (*E*)‐**4 a** and its subsequent reaction with **2** to form **6** and **7 a** are also shown (red line).

From (*Z*)‐**4 a**@**1**_2_, the isomerization step takes place via (*E*)‐**4 a‐TS**@**1**_2_, with a barrier of 20.7 kcal mol^−1^, somewhat higher than in the absence of the capsule (19.2 kcal mol^−1^). The geometry of the guest in the resulting (*E*)‐**4 a**@**1**_2_ resembles very much its geometry outside the capsule. Intermediate (*E*)‐**4 a**@**1**_2_ is calculated to be 0.5 kcal mol^−1^ higher in energy than (*Z*)‐**4 a**@**1**_2_ (as opposed to a difference of −1.1 kcal mol^−1^ in the absence of capsule). From (*E*)‐**4 a**@**1**_2_, the nitrogen atom then attacks the carbonyl carbon atom in **5 a‐TS**@**1**_2_, with concomitant dissociation of the ester C−O bond (Figure 3). The barrier for this step is calculated to be 23.0 kcal mol^−1^ with respect to (*Z*)‐**4 a**@**1**_2_, and the resulting complex **5 a**@**1**_2_ is 12.1 kcal mol^−1^ more stable. The encapsulated product complex **5 a**@**1**_2_ is thus 19.0 kcal mol^−1^ more stable than the reactant complex **2⋅3 a**@**1**_2_ (Figure 4). As in the case of the reaction outside the capsule, rotation around the O−C single bond in (*E*)‐**4 a** is necessary for the 1,3 O→N acyl transfer reaction to take place, and this facile rotation can be considered as a part of the following transition state, **5 a‐TS**@**1**_2_.

In the alternative reaction pathway, (*E*)‐**4 a** can be released to solution to react with another molecule of **2**, forming anhydride **6** and formamide **7 a**. As indicated in Scheme 2 A, minor amounts of the complex **2⋅7 a**@**1**_2_ were detected experimentally (the optimized structure of this complex is given in the Supporting Information).^27^ As shown in Figure 4, this reaction is calculated to have a barrier of 23.8 kcal mol^−1^, that is, 1.3 kcal mol^−1^ higher than that of the acyl transfer reaction inside the capsule, **5 a‐TS**@**1**_2_. This result is in good agreement with the experimental observation that, in the presence of the capsule, **5 a**@**1**_2_ is the major product and **2⋅7 a**@**1**_2_ is a minor product (Scheme 2 A).^27^

Despite this good agreement, we note here that the barrier for the formation of **7 a** involves the guest‐exchange step [(*E*)‐**4 a**@**1**_2_+2 **2**+**3 a**→**2⋅3 a**@**1**_2_+**2⋅**(*E*)‐**4 a**], and the calculated Gibbs energies of this kind of step can be associated with larger uncertainties compared to other steps when using the quantum‐chemical methodology. A related issue is concerned with the barrier for guest release, which has not been considered here. In previous experimental studies with other guests, release rates have been reported that correspond to barriers of approximately 20 kcal mol^−1^.^75^ This effect could also add to the uncertainty in the calculation of the competition between the two pathways.

The overall energy profiles for the reactions inside and outside the capsule are compared in Figure 4. Interestingly, although the step sequence is the same, the rate‐limiting step inside the capsule is different from that outside. Outside the capsule, the initial α‐addition step was found to be rate‐limiting, while inside the capsule, the barrier for this step is lower than that for the acyl transfer step (20.2 vs. 23.0 kcal mol^−1^). Notably, the overall barrier inside the capsule is significantly lower than that outside, 23.0 versus 29.1 kcal mol^−1^, in excellent agreement with the rate acceleration observed experimentally.^27^

As shown in Figure 4, the barrier for the initial α addition is lowered the most in the presence of the capsule compared to the solution reaction, by approximately 9 kcal mol^−1^. To elucidate the origins of this barrier reduction, an energy decomposition analysis was conducted, by following the same procedure as in our previous studies on other reactions in the same capsule (details are given in the Supporting Information).^44^, ^45^ This analysis showed that the entropic contribution to the barrier is reduced by 4.3 kcal mol^−1^ due to encapsulation. A further reduction of the barrier, amounting to 4.7 kcal mol^−1^, is calculated to stem from the capsule undergoing more favorable interactions with the TS than with the reactants.

To summarize this section, encapsulation changes the energy profile of the reaction between **2** and **3 a** significantly compared with solution. The barrier for the α‐addition step, calculated to be rate‐determining in the absence of the capsule, is lowered by as much as 9 kcal mol^−1^. Instead, the 1,3 O→N acyl transfer step becomes rate‐determining inside the capsule, with a barrier of 23 kcal mol^−1^. The barrier for formation of product **5 a** inside the capsule is calculated to be lower than the barrier for the combined release of the imidate intermediate **4 a** and its reaction with **2** outside the capsule to yield formamide **7 a**.

### Reaction of tolylacetic acid and isopropyl isonitrile inside the capsule

**Figure 5:**
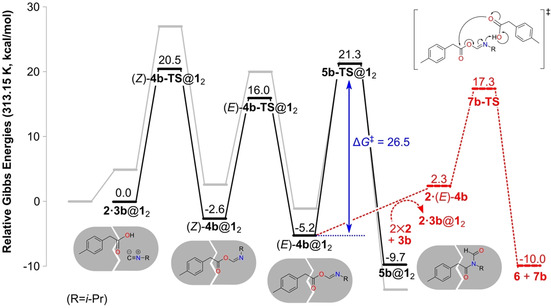
Calculated energy profile for the reaction between **2** and **3 b** inside capsule **1_2_** (black line). The energy profile for the reaction outside the capsule is shown as a gray line for comparison. The energies of the release of intermediate (*E*)‐**4 b** and its subsequent reaction with **2** to form **6** and **7 b** are shown in red.

**Figure 6:**
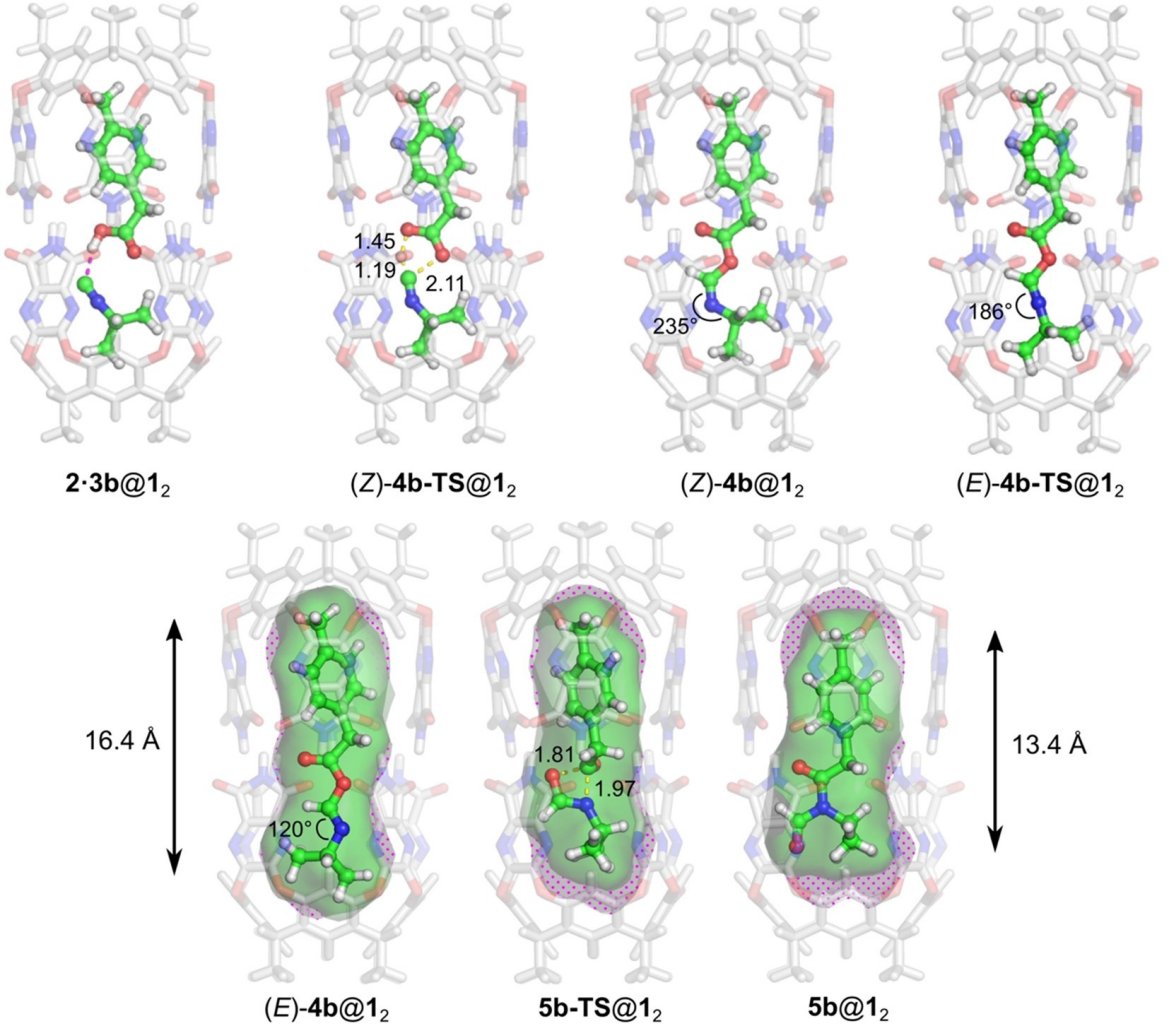
Optimized geometries of intermediates and transition states in the reaction between **2** and **3 b** inside capsule **1_2_**. For the last three species, the inner void of the capsule is shown in polka‐dotted gray and the molecular surface of the guest is shown in green. The guest length is indicated for complexes (*E*)‐**4 b**@**1**_2_ and **5 b**@**1**_2_. Capsule voids and guest surfaces were calculated with PyMOL.^77^ Guest lengths were calculated along the capsule axis by employing Bondi atomic radii.^78^

We now turn to the reaction of **2** with isopropyl isonitrile (**3 b**). Whereas the presence of the capsule was experimentally found to accelerate the conversion of *n*‐butyl isonitrile **3 a** to **5 a**, it was observed to prevent the same reaction for **3 b**. The fact that the intermediate **4 b** could be detected transiently indicates that the barrier for 1,3 O→N acyl transfer is somehow raised by encapsulation.^27^ We performed similar calculations for substrate **3 b** as for **3 a** above. The obtained energy profile is given in Figure 5, and the optimized geometries of the intermediates and transition states along the reaction path are shown in Figure 6.

The optimized geometries with **3 b** are in general quite similar to those with **3 a** shown in Figure 3. However, the calculated energies show significant differences associated with the change of substituent, as can be seen in Figure 5. Although the barrier for the initial α‐addition step is very similar, the resulting (*Z*)‐**4 b**@**1**_2_ intermediate is less stable than its counterpart (*Z*)‐**4 a**@**1**_2_ (−2.6 vs. −6.9 kcal mol^−1^). The other isomer, (*E*)‐**4 b**@**1**_2_, is now more stable than (*Z*)‐**4 b**@**1**_2_, and the barrier for the following acyl transfer step increases to 26.5 kcal mol^−1^ (vs. 23.0 kcal mol^−1^ for **3 a**). The energies of both **5 b‐TS**@**1**_2_ and the product complex **5 b**@**1**_2_ are considerably higher than those of their counterparts in the reaction of **3 a**. The higher barrier of the acyl transfer explains the experimental detection of complex (*Z*)‐**4 b**@**1**_2_.^27^ It also renders the competing pathway more viable, that is, the release of **4 b** and the subsequent reaction with **2** outside the capsule to form anhydride **6** and formamide **7 b**. The barrier for this process is calculated to be 22.5 kcal mol^−1^, which is 4.0 kcal mol^−1^ lower than **5 b‐TS**@**1**_2_ (Figure 5). The formation of **6** and **7 b** is thus favored in the presence of the capsule, which is consistent with the lack of observation of **5 b**@**1**_2_ in the experiments.^27^

Thus, also for isonitrile **3 b**, the calculations reproduce the experimentally observed rate acceleration in the presence of the capsule. The overall barrier, calculated to be 22.5 kcal mol^−1^ [(*E*)‐**4 b**@**1**_2_ to **7 b‐TS**], is significantly lower than the overall barrier outside, which corresponds to the α‐addition step and is calculated to be approximately 27 kcal mol^−1^ (see Supporting Information). Here, we have to make the same reservations as above concerning the uncertainty in the calculations related to the ligand exchange energies and to neglecting the barrier for guest release.

An energy decomposition analysis was undertaken to quantify the reasons for the calculated barrier increase of the acyl transfer step with the isopropyl substituent as compared with *n*‐butyl (see Supporting Information for details). The main difference was found to lie in the interaction energies between the host and the guests. Whereas (*E*)‐**4 a**, **5 a‐TS**, and **5 a** all interact with the capsule with very similar energies, **5 b‐TS** and **5 b** are calculated to be significantly worse guests than their preceding intermediate (*E*)‐**4 b**. The host–guest interaction energies in **5 b‐TS**@**1**_2_ and **5 b**@**1**_2_ are 3.9 and 7.5 kcal mol^−1^ higher than in (*E*)‐**4 b**@**1**_2_, respectively.

Examination of the optimized geometries of **5 b‐TS**@**1**_2_ and **5 b**@**1**_2_ shows that the guests in these two complexes are significantly shorter than those of both (*Z*)‐**4 b**@**1**_2_ and (*E*)‐**4 b**@**1**_2_, by 2–3 Å (see Figure 6). The length (measured along the capsule axis) changes from 16.4 Å for (*E*)‐**4 b** to 14.5 Å for **5 b‐TS** and 13.4 Å for **5 b**. The difference in interaction energy thus correlates inversely with guest length.

The shorter guests in **5 b‐TS**@**1**_2_ and **5 b**@**1**_2_ give rise to empty regions inside the capsule (shown as polka‐dotted areas in Figure 6) that are unfavorable from energetic point of view and result in the higher barrier for the acyl transfer step. With the *n*‐butyl substituent, on the other hand, the guest lengths differ by less than 1 Å on going from (*E*)‐**4 a**@**1**_2_ to **5 a**@**1**_2_. In these structures, the C_4_ chain is contorted from its optimal linear conformation to better fit inside the capsule. In particular, in the acyl transfer step, the chain untwines somewhat to better fit the available space.

Finally, the increased barrier can also be analyzed in terms of the packing coefficient (PC), that is, the fraction of the host cavity that is occupied by guests.^76^ On going from (*E*)‐**4 b**@**1**_2_ to **5 b**@**1**_2_, the PC decreases from 0.59 to 0.53 (see Supporting Information). The lower part of the capsule, as oriented in Figure 6, expands to encompass *N‐*formyl amide **5 b**, whereas the 1,3 O→N acyl transfer does not affect the guest size. A similar trend can be seen in the reaction of the *n*‐butyl isonitrile, but the capsule expansion, and thus the decrease in PC, is smaller in this case (see Supporting Information for details).

## Conclusion

The reactions of carboxylic acid **2** with isonitrile **3 a** or **3 b** have been investigated in the presence of nanocapsule **1**_2_ by using a dispersion‐corrected DFT protocol. The influence of the capsule on the reactions was evaluated by comparing the geometries and obtained energy profiles to the corresponding solution counterparts.

The calculations reproduce the experiments and provide rationalizations for the observations that the presence of the capsule accelerates the reactions and leads to divergent behaviors of substrates **3 a** and **3 b**. With *n*‐butyl isonitrile (**3 a**), the barrier for 1,3 O→N acyl transfer inside the capsule is calculated to be lower than the barrier for the combined release of the acyl imidate intermediate (*E*)‐**4 a** and its subsequent reaction with another molecule of carboxylic acid **2** outside the capsule. This results in the formation of product **5 a** inside the capsule, as observed experimentally. With isopropyl isonitrile (**3 b**), on the other hand, a higher barrier is calculated for the acyl transfer step inside the capsule, and it is therefore energetically more favorable to release the (*E*)‐**4 b** intermediate to solution, where it can react with carboxylic acid **2** to form products **6** and **7 b**, in agreement with the experimental findings.

It is argued that the reason for the disfavoring of the acyl transfer in the case of the isopropyl isonitrile substrate is that it leads to significantly shorter transition state and product structures that do not fill the capsule as well as in the case of *n*‐butyl isonitrile. The shorter structures lead to energetically unfavorable voids at the top and bottom ends of the capsule.

## Conflict of interest

The authors declare no conflict of interest.

## Supporting information

As a service to our authors and readers, this journal provides supporting information supplied by the authors. Such materials are peer reviewed and may be re‐organized for online delivery, but are not copy‐edited or typeset. Technical support issues arising from supporting information (other than missing files) should be addressed to the authors.

Supplementary
