# Supplementary material for: Modeling the Reaction of Carboxylic Acids and Isonitriles in a Self‐Assembled Capsule
Source: Chemistry. 2020 Jul 22;26(47):10861–70. doi: 10.1002/chem.202001735 (PMC7522688; doi:10.1002/chem.202001735)
Supplement: Supplementary file 1 — Supplementary [file CHEM-26-10861-s001.pdf]

Chemistry–A European Journal

Supporting Information

Modeling the Reaction of Carboxylic Acids and Isonitriles in a Self-Assembled Capsule

Henrik Daver,[a, d] Julius Rebek, Jr.,[b, c] and Fahmi Himo*[a]

Table of contents

1. Reactions in the absence of capsule S2
2. Geometries of capsule-formamide complexes S5
3. Energy decomposition analysis S6
4. Host and guest volumes S9
5. Absolute energies and energy corrections S10
6. References S12
7. Cartesian coordinates S13

1. Reactions in the absence of capsule

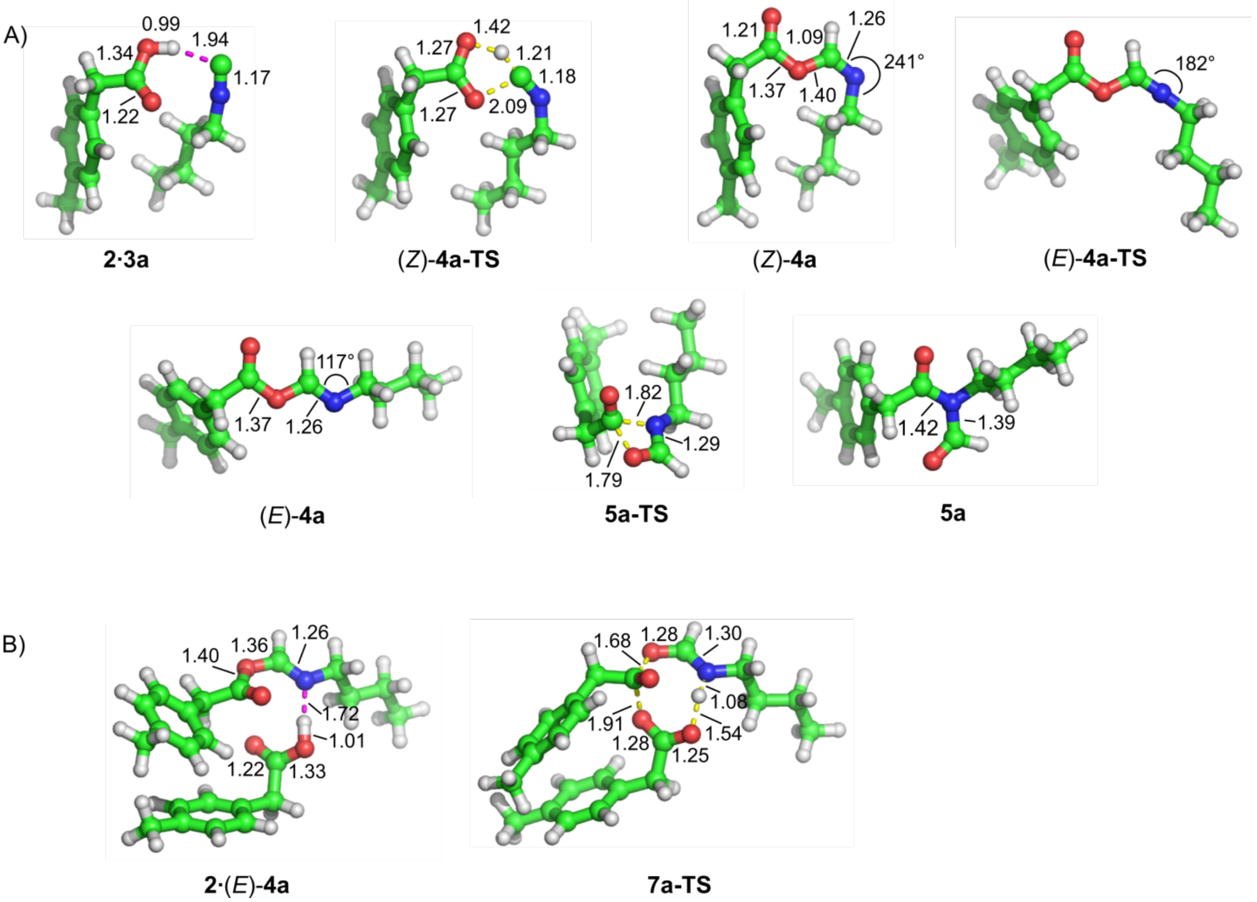

Figure S1. *Optimized geometries of intermediates and transition states for (A) the formation of N-formyl amide* ***5a*** *from carboxylic acid* ***2*** *and isonitrile* ***3a****, and (B) the formation of anhydride* ***6*** *and formamide 7****a*** *from* ***2*** *and (E)-****4a****. Selected distances (Å) and angles are shown.*

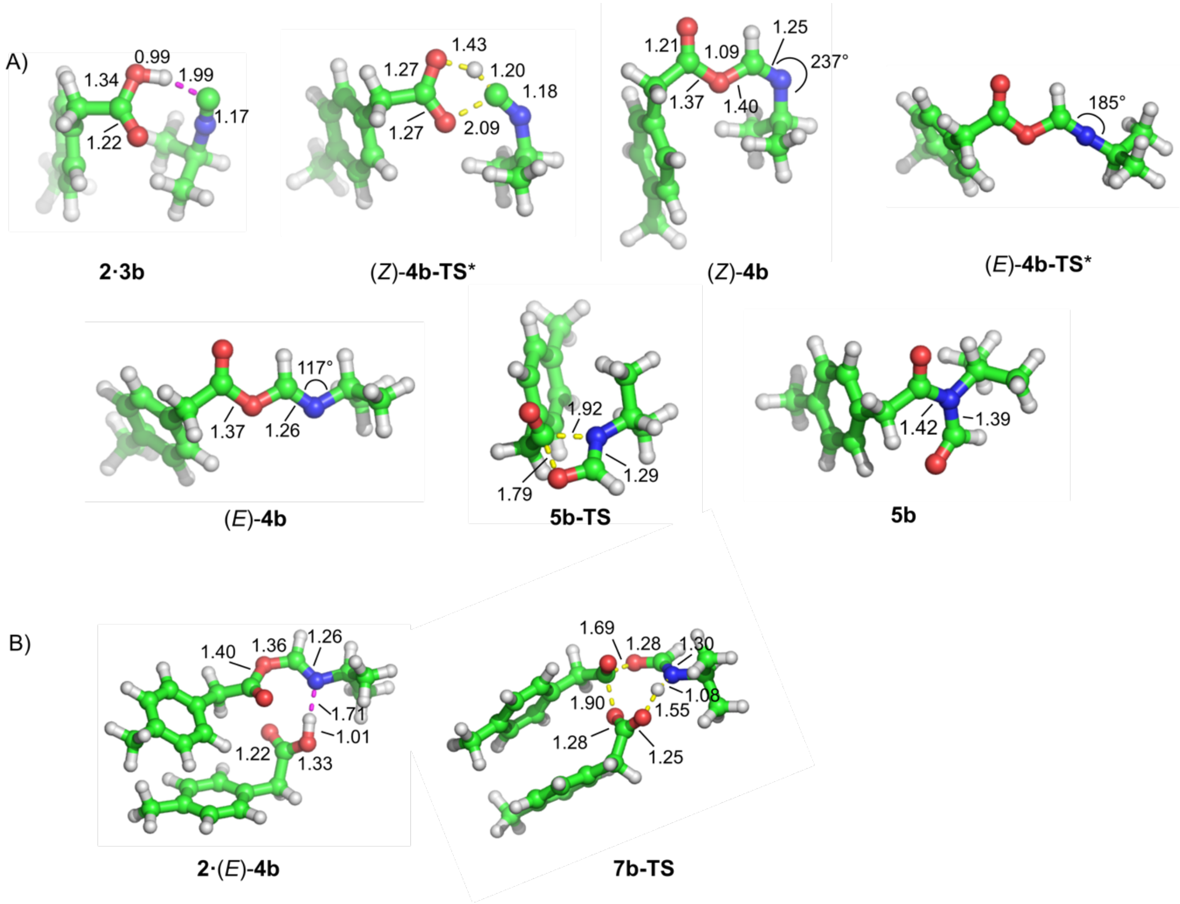

Figure S2. *Optimized geometries of intermediates and transition states for (A) the formation of N-formyl amide **5b** from carboxylic acid **2** and isonitrile **3b** and (B) the formation of anhydride **6** and formamide **7b** from **2** and (*E*)-**4b**.*

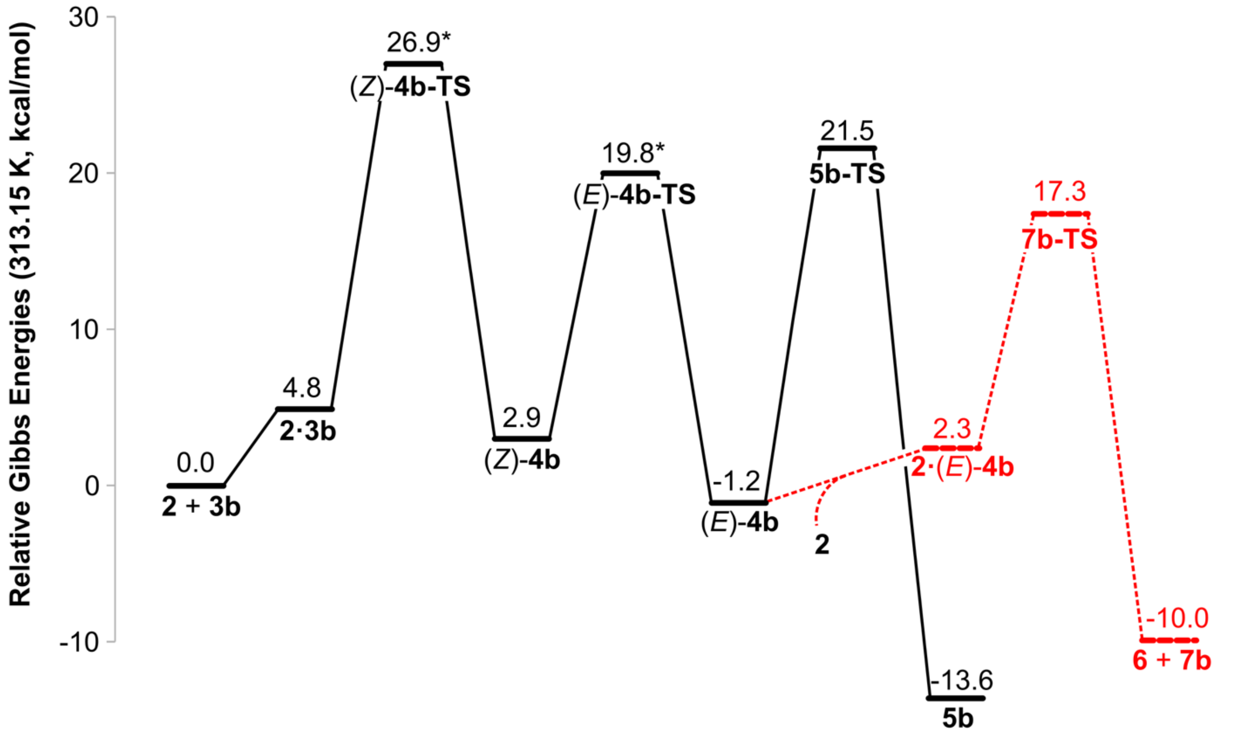

Figure S3. *Calculated energy profile for the reaction between carboxylic acid* ***2*** *and isonitrile* ***3b*** *in the absence of capsule.*

Transition states **7a-TS** and **7b-TS** result in the *cis* conformation of the **7a**/**7b** formamides. Interconversion to the respective *trans* conformers is calculated to have barriers of ca. 22 kcal/mol. The *trans* conformers are calculated to be ca. 1 kcal/mol more stable than the *cis* ones in solution.

Note that (*Z*)-**4b-TS** and (*E*)-**4b-TS** are marked with asterisks (*) in Figures S2 and S3. These geometries have two imaginary frequencies. The additional small (<10*i* cm^{-1}) imaginary frequencies were found to correspond to diffuse vibrations involving the tolyl and propyl moieties. Other conformations of these TSs were found to be several kcal/mol higher. Many attempts were made to eliminate these surplus imaginary frequencies without success. They were therefore replaced by real frequencies of the same magnitudes in the quasi-RRHO calculations. Experience of similar cases shows that the error bar of this treatment is <1 kcal/mol.

2. Geometries of capsule-formamide complexes

The optimized geometries of the lowest energy complexes of **2·7a@1**$_2$, **7a·7a@1**$_2$, **2·7b@1**$_2$ and **7b·7b@1**$_2$ are shown in Figure S4. Interestingly, in the complexes with one *p*-tolylacetic acid and one formamide molecule, i.e. **2·7a@1**$_2$ and **2·7b@1**$_2$, the formamide conformation and the interactions between the acid and the formamide differ depending on the formamide substituent. In **2·7a@1**$_2$, a hydrogen bond is present between the carboxylic acid and the carbonyl oxygen of *cis*-**7a**. In the most stable conformation of **2·7b@1**$_2$, however, *trans*-**7b** is encapsulated and an addition guest-guest hydrogen bond is possible, from the amide of **7b** to the carbonyl oxygen of **2**. There are also interesting differences between the complexes with two formamide guests. In **7a·7a@1**$_2$ a hydrogen bond exists between the formamides, while the two formamides do not interact in **7b·7b@1**$_2$.

Energetically, **2·7a@1**$_2$ and **2·7b@1**$_2$ are similarly stable relative to their respective reactant complexes (-7.1 and -5.5 kcal/mol). However, an interesting observation is that **7b·7b@1**$_2$, experimentally observed in a 1:3 ratio to **2·7b@1**$_2$,[S1] is calculated to be more than 13 kcal/mol higher than **2·7b@1**$_2$. Even though two formamides cannot fill the capsule as well as the combination of one carboxylic acid and one formamide, this energy difference is overestimated by the computational methods. Comparing **2·7a@1**$_2$ with **7a·7a@1**$_2$, the latter is calculated to be only +4.0 kcal/mol higher.

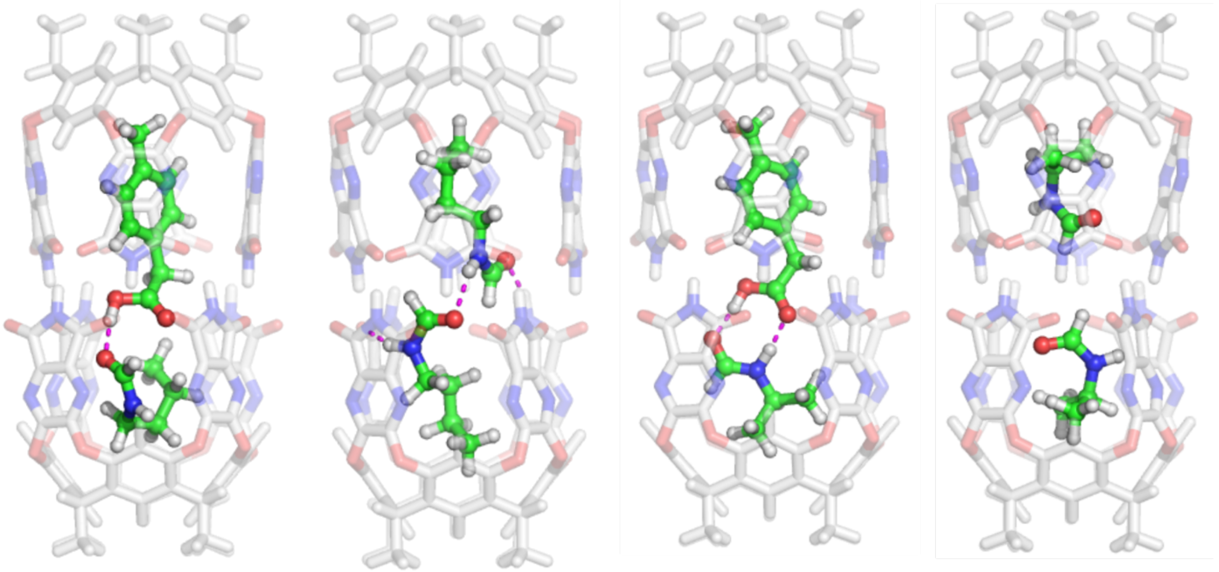

2·7a@1$_2$ -7.1 kcal/mol rel. to **2·3a@1**$_2$

7a·7a@1$_2$ -3.1 kcal/mol rel. to **2·3a@1**$_2$

2·7b@1$_2$ -5.5 kcal/mol rel. to **2·3b@1**$_2$

7b·7b@1$_2$ +7.9 kcal/mol rel. to **2·3b@1**$_2$

Figure S4. *Optimized geometries and relative Gibbs energies of encapsulation complexes with* ***7a*** *or* ***7b****.*

3. Energy decomposition analysis

To quantify the different factors affecting the reactivity inside the capsule, an energy decomposition analysis was undertaken following the same procedure as in our previous papers.[S2,S3]

First the α-addition step was evaluated. In Figure S5, the analysis is done for three cases: i) *n*-butyl substituent with reactant structure **2·3a@1**$_2$ (Figure S5A), ii) *n*-butyl substituent with reactant structure (**2·3a@1**$_2$)' (Figure S5B), and iii) isopropyl substituent with reactant structure **2·3b@1**$_2$ (Figure S5C). The two reactant complexes **2·3a@1**$_2$ and (**2·3a@1**$_2$)' differ in that in **2·3a@1**$_2$, a hydrogen bond is formed between **2** and a carbonyl oxygen at the capsule seam, while in (**2·3a@1**$_2$)', **2** forms a hydrogen bond to the isonitrile moiety of **3a**. The two structures differ only by 0.2 kcal/mol.

The energetic effect of the capsule can be roughly divided into enthalpic and entropic contributions. For all cases, it is found that the two parts have similar contributions to the lowering of the barrier: i) $\Delta\Delta H^{\ddagger}$ = -4.6 kcal/mol, $-T\Delta\Delta S^{\ddagger}$ = -4.3 kcal/mol; ii) $\Delta\Delta H^{\ddagger}$ = -3.7 kcal/mol, $-T\Delta\Delta S^{\ddagger}$ = -5.4 kcal/mol; and iii) $\Delta\Delta H^{\ddagger}$ = -2.5 kcal/mol, $-T\Delta\Delta S^{\ddagger}$ = -3.9 kcal/mol (Figure S5). This is quite similar to the cycloaddition reaction in the same capsule studied previously, for which strain and interaction effects were calculated to lower the barrier by 3.1 kcal/mol and entropic effects were found to further reduce it by 3.3 kcal/mol.[S2]

Further decomposition of the enthalpic contribution into contributions from host and guest distortions and host-guest interaction shows that this analysis is quite sensitive to whether **2·3a@1**$_2$ or (**2·3a@1**$_2$)' is chosen as the reactant complex. With the **2·3a@1**$_2$ (case i), the host is significantly distorted as one of the hydrogen bonds between the two cavitands is broken. In the decomposition analysis, this shows up as host distortion lowering the barrier significantly (-7.4 kcal/mol) since the host is significantly less distorted in the TS structure. This is compensated by the host-guest interaction energy being greater in the reactant structure than in the TS, giving a host-guest interaction term of +7.5 kcal/mol. When cases ii and iii are compared, however, the enthalpic terms are much more similar.

The fact that the entropic and enthalpic contributions to the barrier lowering of the α-addition step are of similar magnitudes can be rationalized as follows. Enthalpically, the capsule interacts better with the TS than with the reactants. This is in part due to the breaking of the vdW interactions that in solution exist between the aryl group of **2** and the *n*-butyl or isopropyl moieties in **3a** and **3b**, respectively (see Figures S1 and S2), thus destabilizing the reactant complex compared to the solution case. The entropic effects contribute to the barrier in solution by 4.4 kcal/mol, while the barrier inside the capsule is hardly affected (Figure S5). In solution, the ground state of the reaction is the separated reactants, but in presence of capsule the reactant supercomplex becomes the lowest-energy species. The capsule eliminates thus the entropic penalty of bringing the reactants together.

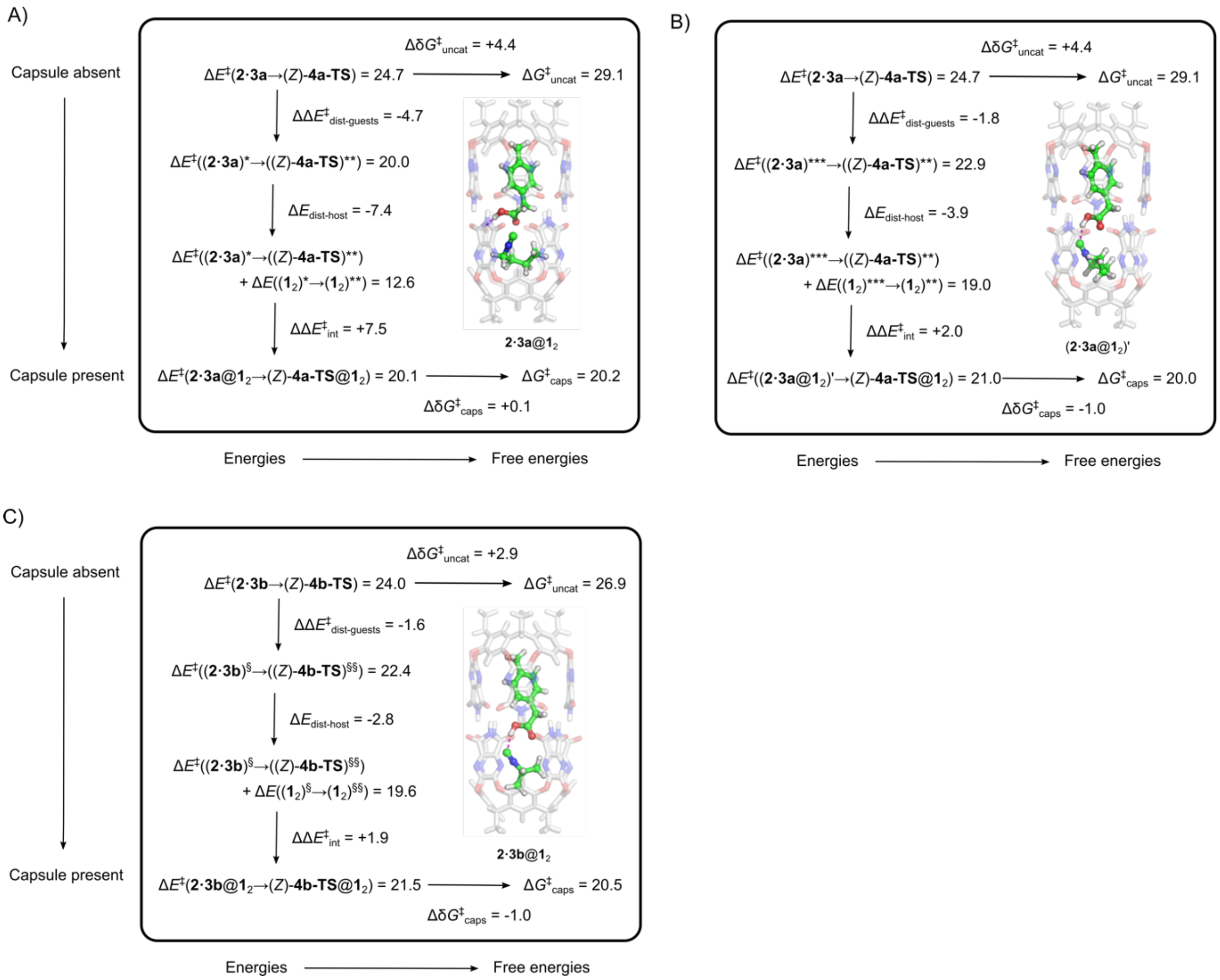

Figure S5. *Energy decomposition analyses of how capsule* ***1_2*** *lowers the barrier for α-addition, where the reactant complex is either (A)* ***2·3a@1_2****, (B) (****2·3a@1_2****)' or (C)* ***2·3b@1_2****. All energies are given in kcal/mol.*

** = Guest or host structure from* ***2·3a@1_2****.*

*** = Guest or host structure from (Z)-****4a-TS@1_2****.*

**** = Guest or host structure from (****2·3a@1_2****)'.*

§ = Guest or host structure from ***2·3b@1_2****.*

*§§ = Guest or host structure from (Z)-****4b-TS@1_2****.*

An energy decomposition analysis of how the capsule affects the barrier for the acyl transfer step with both substituents is presented in Figure S6. Overall, the capsule is calculated to raise both barriers, but to different extents: +0.8 kcal/mol with the *n*-butyl substituent and +3.8 kcal/mol with isopropyl. This is found to stem from the difference in host-guest interaction energy, which is calculated to be negligible in the case of the *n*-butyl substituent (-0.2 kcal/mol, Figure S6A) and quite significant is the case of the isopropyl substituent (+4.0 kcal/mol, Figure S6B).

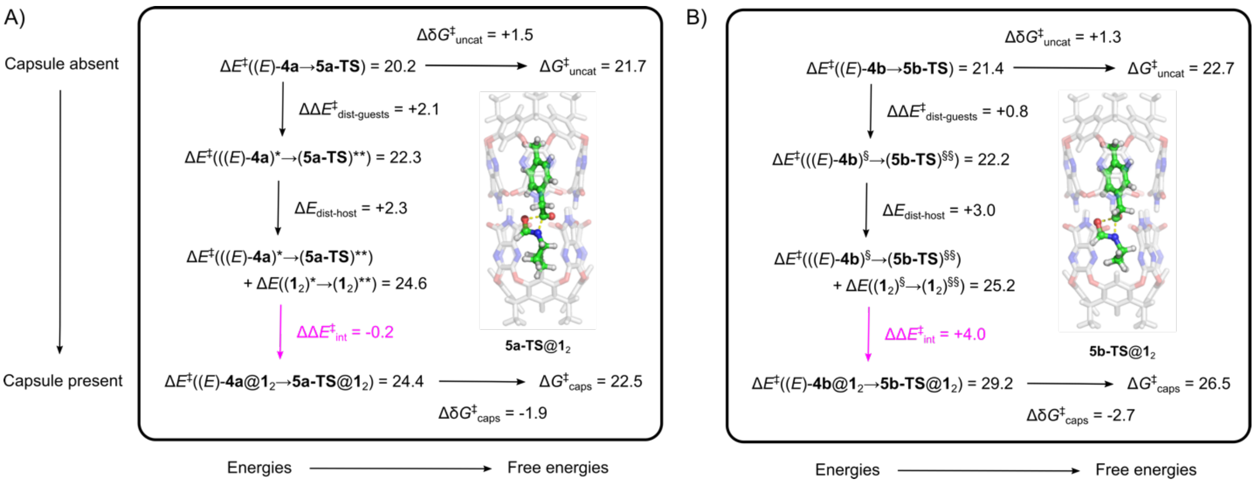

Figure S6. *Energy decomposition analyses of how capsule* $\mathbf{1}_2$ *affects the barrier for the 1,3 O→N acyl transfer step, starting from (A) (E)-**4a@1**$_2$ or (B) (E)-**4b@1**$_2$. All energies are given in kcal/mol.*

** = Guest or host structure from (E)-**4a@1**$_2$.*

*** = Guest or host structure from **5a-TS@1**$_2$.*

*§ = Guest or host structure from (E)-**4b@1**$_2$.*

*§§ = Guest or host structure from **5b-TS@1**$_2$.*

4. Host and guest volumes

The calculated van der Waals volumes of the guests (calculated with VOIDOO)[S4] and cavity volumes of the host (calculated with the sphere-packing method as in Ref. S2) are given in Table S1 for all studied host-guest complexes.

The packing coefficients (PCs),[S5] also listed, are quite similar throughout the reaction, with the exception of the 1,3 O→N acyl transfer step with the isopropyl substituent: from (*E*)-**4b**@$\mathbf{1}_2$ to **5b**@$\mathbf{1}_2$, the PC decreases from 0.59 to 0.53. This is due to an increase in the cavity volume of the host, which mainly stems from the lower part of the capsule (as shown in Figure 6 in the paper) widening to accommodate the *N*-formyl amide product **5b**. A related effect is observed in going from (*E*)-**4a**@$\mathbf{1}_2$ to **5a**@$\mathbf{1}_2$, but the decrease in PC is smaller there (from 0.60 to 0.57). This is because the lower part of the capsule (in Figure 3) is already widened in order to accommodate the longer *n*-butyl group in (*E*)-**4a**@$\mathbf{1}_2$, and thus less distortion is needed in order to make room for product **5a**.

For comparison, the cavity volumes were also calculated with BetaVoid.[S6] While volumes estimated with this method are much smaller, the same trend as above is observed: the cavity volume increases from (*E*)-**4**@$\mathbf{1}_2$ to **5**@$\mathbf{1}_2$ (Table S1).

Table S1. *Calculated guest van der Waal volumes and host cavity volumes, in Å^3.*

	Guest vdW volume	*Cavity volume (sphere-packing)*	*Packing coefficient*	*Cavity volume (BetaVoid)*
**R=*n*-Bu**				
**2·3a**@$\mathbf{1}_2$	241	427	0.57	163
(*Z*)-**4a-TS**@$\mathbf{1}_2$	236	397	0.59	142
(*Z*)-**4a**@$\mathbf{1}_2$	234	402	0.58	187
(*E*)-**4a-TS**@$\mathbf{1}_2$	234	393	0.60	140
(*E*)-**4a**@$\mathbf{1}_2$	234	387	0.60	134
**5a-TS**@$\mathbf{1}_2$	235	417	0.56	154
**5a**@$\mathbf{1}_2$	231	407	0.57	149
**R=*i*-Pr**				
**2·3b**@$\mathbf{1}_2$	223	386	0.58	132
(*Z*)-**4b-TS**@$\mathbf{1}_2$	219	385	0.57	130
(*Z*)-**4b**@$\mathbf{1}_2$	218	391	0.56	136
(*E*)-**4b-TS**@$\mathbf{1}_2$	217	375	0.58	128
(*E*)-**4b**@$\mathbf{1}_2$	217	369	0.59	123
**5b-TS**@$\mathbf{1}_2$	218	415	0.53	153
**5b**@$\mathbf{1}_2$	217	410	0.53	149

5. Absolute energies and energy corrections

The calculated energies of all compounds in the article are given in Table S2.

Table S2. *Calculated absolute energies and energy corrections (in atomic units).*

	Absolute energy B3LYP-D3(BJ)/ 6-311+G(2d,2p)[a]	***Solvation Gibbs energy[b] (COSMOtherm)***	***Thermal correction to Gibbs energy (quasi-RRHO, 298.15 K)***
**2**	-499.650716	-0.008943	0.132689
**3a**	-250.762496	-0.005502	0.098376
**2·3a**	-750.428865	-0.014029	0.256009
(*Z*)-**4a-TS**	-750.389835	-0.013657	0.253305
(*Z*)-**4a**	-750.442314	-0.012968	0.259769
(*E*)-**4a-TS**	-750.405615	-0.014605	0.255376
(*E*)-**4a**	-750.439131	-0.014908	0.256801
**5a-TS**	-750.407007	-0.014904	0.259280
**5a**	-750.468146	-0.014570	0.259894
**2**·(*E*)-**4a**	-1250.112603	-0.024461	0.419222
**7a-TS**	-1250.089501	-0.024904	0.418561
**6**	-922.825167	-0.017929	0.268115
**7a**	-327.282754	-0.007159	0.125369
**2·3a@1**$_2$	-8776.400107	-0.124117	1.731148
(**2·3a@1**$_2$)’	-8776.401448	-0.124285	1.733010
(*Z*)-**4a-TS@1**$_2$	-8776.368932	-0.123263	1.731335
(*Z*)-**4a@1**$_2$	-8776.418774	-0.121241	1.735901
(*E*)-**4a-TS@1**$_2$	-8776.383470	-0.122296	1.734659
(*E*)-**4a@1**$_2$	-8776.419368	-0.121131	1.737235
**5a-TS@1**$_2$	-8776.378814	-0.122866	1.734182
**5a@1**$_2$	-8776.437271	-0.121523	1.735403
**2·7a@1**$_2$	-8852.935513	-0.126723	1.761475
**7a·7a@1**$_2$	-8680.555450	-0.124196	1.749360
**3b**	-211.435327	-0.004477	0.071707
**2·3b**	-711.101061	-0.014229	0.227896

	Absolute energy B3LYP-D3(BJ)/ 6-311+G(2d,2p)[a]	***Solvation Gibbs energy***[b] ***(COSMOtherm)***	***Thermal correction to Gibbs energy (quasi-RRHO, 298.15 K)***
(*Z*)-**4b-TS**	-711.062815	-0.014238	0.224906
(*Z*)-**4b**	-711.109848	-0.012333	0.231810[c]
(*E*)-**4b-TS**	-711.078331	-0.013205	0.227956
(*E*)-**4b**	-711.112443	-0.013936	0.229451
**5b-TS**	-711.078334	-0.013950	0.231476
**5b**	-711.136200	-0.013333	0.232750
**2**·(*E*)-**4b**	-1210.786126	-0.023426	0.391112
**7b-TS**	-1210.762776	-0.023493	0.391734
**7b**	-287.955301	-0.006103	0.098386
**2·3b@1**$_2$	-8737.078372	-0.123830	1.703218
(*Z*)-**4b-TS@1**$_2$	-8737.044135	-0.123799	1.701566
(*Z*)-**4b@1**$_2$	-8737.087837	-0.121645	1.706302
(*E*)-**4b-TS@1**$_2$	-8737.057309	-0.122590	1.706432
(*E*)-**4b@1**$_2$	-8737.093352	-0.121940	1.707964
**5b-TS@1**$_2$	-8737.045143	-0.123680	1.703794
**5b@1**$_2$	-8737.100799	-0.120681	1.707026
**2·7b@1**$_2$	-8813.610282	-0.124850	1.733815
**7b·7b@1**$_2$	-8601.888660	-0.119163	1.694724

[a] Three-body dispersion correction is included.
[b] Corrections for the transfer from ideal gas to solution, +1.9 kcal/mol, are included in the reported solvation Gibbs energies.
[c] An imaginary frequency of 9*i* cm^{-1} was found in the vibrational analysis and could not be eliminated by further optimization. This was treated as real in the calculations of the thermal correction to Gibbs energy.

6. References

[S1] Hou, J.-L.; Ajami, D.; Rebek, J., Jr. *J. Am. Chem. Soc.* **2008**, *130*, 7810-7811.
[S2] Daver, H.; Harvey, J. N.; Rebek, J., Jr.; Himo, F., *J. Am. Chem. Soc.* **2017**, *139*, 15494-15503.
[S3] Brea, O.; Daver, H.; Rebek, J., Jr; Himo, F. *J. Org. Chem.* **2019**, *84*, 7354-7361.
[S4] Kleywegt, G. J.; Jones, T. A. *Acta Cryst. Sect. D* **1994**, 178-185.
[S5] Mecozzi, S.; Rebek, J., Jr. *Chem. Eur. J.* **1998**, *4*, 1016-1022.
[S6] Kim, J.-K.; Cho, Y.; Laskowski, R. A.; Ryu, S. E.; Sugihara, K.; Kim, D.-S. *Proteins* **2014**, *82*, 1829-1849. The calculations were done with a probe radius of 0.93 Å.

7. Cartesian coordinates

2

```
H  3.49043  0.26576  1.70962
C  1.70745  0.46898 -0.94359
C  2.60427 -0.14373  0.11238
O  2.96725  0.75772  1.05428
O  2.93810 -1.30775  0.15582
C  0.24836  0.27737 -0.56700
C -0.58080  1.37279 -0.32120
C -0.28785 -1.01163 -0.45110
C -1.92070  1.18681  0.02219
H -0.17970  2.37935 -0.40314
C -1.62366 -1.19174 -0.10631
H  0.35310 -1.87091 -0.62467
C -2.46430 -0.09601  0.13690
H -2.55180  2.05277  0.20402
H -2.02384 -2.19925 -0.02482
C -3.90616 -0.30266  0.53084
H -4.38272 -1.07048 -0.08718
H -3.98837 -0.63142  1.57395
H -4.48386  0.62016  0.42982
H  1.93486  1.53155 -1.05028
H  1.92936 -0.03842 -1.88672
```

3

```
C  0.91825  0.69169  0.00010
H  0.88854  1.34098  0.88274
N  2.15963 -0.00897 -0.00011
C  3.16324 -0.62420  0.00005
C -0.27949 -0.26527 -0.00004
H -0.21231 -0.91586 -0.87972
H -0.21248 -0.91605  0.87953
C -1.61229  0.48840 -0.00007
H -1.65869  1.14604 -0.87827
H -1.65867  1.14613  0.87805
C -2.81793 -0.45418  0.00004
H -2.81205 -1.10074  0.88421
H -3.75907  0.10376 -0.00031
H -2.81178 -1.10138 -0.88365
H  0.88844  1.34133 -0.88230
```

2·3a

```
C  2.20235  2.00422  0.52952
H  1.97877  1.43620  1.43734
N  3.31225  1.35894 -0.09390
C  4.11646  0.67041 -0.59597
H  3.42250 -1.23592 -0.68813
C  0.58859 -2.62294  0.01053
C  1.82916 -1.76599  0.20578
O  2.68292 -1.87122 -0.82195
O  2.01321 -1.06072  1.17937
C  0.98411  2.03167 -0.40037
H  1.24910  2.56683 -1.32010
H  0.73455  1.00372 -0.68406
C -0.22595  2.68914  0.26664
H  0.05337  3.68224  0.64404
H -0.52199  2.09169  1.13712
C -1.41118  2.81660 -0.69205
H -1.70353  1.83539 -1.07682
H -2.28002  3.25483 -0.19088
H -1.16078  3.45533 -1.54634
H  2.50093  3.01918  0.81271
C -0.67622 -1.78742  0.03556
C -1.48986 -1.69366 -1.09770
C -1.06365 -1.10187  1.19341
C -2.66941 -0.95017 -1.07040
H -1.20420 -2.21679 -2.00635
C -2.24229 -0.35884  1.21543
H -0.43116 -1.14947  2.07318
C -3.06905 -0.27309  0.08785
H -3.29165 -0.90009 -1.96033
H -2.53164  0.15645  2.12778
C -4.33925  0.54062  0.11024
H -4.66878  0.73617  1.13443
H -4.19489  1.51082 -0.37974
H -5.15111  0.02871 -0.41568
H  0.66895 -3.17783 -0.92532
H  0.57394 -3.34657  0.83406
```

(*Z*)-4a-TS

```
C  2.84292  1.97644  0.10070
H  2.91697  1.89485  1.19009
N  3.45987  0.78608 -0.45761
C  3.31546 -0.38691 -0.47724
H  3.17179 -1.56501 -0.70982
C  0.14893 -2.78993  0.50095
C  1.47988 -2.10555  0.18254
O  2.29895 -2.68104 -0.59711
O  1.69748 -0.96712  0.70315
C  1.37541  2.07145 -0.33474
H  1.33288  2.13306 -1.42895
H  0.87248  1.14695 -0.03739
C  0.66984  3.28006  0.28588
H  1.20130  4.20387  0.02029
H  0.71393  3.20078  1.38009
C -0.79154  3.37412 -0.16195
H -1.33415  2.46029  0.09890
H -1.29883  4.22140  0.30984
H -0.86172  3.50339 -1.24765
H  3.41759  2.84919 -0.21994
C -1.01077 -1.84018  0.30990
C -1.77927 -1.87040 -0.85641
C -1.30304 -0.86371  1.27110
C -2.80899 -0.95013 -1.06016
H -1.56910 -2.61970 -1.61522
C -2.33243  0.05082  1.06761
H -0.70818 -0.81801  2.17756
C -3.10110  0.02861 -0.10555
H -3.39407 -0.99462 -1.97519
H -2.54833  0.79326  1.83237
C -4.19303  1.04501 -0.33208
H -4.79661  1.19195  0.56951
H -3.77404  2.02240 -0.60215
H -4.86216  0.73917 -1.14113
H  0.05739 -3.67359 -0.13344
H  0.19875 -3.12516  1.54382
```

(*Z*)-4a

```
C  0.91052  2.45896  0.75084
H  0.75613  1.68954  1.51817
N  2.26586  2.40924  0.19709
C  2.78844  1.28859 -0.02907
H  3.76821  1.15476 -0.47729
C  1.72846 -2.23556  0.22840
C  2.61468 -1.08289 -0.21284
O  3.60442 -1.17456 -0.89643
O  2.13559  0.09696  0.28700
C -0.13944  2.28420 -0.35487
H  0.05995  3.01359 -1.14985
H -0.02383  1.28801 -0.79726
C -1.57266  2.44692  0.15678
H -1.68438  3.42959  0.63426
H -1.76179  1.69695  0.93470
C -2.60643  2.29522 -0.96110
H -2.51632  1.31406 -1.43815
H -3.62868  2.39209 -0.58136
H -2.46403  3.05685 -1.73583
H  0.78487  3.43803  1.22475
C  0.25273 -1.90682  0.19865
C -0.46438 -1.96765 -0.99954
C -0.41496 -1.48844  1.35413
C -1.81536 -1.62234 -1.03905
H  0.03757 -2.28363 -1.91049
C -1.76422 -1.14796  1.31316
H  0.12930 -1.42377  2.29228
C -2.48981 -1.21127  0.11623
H -2.35510 -1.67653 -1.98074
H -2.26470 -0.82874  2.22373
C -3.95912 -0.87175  0.08749
H -4.56298 -1.70177  0.47374
H -4.17612  0.00433  0.70604
H -4.30053 -0.65933 -0.92887
H  1.97487 -3.08310 -0.41505
H  2.03572 -2.50006  1.24767
```

(*E*)-4a-TS

```
C  3.59241 -0.65540 -0.83349
H  4.44441 -1.28472 -0.53761
N  2.42847 -1.44359 -0.77452
C  1.40654 -2.09789 -0.69489
H  1.09093 -2.97448 -1.26615
C -1.76379 -1.81308  1.23609
C -0.77799 -2.44368  0.26627
O -1.00268 -3.41268 -0.42321
O  0.39028 -1.74415  0.26467
C  3.55649  0.60923  0.04076
H  2.69183  1.21489 -0.25849
H  3.38120  0.30961  1.08142
C  4.84014  1.43606 -0.06434
H  5.01135  1.71122 -1.11409
H  5.69809  0.81633  0.23061
C  4.80318  2.70133  0.79592
H  4.66516  2.45330  1.85406
H  5.73026  3.27596  0.70501
H  3.97441  3.35361  0.49919
H  3.78465 -0.37596 -1.88061
C -2.26851 -0.48283  0.71103
C -3.50272 -0.39882  0.06147
C -1.49307  0.67769  0.82578
C -3.95651  0.81644 -0.45152
H -4.11254 -1.29161 -0.04725
C -1.94936  1.88792  0.31190
H -0.52084  0.62361  1.30551
C -3.19053  1.98049 -0.33283
H -4.92005  0.85946 -0.95264
H -1.33244  2.77765  0.41107
C -3.69122  3.30278 -0.85956
H -4.12450  3.91119 -0.05606
H -2.88069  3.88759 -1.30583
H -4.46606  3.16282 -1.61845
H -2.58668 -2.51778  1.36698
H -1.25901 -1.67449  2.19778
```

(*E*)-4a

```
C -3.72213 -0.72108 -0.23116
H -4.43875 -1.21575 -0.89926
N -2.39440 -0.89463 -0.80220
C -1.53151 -1.44590 -0.07303
H -1.65631 -1.83472  0.94026
C  2.04030 -2.14816 -0.44831
C  0.68254 -2.21387  0.21740
O  0.44004 -2.69734  1.29603
O -0.25522 -1.59768 -0.57652
C -4.07340  0.76641 -0.13376
H -3.35940  1.25519  0.54196
H -3.93029  1.22511 -1.11956
C -5.50317  1.00682  0.35788
H -5.63920  0.52627  1.33613
H -6.20843  0.51361 -0.32436
C -5.84888  2.49374  0.46606
H -5.75382  2.99188 -0.50519
H -6.87437  2.64220  0.81877
H -5.17760  3.00412  1.16564
H -3.82295 -1.19384  0.76118
C  2.63502 -0.76027 -0.28851
C  2.25181  0.28569 -1.13642
C  3.54785 -0.49422  0.73503
C  2.78123  1.56173 -0.96696
H  1.53121  0.09743 -1.92640
C  4.07606  0.78603  0.90002
H  3.85118 -1.29422  1.40480
C  3.70139  1.83530  0.05438
H  2.47741  2.35991 -1.63940
H  4.79128  0.97133  1.69707
C  4.25042  3.22751  0.24617
H  3.53327  3.86477  0.77795
H  5.17485  3.21588  0.83013
H  4.46085  3.70964 -0.71360
H  2.67302 -2.89880  0.02800
H  1.92502 -2.39575 -1.50759
```

5a-TS

```
H  1.90312  2.06381 -1.02860
C  1.52408  2.62072 -0.16139
C  0.23956  1.95067  0.32963
C  2.60354  2.66672  0.92220
C -0.82097  1.82732 -0.76579
H  3.52502  3.12998  0.55497
H -0.34992  1.39771 -1.66014
H -1.21254  2.81672 -1.04583
N -1.90114  0.96361 -0.33420
C -3.14131  0.95978 -0.68499
H -3.62887  1.68455 -1.34566
C -1.78130 -1.72423 -0.22343
C -2.35297 -0.62282  0.64731
O -2.37188 -0.53972  1.83014
O -3.81757 -0.02193 -0.18642
H  0.47461  0.94386  0.68716
H -0.17911  2.49734  1.18342
H  1.30821  3.63779 -0.51597
H  2.26577  3.23877  1.79336
H  2.84596  1.65546  1.26692
C -0.27146 -1.63486 -0.22610
C  0.41861 -1.30198 -1.39536
C  0.45851 -1.79709  0.95670
C  1.80037 -1.11878 -1.38127
H -0.13306 -1.17636 -2.32318
C  1.83990 -1.61354  0.96737
H -0.06518 -2.03529  1.87706
C  2.53407 -1.26086 -0.19729
H  2.31636 -0.85812 -2.30180
H  2.38846 -1.73814  1.89722
C  4.02208 -1.01355 -0.17458
H  4.50070 -1.36680 -1.09303
H  4.24046  0.05753 -0.08489
H  4.49762 -1.51633  0.67209
H -2.12853 -2.66869  0.21149
H -2.18175 -1.64093 -1.23300
```

5a

```
H  4.74231 -0.85926 -1.61961
C  4.84105  0.01897 -0.96790
C  3.63977  0.08270 -0.02080
C  6.17629 -0.04285 -0.22274
C  2.31469  0.14642 -0.78042
H  7.02059 -0.09048 -0.91756
H  2.18287 -0.74258 -1.39805
H  2.27572  1.03833 -1.40746
N  1.15723  0.21690  0.12759
C  0.70503  1.47607  0.50371
H -0.17894  1.45752  1.15370
C -0.59282 -0.93895  1.52542
C  0.61507 -0.98743  0.59535
O  1.08474 -2.05252  0.23111
O  1.22713  2.50968  0.13686
H  3.63309 -0.79892  0.63084
H  3.72100  0.96655  0.62335
H  4.82808  0.89560 -1.62895
H  6.31427  0.84003  0.41120
H  6.22493 -0.92586  0.42404
C -1.86631 -0.55416  0.79425
C -2.72837  0.41889  1.30556
C -2.20479 -1.18179 -0.41104
C -3.89993  0.75933  0.62879
H -2.48719  0.91229  2.24409
C -3.37358 -0.83768 -1.08355
H -1.54775 -1.94500 -0.81867
C -4.24042  0.14147 -0.57852
H -4.55733  1.51768  1.04547
H -3.61993 -1.33728 -2.01688
C -5.48821  0.53212 -1.33100
H -6.22124  1.00480 -0.67148
H -5.25835  1.24561 -2.13175
H -5.96117 -0.33715 -1.79809
H -0.67184 -1.95226  1.92785
H -0.41954 -0.26373  2.36846
```

2·(*E*)-4a

```
C  4.75948 -1.38375 -0.59289
H  3.51057 -3.00534  0.99453
C -0.64597 -1.64685  1.18448
C  0.52782 -1.81958  0.24925
O  0.57244 -1.60053 -0.92753
C -1.98806 -1.66947  0.49716
C -2.30529 -0.73338 -0.49491
C -2.95163 -2.61452  0.85543
C -3.55147 -0.75543 -1.11116
H -1.57862  0.01760 -0.77938
C -4.20531 -2.62658  0.24165
H -2.72435 -3.34655  1.62654
C -4.52482 -1.69930 -0.75427
H -3.77755 -0.01453 -1.87310
H -4.94268 -3.36735  0.53991
C -5.87250 -1.70803 -1.43375
H -6.33748 -0.71607 -1.40949
H -5.78455 -1.99534 -2.48817
H -6.55755 -2.41219 -0.95369
H -0.58246 -2.40355  1.97036
H -0.46317 -0.67666  1.66502
C  0.83368  2.74892 -0.00227
C  1.49622  1.38776  0.16043
O  1.41530  0.73247  1.19003
O  2.14174  0.98542 -0.92640
C -0.67190  2.63232  0.12506
C -1.25915  2.25089  1.33959
C -1.50546  2.87539 -0.96900
C -2.63855  2.10484  1.44432
H -0.62116  2.05045  2.19378
C -2.89039  2.73756 -0.85540
H -1.06976  3.17359 -1.91878
C -3.47913  2.33988  0.34781
H -3.07389  1.79429  2.39066
H -3.52030  2.93159 -1.71980
C -4.96476  2.11084  0.45566
H -5.50795  2.61119 -0.35119
H -5.18741  1.03860  0.39385
H -5.36033  2.47407  1.40945
H  1.22982  3.39386  0.79045
H  1.11037  3.17822 -0.96646
H  2.50692  0.06069 -0.75013
N  3.33477 -1.36716 -0.28362
C  2.89365 -2.23412  0.52301
O  1.61792 -2.32284  0.97423
H  5.27191 -2.23267 -0.11232
C  5.41541 -0.06478 -0.17222
H  4.86928  0.76477 -0.63636
H  5.30034  0.05779  0.91231
H  4.86379 -1.51345 -1.67714
C  6.89635  0.00199 -0.55429
H  6.99637 -0.13251 -1.63956
H  7.43232 -0.83770 -0.09169
C  7.55183  1.32087 -0.13832
H  7.49416  1.46468  0.94625
H  8.60793  1.35036 -0.42382
H  7.05257  2.17342 -0.61151
```

7a-TS

```
C  5.02414 -0.76821 -0.04813
N  3.63283 -1.14020  0.18532
C  3.24079 -1.76039  1.25994
H  4.00780 -2.05267  1.98527
C -0.39382 -2.26476  1.20099
C  0.85159 -2.00363  0.39811
O  1.19353 -2.40967 -0.66662
O  2.04106 -2.07339  1.57583
H  5.65364 -1.22027  0.72770
C -1.66792 -1.94582  0.45061
C -2.68856 -1.24701  1.10000
C -1.87659 -2.35968 -0.86959
C -3.88993 -0.97436  0.45232
H -2.53773 -0.90168  2.11833
C -3.07377 -2.06649 -1.51961
H -1.09259 -2.89422 -1.39423
C -4.09970 -1.36898 -0.87240
H -4.66906 -0.42985  0.97690
H -3.21304 -2.38580 -2.54953
C -5.37254 -1.00540 -1.59423
H -5.60536 -1.72179 -2.38761
H -6.22505 -0.96542 -0.90922
H -5.28461 -0.01590 -2.06031
H -0.34951 -3.33061  1.46430
H -0.33684 -1.69102  2.12458
C  5.18979  0.75387 -0.07296
H  4.47013  1.16895 -0.78796
H  4.91901  1.15589  0.91187
C  0.26925  1.80605 -0.94429
C  1.05716  0.54264 -0.58282
O  0.69746 -0.10448  0.46257
O  2.03840  0.25393 -1.30751
C -1.10877  1.95258 -0.35268
C -1.29440  2.10594  1.02847
C -2.23584  1.98983 -1.17503
```

```
C -2.56401  2.30428  1.55940
H -0.43271  2.06037  1.68667
C -3.50940  2.19232 -0.64103
H -2.11775  1.86372 -2.24803
C -3.69528  2.35634  0.73271
H -2.68329  2.42227  2.63412
H -4.37069  2.22265 -1.30342
C -5.06958  2.56799  1.31838
H -5.13847  3.52560  1.84734
H -5.83908  2.55974  0.54144
H -5.31963  1.78449  2.04368
H  0.90439  2.64711 -0.63339
H  0.23492  1.84961 -2.03633
H  2.91844 -0.71661 -0.50360
H  5.33316 -1.19491 -1.00916
C  6.61324  1.17961 -0.44239
H  6.87020  0.76556 -1.42619
H  7.32508  0.74110  0.27021
C  6.78107  2.70057 -0.46593
H  6.55846  3.13647  0.51421
H  7.80281  2.98553 -0.73461
H  6.10233  3.15822 -1.19356
```

6

```
C  0.85135 -2.61794  0.55200
C  1.83764 -1.48424  0.73553
O  2.07050 -0.91201  1.76823
C -0.52456 -2.04776  0.25766
C -1.07198 -2.10695 -1.02534
C -1.26040 -1.43066  1.27538
C -2.33518 -1.57449 -1.28265
H -0.51020 -2.57845 -1.82693
C -2.52325 -0.90821  1.01541
H -0.83638 -1.36168  2.27271
C -3.08149 -0.96622 -0.26829
H -2.74809 -1.63576 -2.28627
H -3.08892 -0.45097  1.82291
C -4.43250 -0.35823 -0.55015
H -4.35126  0.72463 -0.70710
H -4.88610 -0.78655 -1.44825
H -5.12238 -0.51033  0.28557
H  1.18085 -3.26546 -0.26310
H  0.83946 -3.19056  1.48267
C  2.71240  1.10344 -1.31225
C  3.28121 -0.04586 -0.51292
O  2.37000 -1.12084 -0.47923
O  4.35422 -0.11084  0.01052
C  1.37618  1.54605 -0.75126
C  0.19031  1.33397 -1.45776
C  1.30704  2.13867  0.51434
C -1.03422  1.72979 -0.92469
H  0.22360  0.85730 -2.43337
C  0.08024  2.52508  1.04706
H  2.21691  2.29036  1.08795
C -1.11019  2.33038  0.33624
H -1.94363  1.55981 -1.49253
H  0.04620  2.98440  2.03141
C -2.43822  2.72754  0.92893
H -2.83812  1.92490  1.55932
H -2.34829  3.62082  1.55422
H -3.17888  2.93071  0.15008
H  3.45338  1.90647 -1.30227
H  2.58787  0.76376 -2.34655
```

7a

```
C  0.45394  1.07349 -0.49651
N  1.26195  0.61989  0.63169
C  2.13888 -0.41325  0.51181
H  2.63728 -0.65523  1.47224
O  2.37527 -1.00672 -0.52780
C -0.78277  0.20323 -0.74960
H -1.28243  0.57314 -1.65477
H  1.09878  1.06425 -1.37895
H -0.44428 -0.81552 -0.97033
H  1.08153  0.98972  1.55266
C -1.77505  0.18652  0.41590
H -2.09873  1.21428  0.63372
H -1.26499 -0.17436  1.31832
C -2.99886 -0.68987  0.14039
H -2.70279 -1.72713 -0.05071
H -3.69185 -0.69034  0.98772
H -3.54720 -0.33604 -0.73976
H  0.16187  2.11108 -0.30148
```

2·3a@1$_2$

```
C -8.30763 -0.41288  3.31280
C -8.89425 -1.82018  3.24589
C -9.01855 -0.68686 -2.38395
C -8.48949 -1.97983 -2.43633
C -9.10458 -3.16445 -1.69313
C -8.47700 -3.30034 -0.30948
C -8.93150 -2.54458  0.77911
C -8.35109 -2.60375  2.04917
C -8.87995  0.67162  2.63555
C -8.97657  3.14358  1.93575
C -8.43041  3.27085  0.51616
C -8.95599  2.53316 -0.55182
C -8.44014  2.59202 -1.85072
C -8.46203  0.39080 -3.08348
C -9.04195  1.80109 -3.01170
C -7.24270 -3.44779  2.19004
C -7.40095 -4.16742 -0.07962
C -6.77041 -4.24373  1.15712
C -7.14822  2.13150  3.38361
C -7.15114 -0.15401  4.05858
C -6.55485  1.10044  4.09752
C -7.33722 -2.16337 -3.21149
C -7.33288  0.12454 -3.86739
C -6.75219 -1.13604 -3.93689
C -7.32949  3.42228 -2.05015
C -7.35334  4.11905  0.23239
C -6.78914  4.19936 -1.03529
H -5.86986 -1.30950 -4.53874
H -9.90295 -0.51230 -1.78289
H -8.81991 -4.05836 -2.24950
H -9.77176 -1.87763  0.62557
H -5.92351 -4.90090  1.30562
H -5.64921  1.26880  4.66535
H -9.78598  0.50413  2.06550
H -9.79384  1.87326 -0.35890
H -5.94272  4.84678 -1.22534
H -8.72114  2.30871 -3.92218
H -8.65239  4.03792  2.46952
H -8.53678 -2.34144  4.13486
O -6.57952 -1.19337  4.81131
O -6.60062 -3.50071  3.43298
O -6.82513  4.92703  1.24809
O -6.59422  3.40930  3.44455
O -6.76599  3.49605 -3.32751
O -6.79667  1.15809 -4.64635
O -6.93750 -5.00014 -1.10876
O -6.82081 -3.45651 -3.29901
C -5.42159 -2.83204  3.52219
C -5.39657 -1.65237  4.32039
N -4.28371 -0.97775  4.56579
N -4.35309 -3.28118  2.88085
C -3.20470 -1.47602  3.96033
C -3.24728 -2.57118  3.11087
C -1.79210 -0.97538  4.01587
C -1.87503 -2.77863  2.54787
N -1.08323 -1.80443  3.13683
O -1.33988 -0.06010  4.66683
H -0.05935 -1.82195  3.05882
O -1.51713 -3.60990  1.72995
C -5.45366  3.67810  2.75356
C -5.57060  4.56786  1.65023
C -8.33504  1.95906  2.65814
N -4.52018  5.04010  0.99835
N -4.29297  3.17384  3.14582
C -5.58934  1.63950 -4.24219
C -5.57641  2.85630 -3.50421
C -5.70421 -4.65801 -1.58906
C -5.64752 -3.74805 -2.68296
N -4.46463  3.37842 -3.00862
N -4.48620  0.97018 -4.54089
N -4.61965 -5.15430 -1.01482
N -4.51138 -3.23310 -3.12353
C -3.24179  3.63291  2.45907
C -3.42808 -3.70961 -2.50533
C -3.35001  4.57373  1.44211
C -3.36971  1.52916 -4.07018
C -3.36225  2.68721 -3.30769
C -3.47304 -4.68132 -1.51606
C -2.05866 -5.02958 -1.12610
C -1.99717 -3.31270 -2.72379
C -1.78112  3.30313  2.64933
C -1.96670  4.93296  0.97226
C -1.95284  1.05488 -4.22638
C -1.94613  2.98020 -2.90095
N -1.26163 -4.14939 -1.88885
N -1.18488  1.98314 -3.49927
N -1.11810  4.14264  1.75585
O -1.58528 -2.42352 -3.43938
O -1.66116 -5.86369 -0.35080
O -1.53800  0.09442 -4.83121
O -1.56193  3.87316 -2.17068
O -1.28100  2.49690  3.40278
O -1.62574  5.72212  0.12136
H -0.10244  4.22238  1.65585
H -0.16419  1.97782 -3.55628
H -0.24239 -4.21577 -1.90081
C  8.97094  3.46855 -1.17014
C  8.32798  2.49943 -2.15824
C  8.41699  3.21824  0.22892
H  8.64395  4.46763 -1.45932
C  8.83038  1.20938 -2.36406
C  7.19198  2.86882 -2.88990
C  8.98796  2.28803  1.10544
C  7.28535  3.91311  0.67557
C  8.25500  0.30660 -3.26150
H  9.71209  0.90291 -1.81336
C  6.57070  2.00290 -3.78457
O  6.70352  4.17466 -2.75535
C  8.47256  2.03361  2.37915
H  9.87308  1.75166  0.78493
C  6.72678  3.69252  1.93037
O  6.74861  4.90154 -0.15450
C  8.82305 -1.08509 -3.52245
C  7.11503  0.73598 -3.95144
H  5.69139  2.31036 -4.33592
C  5.51199  4.32054 -2.11064
C  9.10343  1.04620  3.36080
C  7.32447  2.74370  2.75001
H  5.85158  4.24067  2.25281
C  5.53594  4.67823 -0.73367
H  8.47793 -1.37429 -4.51539
C  8.24553 -2.11433 -2.55525
O  6.55721 -0.14768 -4.87907
N  4.38348  4.13778 -2.78265
C  8.49646 -0.34481  3.19754
H  8.82525  1.38701  4.35865
O  6.82202  2.50524  4.03154
N  4.43094  4.82416 -0.01518
C  8.79557 -2.31272 -1.28552
```

C 7.15646 -2.92197 -2.91059
C 5.37549 -0.73138 -4.54893
C 3.27987 4.30981 -2.05440
C 8.95325 -1.22532 2.20847
C 7.45890 -0.79827 4.02290
C 5.67939 1.78783 4.15590
C 3.30383 4.63447 -0.70417
C 8.32552 -3.27453 -0.38762
H 9.63425 -1.69463 -0.98683
C 6.65339 -3.89848 -2.05602
O 6.58133 -2.77044 -4.18094
C 5.38158 -2.11795 -4.22311
N 4.27004 -0.00179 -4.56088
C 1.84333 4.12714 -2.46958
C 8.41790 -2.49979 2.01317
H 9.76677 -0.90323 1.56882
C 6.86563 -2.04576 3.84510
O 6.99397 0.01213 5.07544
C 5.75610 0.54039 4.83970
N 4.54390 2.25481 3.66132
C 1.89067 4.65099 -0.19123
C 8.96679 -3.47956 0.97990
C 7.24242 -4.05716 -0.80574
H 5.81335 -4.51269 -2.35491
N 4.26962 -2.78926 -3.96241
C 3.16232 -0.68855 -4.28276
N 1.10784 4.36923 -1.29967
O 1.38951 3.83649 -3.55142
C 7.34497 -2.86736 2.83468
H 6.04472 -2.36098 4.47600
N 4.67982 -0.14447 5.19619
C 3.47752 1.51660 3.97040
O 1.52113 4.84498 0.95125
H 8.66460 -4.47287 1.31264
O 6.74498 -5.04433 0.06486
C 3.15904 -2.04930 -4.00998
C 1.75594 -0.17006 -4.17849
H 0.08712 4.31681 -1.31230
O 6.77196 -4.12913 2.63117
C 3.52883 0.38951 4.77700
C 2.07164 1.69854 3.49701
C 5.56112 -4.71866 0.65823
C 1.74243 -2.46690 -3.72496
N 0.99437 -1.28999 -3.84566
O 1.37275 0.96910 -4.33169
C 5.58447 -4.16010 1.97021
C 2.11507 -0.08910 4.99428
N 1.33000 0.77672 4.19789
O 1.70191 2.46618 2.61937
N 4.43714 -4.88328 -0.02344
O 1.32182 -3.56658 -3.44132
H -0.02567 -1.31085 -3.82345
N 4.50074 -3.66499 2.55084
O 1.70309 -0.98423 5.68710
H 0.30880 0.69840 4.17807
C 3.34565 -4.43748 0.59958
C 3.38487 -3.80300 1.83338
C 1.92656 -4.42066 0.10123
C 2.00381 -3.31329 2.15558
N 1.21524 -3.73861 1.09308
O 1.47688 -4.86983 -0.92912
O 1.63688 -2.67409 3.12334
H 0.19098 -3.67100 1.12353
O 1.06126 -0.43607 1.24355
C 0.28505 0.22423 0.56836
C -2.03202 -0.18811 -0.37636
C -2.82409 -1.32653 -0.19952
C -2.66239 1.06315 -0.36545
C -4.20553 -1.22766 -0.03756
H -2.35147 -2.29979 -0.17937
C -4.04444 1.16077 -0.20883
H -2.06510 1.96051 -0.46800
C -4.84112 0.01869 -0.04763
H -4.79535 -2.13025 0.10050
H -4.51468 2.14078 -0.22407
O 0.06436 1.52299 0.75485
H 0.61841 1.85603 1.50901
C -0.53612 -0.32994 -0.58008
H -0.26319 -1.37797 -0.70865
H -0.21799 0.21517 -1.47533
C -6.32894 0.12694 0.14357
H -6.85921 -0.68211 -0.36518
H -6.59212 0.06931 1.20388
H -6.71253 1.07454 -0.23603
C -10.51090 3.10762 1.97925
H -10.85750 3.06307 3.01519
H -10.91807 4.00912 1.51395
H -10.93377 2.24748 1.45250
C -10.57768 1.81412 -2.99145
H -10.94333 2.84413 -3.01250
H -10.96893 1.28732 -3.86593
H -10.99753 1.33518 -2.10243
C -10.63845 -3.11418 -1.64955
H -11.04134 -3.06339 -2.66462
H -11.02800 -4.01326 -1.16479
H -11.02378 -2.25149 -1.09872
C -10.42972 -1.82622 3.29335
H -10.79967 -2.85466 3.31374
H -10.77896 -1.31197 4.19273
H -10.88524 -1.33030 2.43174
C 10.50497 3.44906 -1.22452
H 10.84530 3.68847 -2.23540
H 10.91416 4.19217 -0.53475
H 10.93312 2.47925 -0.95560
C 10.63737 1.03306 3.28800
H 11.03012 2.03806 3.46437
H 11.04110 0.36268 4.05123
H 11.01786 0.69516 2.32013
C 10.35928 -1.10201 -3.54601
H 10.71860 -2.10339 -3.79749
H 10.72929 -0.40001 -4.29808
H 10.80634 -0.82349 -2.58745
C 10.50221 -3.46224 0.91588
H 10.92078 -3.67812 1.90256
H 10.85512 -4.22187 0.21345
H 10.90583 -2.49959 0.58990
C 4.83270 0.12283 1.02979
C 5.29712 -1.01219 0.11141
C 4.22744 -1.51082 -0.86614
C 4.55563 1.45842 0.33244
N 3.42654 1.40524 -0.54268
H 6.19311 -0.70075 -0.44139
H 4.48299 -2.49118 -1.26796
H 5.42655 1.76266 -0.25855
H 4.36180 2.23984 1.07189
H 3.93098 -0.18196 1.57322
H 5.61434 0.32057 1.77035
H 5.62150 -1.83390 0.75450
H 3.24886 -1.57824 -0.37906
H 4.11633 -0.82668 -1.70691
C 2.48912 1.31122 -1.24185

(2·3a@1$_2$)’

C 8.85039 -1.55470 3.23359
C 8.26626 -0.14453 3.22309
C 8.27913 -2.42115 2.11408
H 8.51893 -2.01157 4.16636
C 8.80952 0.88113 2.44551
C 7.16516 0.18378 4.02517
C 8.82860 -2.45036 0.82892
C 7.18479 -3.26160 2.35504
C 8.31774 2.19054 2.45164
H 9.65349 0.65176 1.80546
C 6.65297 1.47493 4.08797
O 6.59445 -0.82477 4.81384
C 8.34830 -3.28380 -0.18743
H 9.67704 -1.81095 0.61438
C 6.65265 -4.09348 1.37576
O 6.64466 -3.28606 3.64720
C 8.93192 3.27287 1.57161
C 7.23367 2.46213 3.29634
H 5.80859 1.70435 4.72558
C 5.42081 -1.35584 4.36577
C 8.98949 -3.31890 -1.57282
C 7.24588 -4.09390 0.11684
H 5.80258 -4.72908 1.58892
C 5.44325 -2.65871 3.78947
H 8.61596 4.22661 1.99527
C 8.36368 3.22860 0.15451
O 6.72462 3.77146 3.36759
C 8.40952 -2.20568 -2.43773
H 8.68292 -4.25962 -2.03030
O 6.76647 -4.97133 -0.86566
C 8.89940 2.43533 -0.86286
C 7.25601 4.01973 -0.17703
C 5.51827 3.95149 2.74954
C 3.21774 -1.26833 4.01163
C 8.94060 -0.91028 -2.44947
C 7.28461 -2.44731 -3.23586
C 5.56902 -4.65204 -1.43579
C 3.23243 -2.55812 3.50029
C 8.40885 2.44300 -2.17643
H 9.73438 1.78490 -0.62802
C 6.75605 4.09768 -1.46824
O 6.67846 4.78039 0.84142
C 5.50471 4.35621 1.38313
C 8.38102 0.13210 -3.19366
H 9.82774 -0.70989 -1.86067
C 6.67977 -1.44337 -3.98487
O 6.78103 -3.75114 -3.28936
C 5.57653 -3.99134 -2.69620
C 8.98968 1.53439 -3.25660
C 7.35353 3.31963 -2.45648
H 5.91339 4.73570 -1.70066
C 3.28769 3.80422 2.70279
C 7.22111 -0.16586 -3.92213
H 5.79883 -1.64803 -4.57902
C 3.33502 -4.58266 -1.43245
H 8.67603 1.95812 -4.21152
O 6.85872 3.41865 -3.77027
C 3.29273 4.05469 1.33665
O 6.63682 0.86916 -4.65311
C 3.34238 -3.91117 -2.64920
C 5.61372 2.86576 -3.88779
C 5.52111 1.47279 -4.16860
C 3.40419 2.93416 -3.59191
C 3.34798 1.54892 -3.66832
C 10.52545 1.51247 -3.24378
H 10.90056 0.89095 -4.06128
H 10.94049 1.11885 -2.31183
C 10.46798 3.24609 1.58769
H 10.86377 4.07707 0.99764
H 10.88419 2.32237 1.17639
C 10.38716 -1.55032 3.24381
H 10.75430 -0.97696 4.09917
H 10.82040 -1.10942 2.34178
C 10.52373 -3.30687 -1.51874
H 10.88287 -4.15700 -0.93287
H 10.93100 -2.39992 -1.06352
H 10.93509 -3.38238 -2.52894
H 10.76321 -2.57376 3.32317
H 10.83231 3.34283 2.61379
H 10.91244 2.52671 -3.37190
C -9.02743 1.20120 3.33018
C -8.45070 -0.20567 3.19545
C -8.44212 2.15776 2.28625
H -8.69317 1.57442 4.29928
C -9.00674 -1.15814 2.32832
C -7.31488 -0.59091 3.92019
C -8.98861 2.32557 1.00955
C -7.32365 2.94218 2.59508
C -8.45451 -2.42697 2.13286
H -9.89828 -0.88933 1.77399

C -6.71523 -1.83368 3.74836
O -6.76185 0.28534 4.86661
C -8.48963 3.25028 0.08290
H -9.83492 1.71116 0.72380
C -6.81552 3.90231 1.73374
O -6.71789 2.76318 3.84059
C -9.05397 -3.47660 1.19281
C -7.28111 -2.71618 2.84166
H -5.82427 -2.09927 4.30190
C -5.55319 0.80991 4.50906
C -9.04553 3.37534 -1.33165
C -7.41774 4.05159 0.49235
H -5.96411 4.50734 2.01566
C -5.54716 2.07399 3.85749
H -8.75317 -4.44754 1.58935
C -8.43694 -3.37355 -0.20139
O -6.71279 -3.97404 2.65578
C -8.36136 2.36781 -2.25376
H -8.75818 4.36422 -1.69059
O -6.94316 5.04784 -0.37286
C -8.90160 -2.44034 -1.14109
C -7.35437 -4.17672 -0.58657
C -5.55910 -4.09416 1.95194
C -3.33944 0.69686 4.26209
C -8.84128 1.06591 -2.41826
C -7.21206 2.71579 -2.97671
C -5.68942 4.83566 -0.86228
C -3.35770 1.86070 3.50494
C -8.30873 -2.25443 -2.39299
H -9.74863 -1.81900 -0.87277
C -6.71712 -4.01451 -1.81210
O -6.86215 -5.17673 0.27025
C -5.62174 -4.87719 0.76523
C -8.24964 0.12878 -3.27304
H -9.72170 0.76795 -1.86152
C -6.59459 1.83234 -3.85365
O -6.71685 4.01659 -2.84792
C -5.57131 4.21943 -2.14126
C -8.83076 -1.27297 -3.44262
C -7.18750 -3.04196 -2.67822
H -5.85932 -4.62103 -2.07131
C -3.35671 -3.89834 1.67546
C -7.12108 0.55066 -3.98932
H -5.71333 2.12790 -4.40867
C -3.46447 4.87680 -0.68496
H -8.46106 -1.63646 -4.40234
O -6.53847 -2.83659 -3.89839
C -3.39634 -4.79367 0.61727
O -6.50688 -0.32811 -4.89566
C -3.35392 4.21857 -1.90116
C -5.36693 -2.14878 -3.84561
C -5.33026 -0.86413 -4.45575
C -3.20488 -1.95284 -3.34469
C -3.14046 -0.75365 -4.03964
C -10.36605 -1.26679 -3.51269
H -10.70356 -0.59936 -4.31006
H -10.83462 -0.93377 -2.58234
C -10.58846 -3.44145 1.16537
H -10.96921 -4.25029 0.53629
H -10.98752 -2.50302 0.77033
C -10.56331 1.21772 3.33328
H -10.94415 0.57261 4.12962
H -10.99671 0.87024 2.39119
C -10.57732 3.28916 -1.38588
H -11.01660 4.07360 -0.76390
H -10.96615 2.33151 -1.02896
H -10.92579 3.42384 -2.41337
H -10.92610 2.23450 3.50518
H -10.98629 -3.56938 2.17570
H -10.73375 -2.27473 -3.72240
C 2.00963 3.45453 -3.37429
C 1.94877 1.11502 -3.32555
C 1.83163 -2.92142 3.09384
C 1.83194 -0.71235 3.85072
C -1.93288 -3.46792 1.88455
C -1.98006 -5.07636 0.18659
C -1.85033 -2.24743 -2.77019
C -1.72236 -0.25590 -3.98095
C -1.91727 0.22321 4.36278
C -1.96404 2.12703 3.01048
C -2.07748 5.09600 -0.13462
C 1.86225 3.58699 3.13622
C 1.88253 3.92822 0.82607
C -1.90487 3.91535 -2.14527
C 1.92680 -3.52601 -2.98992
C 1.91339 -4.65417 -0.93746
N 1.16713 -4.01147 -1.92256
N 1.22408 2.29372 -3.24788
N -1.04401 -1.21281 -3.20996
N -1.19157 -4.24359 1.01164
N 1.08424 -1.76342 3.33844
N 1.11553 3.70404 1.94887
N -1.18401 1.14269 3.58761
N -1.22930 4.48162 -1.07920
O 1.56441 -0.01740 -3.09574
O 1.61327 4.59311 -3.33050
O 1.49887 4.00144 -0.33124
O -1.72352 5.66252 0.86947
O -1.43273 3.25663 -3.05460
O -1.23777 0.74270 -4.46064
O -1.53474 -3.19347 -2.07081
O -1.57237 -5.84497 -0.64770
O 1.50852 -2.91570 -3.94829
O 1.46518 0.42423 4.07702
O 1.40888 -3.96675 2.65644
O 1.49791 -5.12229 0.09985
O -1.45893 -0.72476 4.95481
O -1.53791 -2.60050 2.64465
O -1.60305 3.00755 2.24649
O 1.41077 3.36873 4.23370
H 0.20508 2.37232 -3.18908
H -0.21614 4.39740 -0.93600
H 0.10454 3.51960 1.94319
H -0.15842 1.08363 3.55853
H 0.07299 -1.77337 3.19686
H -0.17183 -4.30806 1.01536
H 0.15521 -3.84779 -1.86681
H -0.04577 -1.11739 -3.02434
N 4.40510 3.71572 3.42807
N 4.39817 4.35973 0.65332
N 4.40083 0.78978 -3.98959
N 4.55068 3.61628 -3.65300
N 4.46356 -3.60489 -3.30521
N 4.44711 -4.95713 -0.79863
N 4.30618 -0.64833 4.47015
N 4.34850 -3.27512 3.36869
N -4.43462 -3.54665 2.38399
N -4.54026 -5.27084 0.11301
N -4.44450 0.12725 4.75037
N -4.45856 2.58712 3.30427
N -4.31348 -2.68581 -3.24799
N -4.20902 -0.16987 -4.58622
N -4.64027 5.18873 -0.13452
N -4.40061 3.88446 -2.66074
C 5.26590 0.49130 -0.79143
C 5.55171 0.80916 0.67597
C 4.32056 0.72923 1.57681
C 4.99435 -0.98090 -1.10635
N 3.83384 -1.50239 -0.45599
C -0.78247 1.01285 -0.50334
H 6.34652 0.15195 1.04912
H 4.50748 1.18476 2.54779
H 5.84827 -1.59615 -0.80410
H 4.83910 -1.10894 -2.17921
H -0.52362 1.03072 -1.56458
H -0.65180 2.02449 -0.11454
C -2.21969 0.57279 -0.34288
C -2.64988 -0.14832 0.77658
C -3.17279 0.95616 -1.29113
C -3.99806 -0.44682 0.95976
H -1.92979 -0.49656 1.50644
C -4.52138 0.65833 -1.10475
H -2.86680 1.48943 -2.18342
C -4.95760 -0.03815 0.02416
H -4.30434 -1.00643 1.83886
H -5.24437 0.97149 -1.84903
C -6.40930 -0.36902 0.21341
H -6.59407 -1.43230 0.03972
H -6.73560 -0.14727 1.22972
H -7.04311 0.19388 -0.47450
H 4.42035 1.09428 -1.13979
H 6.13272 0.76590 -1.39928
H 5.96549 1.81830 0.70948
H 3.45472 1.21623 1.12315
H 4.02864 -0.30172 1.75945
C 2.86648 -1.90319 0.06133
H 1.00030 -1.58751 0.13936
C 0.25223 0.14973 0.17791
O 0.18544 -1.13547 -0.19585
O 1.08346 0.57956 0.95981

(*Z*)-**4a-TS@1**$_2$

C -8.96886 -3.27313 -1.59826
C -8.35567 -2.15551 -2.43792
C -8.37119 -3.27263 -0.19522
H -8.66150 -4.21030 -2.06298
C -8.87690 -0.86025 -2.46664
C -7.21979 -2.40068 -3.22046
C -8.87227 -2.44922 0.82120
C -7.28440 -4.09658 0.12423
C -8.32798 0.16905 -3.24094
H -9.74445 -0.63837 -1.85671
C -6.65351 -1.42656 -4.03356
O -6.69199 -3.69383 -3.21010
C -8.33601 -2.42058 2.11129
H -9.72265 -1.81476 0.59926
C -6.68817 -4.07694 1.38132
O -6.80526 -5.00099 -0.83691
C -8.90924 1.57865 -3.22408
C -7.21528 -0.15230 -4.03046
H -5.79065 -1.64833 -4.64867
C -5.52556 -3.92311 -2.54811
C -8.93403 -1.58756 3.24580
C -7.21913 -3.23193 2.34732
H -5.83436 -4.70509 1.59998
C -5.57972 -4.70187 -1.35720
H -8.56983 2.05749 -4.14323
C -8.34374 2.42021 -2.07836
O -6.65501 0.82849 -4.86445
C -8.36249 -0.17373 3.26352
H -8.60573 -2.06015 4.17196
O -6.66329 -3.20918 3.63005
C -8.90200 2.45129 -0.79681
C -7.23565 3.24738 -2.30060
C -5.45620 1.32436 -4.43816
C -3.30402 -3.82921 -2.35288
C -8.91597 0.85487 2.49109
C -7.25751 0.15139 4.06092
C -5.49204 -2.53669 3.78148
C -3.34677 -4.68602 -1.26163
C -8.42943 3.29121 0.22131
H -9.74120 1.80001 -0.58024
C -6.75460 4.12921 -1.34523
O -6.62851 3.19846 -3.55888
C -5.45782 2.51743 -3.66018
C -8.39408 2.15051 2.45872
H -9.78865 0.63320 1.88830
C -6.68966 1.42164 4.05251
O -6.71852 -0.82348 4.91616
C -5.50901 -1.32646 4.53215
C -9.01883 3.27018 1.62801
C -7.37102 4.14914 -0.09832
H -5.91285 4.77513 -1.55807

```
C -3.23724  1.19945 -4.20291
C -7.24635  2.38938  3.22726
H -5.82046  1.64146  4.65872
C -3.29260 -2.30973  3.50621
H -8.71777  4.20589  2.10033
O -6.89847  5.05545  0.86515
C -3.25904  2.29195 -3.34506
O -6.68636  3.66934  3.18562
C -3.28937 -1.18659  4.32230
C -5.65281  4.72818  1.32362
C -5.55628  3.86872  2.45431
C -3.43397  4.64981  1.08946
C -3.36009  3.68393  2.08368
C -10.55388  3.22377  1.62808
H -10.93128  3.25499  2.65363
H -10.95506  2.32249  1.15630
C -10.44574  1.58434 -3.24201
H -10.81432  2.61149 -3.30538
H -10.88556  1.13235 -2.34879
C -10.50408 -3.23994 -1.59437
H -10.88308 -3.29352 -2.61844
H -10.91284 -2.33386 -1.13878
C -10.47014 -1.60908  3.23678
H -10.82945 -2.63977  3.29665
H -10.90006 -1.16371  2.33534
H -10.85774 -1.05469  4.09565
H -10.89394 -4.09373 -1.03396
H -10.81301  1.02824 -4.10865
H -10.95254  4.08404  1.08395
C  9.00477 -1.18615 -3.41384
C  8.42981 -2.21633 -2.44512
C  8.41855  0.20716 -3.16783
H  8.66803 -1.48325 -4.40814
C  8.97064 -2.40718 -1.16509
C  7.31662 -2.99604 -2.78734
C  8.98622  1.13816 -2.29193
C  7.26864  0.61459 -3.85512
C  8.42806 -3.29034 -0.22779
H  9.84158 -1.82624 -0.88443
C  6.72704 -3.87374 -1.88453
O  6.76001 -2.90966 -4.07470
C  8.47302  2.43057 -2.12130
H  9.86066  0.84838 -1.72064
C  6.74281  1.89341 -3.75654
O  6.66207 -0.30651 -4.70919
C  9.02533 -3.50790  1.16494
C  7.27636 -3.98639 -0.61704
H  5.84997 -4.44354 -2.16192
C  5.53833 -2.29653 -4.09594
C  9.05090  3.42088 -1.11717
C  7.36521  2.78815 -2.89767
H  5.86650  2.18084 -4.32182
C  5.50897 -0.88792 -4.29186
H  8.70601 -4.50235  1.48016
C  8.43243 -2.52876  2.17629
O  6.69219 -4.85415  0.30562
C  8.40557  3.22324  0.25330
H  8.75262  4.41365 -1.45575
O  6.88334  4.10217 -2.81894
C  8.94485 -1.23323  2.34087
C  7.32485 -2.87869  2.96059
C  5.53214 -4.46873  0.89969
C  3.32581 -2.27940 -3.81073
C  8.89922  2.29894  1.17729
C  7.28790  3.97280  0.64529
C  5.65137  4.24092 -2.25689
C  3.32825 -0.89117 -3.84222
C  8.37643 -0.29291  3.20443
H  9.81423 -0.94601  1.76062
C  6.70708 -1.97104  3.81406
O  6.80286 -4.18132  2.89857
C  5.57418 -4.24117  2.30252
C  8.36100  2.12496  2.45736
H  9.74709  1.68766  0.89112
C  6.72788  3.85801  1.91105
O  6.75614  4.90333 -0.25336
C  5.58617  4.59758 -0.87945
C  8.94768  1.10675  3.43143
C  7.22925 -0.69119  3.90057
H  5.83151 -2.25519  4.38254
C  3.33662 -4.08353  0.92824
C  7.27612  2.94142  2.80395
H  5.87392  4.46025  2.19455
C  3.42336  4.14922 -2.32994
H  8.61431  1.41144  4.42437
O  6.63195  0.24329  4.74729
C  3.35616 -3.99626  2.31361
O  6.71735  2.85633  4.08991
C  3.36322  4.43520 -0.97311
C  5.47684  0.82568  4.33589
C  5.50192  2.23119  4.11655
C  3.29364  0.81807  3.90009
C  3.28944  2.20463  3.83074
C 10.48396  1.11770  3.43802
H 10.85063  2.12207  3.66594
H 10.91830  0.81798  2.48003
C 10.56075 -3.49522  1.15483
H 10.94326 -3.71916  2.15417
H 10.97850 -2.53111  0.85113
C 10.54053 -1.17964 -3.42903
H 10.91671 -2.17664 -3.67337
H 10.97653 -0.88957 -2.46878
C 10.58491  3.39315 -1.05768
H 11.00009  3.60498 -2.04666
H 10.98433  2.42821 -0.73326
H 10.94769  4.15092 -0.35814
H 10.90530 -0.47529 -4.18136
H 10.93805 -4.25026  0.46000
H 10.85956  0.42840  4.19897
C -2.03942  4.93256  0.59817
C -1.94009  3.18959  2.15483
C -1.93630 -4.88877 -0.76704
C -1.89525 -3.33561 -2.51478
C  1.92666 -3.86349  0.45864
C  1.93884 -3.81783  2.79196
C  1.90772  0.32247  3.61602
C  1.87697  2.65934  3.58205
C  1.90728 -2.74372 -3.62957
C  1.93586 -0.40535 -3.56015
C  2.01866  3.96581 -2.84106
C -1.81086  0.73764 -4.33657
C -1.86098  2.53152 -2.84219
C  1.92571  4.37921 -0.54278
C -1.87487 -0.67126  4.39677
C -1.91045 -2.50800  2.96137
N -1.14505 -1.52600  3.55121
N -1.23196  4.02091  1.30450
N  1.13884  1.46585  3.48199
N  1.17826 -3.75378  1.60862
N -1.15780 -4.03694 -1.57709
N -1.08404  1.59844 -3.49853
N  1.16709 -1.55229 -3.47803
N  1.20941  4.13904 -1.70083
O -1.52138  2.23158  2.78047
O -1.65461  5.75254 -0.19880
O -1.49932  3.37237 -2.03767
O  1.62348  3.72555 -3.95573
O  1.48541  4.46698  0.59087
O  1.43101  3.77662  3.47226
O  1.54523 -0.83507  3.50804
O  1.50261 -3.76117  3.91552
O -1.42487  0.26370  5.01513
O -1.51624 -2.43991 -3.24732
O -1.52364 -5.59451  0.11976
O -1.56123 -3.33340  2.13167
O  1.45328 -3.86176 -3.62835
O  1.53763 -3.78991 -0.69707
O  1.57488  0.74995 -3.40685
O -1.34919 -0.15567 -5.00558
H -0.21223  4.04172  1.19460
H  0.19228  4.00095 -1.73524
H -0.07201  1.46627 -3.38214
H  0.14628 -1.59227 -3.40601
H -0.15051 -3.91446 -1.41011
H  0.16407 -3.59410  1.63893
H -0.13803 -1.38041  3.38744
H  0.12847  1.49183  3.32855
N -4.34154  0.67370 -4.73919
N -4.36700  2.98216 -3.06668
N -4.41381  3.30241  2.81154
N -4.59074  5.16512  0.66591
N -4.40359 -0.66168  4.83719
N -4.38750 -3.01860  3.23235
N -4.38774 -3.44424 -3.02956
N -4.49075 -5.11908 -0.72699
N  4.41961 -4.34527  0.19203
N  4.48413 -4.04453  3.02800
N  4.44142 -3.01051 -3.89850
N  4.41528 -0.16473 -4.10990
N  4.38119  0.09897  4.17796
N  4.40294  2.93809  3.90085
N  4.57285  4.03386 -2.99764
N  4.44019  4.67398 -0.22008
C -5.73906 -0.28338 -0.62587
C -5.61136  0.71319  0.53192
C -4.53202  0.34893  1.55370
C -4.44000 -0.50985 -1.39838
N -3.53157 -1.38513 -0.68810
C  0.75915  1.14322  0.03949
H -5.41413  1.71189  0.11990
H -4.62765  0.94098  2.46109
H -3.90415  0.42980 -1.56067
H -4.62980 -0.98452 -2.36202
H  0.55370  1.82575  0.86924
H  0.59942  1.72503 -0.87178
C  2.18983  0.66736  0.10564
C  2.59480 -0.56593 -0.42031
C  3.17476  1.52140  0.61282
C  3.94271 -0.91427 -0.46307
H  1.85170 -1.25985 -0.78987
C  4.52424  1.17126  0.56915
H  2.89178  2.48043  1.03171
C  4.93145 -0.04757  0.02252
H  4.23252 -1.87357 -0.88388
H  5.26811  1.85855  0.95893
C  6.38223 -0.43439 -0.02252
H  6.62617 -1.13608  0.77979
H  6.63058 -0.92396 -0.96542
H  7.03627  0.43337  0.09048
H -6.10986 -1.24781 -0.26015
H -6.48041  0.08436 -1.33889
H -6.58446  0.77311  1.02551
H -4.61578 -0.70640  1.82570
H -3.52610  0.52150  1.16462
C -2.45382 -1.43418 -0.21714
H -1.38529 -1.58530  0.31875
C -0.34029  0.09735  0.07588
O -0.14488 -1.01473  0.66335
O -1.44179  0.40386 -0.47861
```

(*Z*)-**4a**@**1**$_2$

```
C -8.93247 -2.53208 -2.56446
C -8.32631 -1.20112 -3.00065
C -8.34930 -2.98192 -1.23020
H -8.61487 -3.26971 -3.30195
C -8.85739  0.03122 -2.61688
C -7.19469 -1.17647 -3.82542
C -8.87224 -2.53784 -0.01028
C -7.26473 -3.86640 -1.17957
C -8.33461  1.25569 -3.04864
H -9.71656  0.04149 -1.95650
C -6.65235  0.00720 -4.30784
O -6.64934 -2.40183 -4.21208
```

C	-8.36228	-2.93935	1.22639
H	-9.72051	-1.86339	-0.02689
C	-6.69615	-4.26891	0.02475
O	-6.76459	-4.40081	-2.37702
C	-8.92638	2.58116	-2.58417
C	-7.23947	1.20826	-3.91997
H	-5.79191	-0.00266	-4.96438
C	-5.48747	-2.81122	-3.63466
C	-8.98142	-2.50807	2.55515
C	-7.25158	-3.79140	1.20511
H	-5.84463	-4.93694	0.03994
C	-5.54076	-3.93283	-2.75906
H	-8.59725	3.33226	-3.30310
C	-8.35099	3.00536	-1.23294
O	-6.71257	2.40797	-4.43384
C	-8.40299	-1.17785	3.02384
H	-8.67352	-3.25188	3.29049
O	-6.72801	-4.20551	2.43540
C	-8.90906	2.62761	-0.00855
C	-7.22495	3.83607	-1.18259
C	-5.50000	2.74206	-3.90592
C	-3.27373	-2.76369	-3.39527
C	-8.92966	0.04805	2.59970
C	-7.31383	-1.13545	3.90245
C	-5.55448	-3.64287	2.82587
C	-3.31216	-3.93432	-2.65022
C	-8.41829	3.07891	1.22405
H	-9.76144	1.95793	-0.01165
C	-6.72569	4.34947	0.00446
O	-6.62854	4.19036	-2.39650
C	-5.47267	3.57279	-2.74703
C	-8.39801	1.28096	2.98615
H	-9.78961	0.03908	1.94040
C	-6.73814	0.06430	4.30692
O	-6.79712	-2.33970	4.40553
C	-5.58343	-2.71321	3.90551
C	-9.00781	2.61371	2.55127
C	-7.34360	3.97418	1.19294
H	-5.87086	5.01272	0.00918
C	-3.28109	2.52553	-3.77937
C	-7.26731	1.24888	3.81310
H	-5.87796	0.07082	4.96358
C	-3.33967	-3.41297	2.71353
H	-8.70004	3.34763	3.29696
O	-6.85799	4.50929	2.39894
C	-3.27921	3.21043	-2.57183
O	-6.68725	2.46863	4.17902
C	-3.35632	-2.56809	3.81532
C	-5.62451	4.02523	2.72822
C	-5.55125	2.85771	3.54090
C	-3.40908	3.96717	2.46851
C	-3.36040	2.74061	3.11753
C	-10.54346	2.58260	2.53932
H	-10.92167	2.29503	3.52395
H	-10.95152	1.87656	1.81066
C	-10.46262	2.57893	-2.59049
H	-10.84010	3.56659	-2.31246
H	-10.89257	1.85424	-1.89349
C	-10.46833	-2.50991	-2.56539
H	-10.83910	-2.23908	-3.55766
H	-10.88768	-1.79576	-1.85115
C	-10.51703	-2.50788	2.51445
H	-10.88393	-3.49986	2.23788
H	-10.92685	-1.79394	1.79450
H	-10.91775	-2.25024	3.49856
H	-10.85591	-3.49896	-2.30702
H	-10.83192	2.33350	-3.58977
H	-10.93452	3.57306	2.29173
C	9.02765	0.01047	-3.60744
C	8.45646	-1.26797	-2.99950
C	8.42156	1.25621	-2.95418
H	8.70110	0.02584	-4.64827
C	8.99622	-1.83977	-1.83791
C	7.34475	-1.90709	-3.56385
C	8.97141	1.87658	-1.82762
C	7.26914	1.84317	-3.49175
C	8.45049	-2.96483	-1.21429
H	9.87232	-1.37761	-1.39788
C	6.74688	-3.01194	-2.96738
O	6.80397	-1.44076	-4.77356
C	8.44042	3.04562	-1.26697
H	9.84698	1.43379	-1.36677
C	6.72513	3.02115	-3.00351
O	6.67871	1.22756	-4.59652
C	9.05102	-3.60813	0.03748
C	7.29310	-3.50264	-1.79211
H	5.87216	-3.47238	-3.40771
C	5.57463	-0.86422	-4.62850
C	9.00606	3.67869	0.00017
C	7.33060	3.61277	-1.90352
H	5.84816	3.46042	-3.45986
C	5.52714	0.53886	-4.39586
H	8.74557	-4.65533	0.01727
C	8.44615	-3.01009	1.30535
O	6.71744	-4.61320	-1.17886
C	8.36149	3.05477	1.23735
H	8.69613	4.72421	-0.01236
O	6.82686	4.83137	-1.42788
C	8.93613	-1.81953	1.86149
C	7.35583	-3.61118	1.94864
C	5.56010	-4.46177	-0.48513
C	3.36208	-0.96137	-4.36823
C	8.87118	1.90051	1.83861
C	7.22262	3.62247	1.82428
C	5.59201	4.76912	-0.85784
C	3.34302	0.37579	-3.99468
C	8.36855	-1.20927	2.98302
H	9.78862	-1.34458	1.38949
C	6.74169	-3.03231	3.05433
O	6.85193	-4.83870	1.48761
C	5.61696	-4.73158	0.91076
C	8.31954	1.32211	2.98752
H	9.74177	1.43142	1.39557
C	6.64477	3.10215	2.97577
O	6.68873	4.78279	1.25526
C	5.52236	4.67887	0.56169
C	8.91989	0.06760	3.61738
C	7.24564	-1.83494	3.53719
H	5.88318	-3.50091	3.51756
C	3.35626	-4.17119	-0.31509
C	7.20232	1.96021	3.54299
H	5.77300	3.56829	3.41731
C	3.36602	4.65855	-0.96561
H	8.58233	0.05936	4.65450
O	6.64431	-1.22966	4.64323
C	3.39302	-4.58464	1.00947
O	6.63610	1.46045	4.72555
C	3.30393	4.51353	0.41316
C	5.46007	-0.59854	4.43370
C	5.44229	0.81880	4.56357
C	3.26667	-0.57220	4.04845
C	3.22918	0.79587	4.27651
C	10.45599	0.09387	3.63400
H	10.81057	0.99142	4.14763
H	10.89495	0.09172	2.63258
C	10.58624	-3.57408	0.04011
H	10.97010	-4.10558	0.91482
H	10.99096	-2.55882	0.06905
C	10.56314	0.03655	-3.60528
H	10.95317	-0.83555	-4.13676
H	10.98961	0.02648	-2.59858
C	10.54027	3.65101	0.05363
H	10.95322	4.16165	-0.82037
H	10.95048	2.63765	0.06657
H	10.89418	4.16047	0.95381
H	10.92348	0.93929	-4.10555
H	10.97473	-4.05818	-0.85986
H	10.83851	-0.78379	4.16172
C	-2.01110	4.35442	2.07421
C	-1.95335	2.21469	3.02096
C	-1.90521	-4.28189	-2.24754
C	-1.86853	-2.24536	-3.39137
C	1.93167	-3.84474	-0.67065
C	1.97765	-4.65204	1.51920
C	1.88441	-1.04011	3.70378
C	1.80296	1.25523	4.13706
C	1.95462	-1.48605	-4.31612
C	1.94414	0.73312	-3.58439
C	1.96328	4.59616	-1.51313
C	-1.86862	2.11132	-4.08982
C	-1.88647	3.18122	-2.01229
C	1.87345	4.28182	0.80085
C	-1.93835	-2.15244	4.10597
C	-1.92700	-3.52450	2.21878
N	-1.17467	-2.79353	3.11635
N	-1.22446	3.25334	2.46379
N	1.09166	0.09444	3.78946
N	1.19466	-4.20834	0.43444
N	-1.12401	-3.22212	-2.75740
N	-1.12597	2.54779	-2.97355
N	1.19416	-0.40007	-3.83547
N	1.15961	4.36412	-0.37944
O	-1.55430	1.09718	3.29969
O	-1.60780	5.36604	1.55411
O	-1.51290	3.59738	-0.92638
O	1.56594	4.71389	-2.64635
O	1.44124	4.01513	1.90883
O	1.33283	2.36075	4.27084
O	1.54216	-2.16832	3.40456
O	1.55900	-5.01383	2.59088
O	-1.50567	-1.42371	4.96825
O	-1.48730	-1.15486	-3.78686
O	-1.48563	-5.22074	-1.61593
O	-1.53894	-4.09681	1.21323
O	1.52096	-2.57352	-4.60978
O	1.52924	-3.34229	-1.70919
O	1.56186	1.78567	-3.09834
O	-1.41962	1.54033	-5.05292
H	-0.20336	3.30827	2.40028
H	0.14830	4.20909	-0.45305
H	-0.11083	2.39045	-2.92567
H	0.17123	-0.48417	-3.75969
H	-0.11972	-3.17298	-2.56025
H	0.17751	-4.11216	0.53346
H	-0.15565	-2.66439	3.06516
H	0.07787	0.12389	3.67548
N	-4.40009	2.24717	-4.45258
N	-4.36974	3.76641	-2.03923
N	-4.42467	2.17938	3.69838
N	-4.55051	4.61651	2.23027
N	-4.48212	-2.18913	4.42400
N	-4.43391	-3.97818	2.20411
N	-4.35342	-2.19147	-3.92621
N	-4.45141	-4.52747	-2.29397
N	4.43709	-4.12329	-1.09996
N	4.53220	-4.83858	1.66100
N	4.48839	-1.61972	-4.65800
N	4.42073	1.16285	-4.02317
N	4.37871	-1.30226	4.13582
N	4.32276	1.52550	4.51164
N	4.51650	4.77894	-1.63148
N	4.37678	4.52814	1.20790
N	-3.38087	-1.18834	-0.96876
C	-2.22411	-1.24594	-0.48275
O	-1.39557	-0.13329	-0.32857
C	-0.19507	-0.33981	0.31178
O	0.09822	-1.38747	0.83502
C	0.67310	0.89325	0.26953
H	-1.77584	-2.17515	-0.15199
H	0.44119	1.46739	1.17161
H	0.36827	1.51188	-0.57833
C	2.14759	0.56410	0.21393
C	2.62305	-0.41587	-0.66692
C	3.07326	1.25508	0.99742
C	3.98430	-0.67911	-0.77590
H	1.92631	-0.99380	-1.26188
C	4.44040	0.99521	0.88114
H	2.73651	2.00851	1.69910

```
C  4.91878  0.02962 -0.00707
H  4.32481 -1.45092 -1.46059
H  5.14080  1.55412  1.49210
C  6.38633 -0.27400 -0.11446
H  6.63759 -1.18355  0.43879
H  6.68162 -0.43756 -1.15209
H  6.99814  0.53498  0.28969
C -5.40529  0.19487 -0.82590
C -5.45838  0.63551  0.64245
C -4.62556 -0.23460  1.58691
C -3.98684  0.06716 -1.39336
H -5.12614  1.68064  0.71066
H -4.84008 -0.00552  2.62771
H -3.38524  0.93984 -1.11296
H -4.03616  0.02718 -2.48745
H -5.90865 -0.77228 -0.94339
H -5.96462  0.91276 -1.43339
H -6.50497  0.62721  0.96512
H -4.83254 -1.29346  1.40953
H -3.55441 -0.07622  1.44734
```

(*E*)-**4a-TS@1**$_2$

```
C -9.01347 -3.03417 -1.98033
C -8.40152 -1.82769 -2.69075
C -8.42638 -3.20310 -0.58095
H -8.70432 -3.91201 -2.54868
C -8.92177 -0.53667 -2.56777
C -7.26660 -1.97552 -3.49885
C -8.93314 -2.51450  0.52941
C -7.34267 -4.06156 -0.35781
C -8.36969  0.57684 -3.21313
H -9.78852 -0.38711 -1.93518
C -6.70707 -0.91483 -4.20036
O -6.71102 -3.25243 -3.61895
C -8.39763 -2.64061  1.81513
H -9.78686 -1.86235  0.38613
C -6.74988 -4.19652  0.89303
O -6.84322 -4.82795 -1.42387
C -8.93914  1.97832 -3.02379
C -7.26479  0.35073 -4.04473
H -5.84403 -1.06431 -4.83640
C -5.55473 -3.51409 -2.95102
C -8.99729 -1.95433  3.04520
C -7.27756 -3.47032  1.95167
H -5.89576 -4.84552  1.03543
C -5.61391 -4.43505 -1.86668
H -8.60450  2.56129 -3.88258
C -8.35065  2.66621 -1.79162
O -6.71405  1.42447 -4.76385
C -8.41502 -0.55844  3.24340
H -8.67725 -2.54396  3.90498
O -6.71585 -3.59795  3.22451
C -8.89948  2.54064 -0.51211
C -7.23238  3.50036 -1.91868
C -5.50235  1.86011 -4.30866
C -3.34560 -3.36150 -2.66371
C -8.94677  0.55948  2.58836
C -7.30621 -0.34294  4.07300
C -5.54247 -2.95480  3.46084
C -3.38812 -4.35235 -1.69243
C -8.41112  3.23837  0.60066
H -9.74635  1.87847 -0.37407
C -6.72893  4.24060 -0.85952
O -6.64254  3.61806 -3.18098
C -5.47827  2.94924 -3.39155
C -8.39668  1.83917  2.69153
H -9.82178  0.42236  1.96448
C -6.71427  0.90986  4.20137
O -6.77651 -1.41450  4.81038
C -5.56239 -1.86401  4.37549
C -9.00219  3.05698  1.99512
C -7.33925  4.11207  0.38393
H -5.87900  4.89655 -0.99506
C -3.28406  1.67540 -4.11688
C -7.24688  1.97020  3.48167
H -5.84390  1.04707  4.82996
C -3.33867 -2.70159  3.24294
H -8.68981  3.92651  2.57473
O -6.85424  4.88920  1.44981
C -3.28075  2.65073 -3.12734
O -6.66503  3.23713  3.58517
C -3.33942 -1.71145  4.21625
C -5.61449  4.49469  1.86457
C -5.53017  3.50514  2.88589
C -3.39695  4.42335  1.62880
C -3.33521  3.34811  2.50427
C -10.53794  3.03266  1.98841
H -10.91749  2.94897  3.01024
H -10.95015  2.19825  1.41400
C -10.47547  1.99897 -3.02282
H -10.83522  3.02895 -2.95456
H -10.91004  1.44323 -2.18723
C -10.54914 -3.00031 -1.97927
H -10.92423 -2.93546 -3.00411
H -10.95787 -2.15104 -1.42451
C -10.53325 -1.96209  3.02568
H -10.90123 -2.98865  2.94956
H -10.95344 -1.39981  2.18704
H -10.92197 -1.51980  3.94679
H -10.94229 -3.91172 -1.52127
H -10.85673  1.55679 -3.94711
H -10.92322  3.95607  1.54803
C  9.01715 -0.81762 -3.53849
C  8.44092 -1.94792 -2.68826
C  8.43099  0.54157 -3.14012
H  8.67901 -1.00235 -4.55915
C  8.98391 -2.28457 -1.43865
C  7.31673 -2.67191 -3.10785
C  9.00067  1.37126 -2.16817
C  7.27731  1.02130 -3.77362
C  8.42672 -3.25089 -0.59636
H  9.86615 -1.75277 -1.10234
C  6.71391 -3.62972 -2.30057
O  6.76377 -2.44808 -4.37974
C  8.48549  2.63518 -1.85034
H  9.87849  1.02146 -1.63752
C  6.75164  2.28090 -3.53139
O  6.66759  0.20298 -4.72481
C  9.01678 -3.62786  0.76630
C  7.26155 -3.88097 -1.05285
H  5.82953 -4.15592 -2.63494
C  5.54099 -1.83923 -4.33924
C  9.06512  3.50794 -0.74208
C  7.37581  3.07549 -2.58036
H  5.87538  2.63007 -4.06089
C  5.51085 -0.41762 -4.38008
H  8.68998 -4.64984  0.96403
C  8.42729 -2.76850  1.88446
O  6.66700 -4.83081 -0.22411
C  8.42141  3.16469  0.60111
H  8.76845  4.53255 -0.96946
O  6.89474  4.37344 -2.36025
C  8.94850 -1.50255  2.19387
C  7.31750 -3.19656  2.62658
C  5.51004 -4.50506  0.40904
C  3.32800 -1.85565 -4.05978
C  8.91874  2.14905  1.42228
C  7.30067  3.86413  1.07041
C  5.66198  4.44961 -1.78811
C  3.32761 -0.47174 -3.94403
C  8.38691 -0.65912  3.15645
H  9.81903 -1.15792  1.64814
C  6.71064 -2.38696  3.58088
O  6.78250 -4.48032  2.42699
C  5.55246 -4.46406  1.82961
C  8.37622  1.82997  2.67289
H  9.77176  1.57982  1.07309
C  6.74096  3.61098  2.31627
O  6.76523  4.88447  0.27768
C  5.59555  4.64863 -0.37909
C  8.96146  0.70880  3.52950
C  7.24169 -1.12866  3.81091
H  5.83644 -2.72934  4.11873
C  3.31729 -4.11165  0.48622
C  7.28677  2.60009  3.10264
H  5.88367  4.17432  2.66246
C  3.43369  4.36091 -1.87361
H  8.63055  0.90512  4.55016
O  6.65339 -0.29257  4.76140
C  3.33407 -4.22367  1.87005
O  6.72193  2.37006  4.36734
C  3.37267  4.48974 -0.49329
C  5.49006  0.32116  4.42627
C  5.50927  1.74065  4.32929
C  3.30099  0.34094  4.01800
C  3.29528  1.72880  4.04937
C 10.49768  0.71557  3.53477
H 10.86739  1.68979  3.86539
H 10.93012  0.51616  2.55023
C 10.55217 -3.62493  0.76299
H 10.92906 -3.96176  1.73229
H 10.97758 -2.63584  0.57070
C 10.55257 -0.81015 -3.55483
H 10.92773 -1.77389 -3.90930
H 10.99016 -0.62954 -2.56890
C 10.59919  3.47132 -0.68802
H 11.01360  3.78445 -1.65002
H 10.99711  2.47689 -0.46662
H 10.96451  4.15095  0.08646
H 10.91715 -0.02654 -4.22426
H 10.92752 -4.29995 -0.01075
H 10.87244 -0.05026  4.21903
C -2.00012  4.74722  1.17374
C -1.92071  2.83634  2.52092
C -1.97890 -4.62308 -1.23149
C -1.93166 -2.87066 -2.78013
C  1.90763 -3.82277  0.05166
C  1.91382 -4.12504  2.36457
C  1.91221 -0.13704  3.71267
C  1.88206  2.19749  3.83316
C  1.91107 -2.34094 -3.92538
C  1.93308 -0.02354 -3.61071
C  2.02947  4.22404 -2.40270
C -1.86808  1.20878 -4.32249
C -1.88089  2.77608 -2.58904
C  1.93746  4.36292 -0.07122
C -1.92436 -1.21882  4.38194
C -1.94885 -2.82542  2.69035
N -1.18561 -1.95077  3.43566
N -1.20147  3.75333  1.77394
N  1.14620  1.01555  3.63998
N  1.15440 -3.89806  1.19998
N -1.19959 -3.67190 -1.92301
N -1.12406  1.92241 -3.36584
N  1.16786 -1.17388 -3.64781
N  1.22073  4.24559 -1.24811
O -1.51371  1.80371  3.02596
O -1.60490  5.64511  0.47179
O -1.49715  3.47399 -1.66474
O  1.63505  4.11730 -3.53777
O  1.50159  4.30844  1.06605
O  1.43410  3.31965  3.80742
O  1.54418 -1.28498  3.54798
O  1.47543 -4.23259  3.48322
O -1.48024 -0.38028  5.12952
O -1.53217 -1.92805 -3.44126
O -1.56282 -5.45500 -0.46440
O -1.59058 -3.52005  1.75325
O  1.45932 -3.45404 -4.03608
O  1.52187 -3.56248 -1.07911
O  1.56515  1.10681 -3.33212
O -1.42048  0.42115 -5.12007
H -0.18150  3.79097  1.68246
```

```
H  0.20545  4.10107 -1.29583
H -0.11304  1.76750 -3.27108
H  0.14538 -1.22498 -3.56398
H -0.18757 -3.59217 -1.76070
H  0.14241 -3.72230  1.25118
H -0.17502 -1.80022  3.31652
H  0.13485  1.05474  3.49090
N -4.40025  1.24270 -4.70772
N -4.37354  3.32462 -2.76140
N -4.39459  2.89056  3.17837
N -4.54683  4.99656  1.26549
N -4.45830 -1.25703  4.78618
N -4.43461 -3.35722  2.85716
N -4.42321 -2.93900 -3.33002
N -4.52992 -4.88987 -1.25576
N  4.40110 -4.27605 -0.27744
N  4.46152 -4.36832  2.57334
N  4.44501 -2.57182 -4.22260
N  4.41512  0.28084 -4.12731
N  4.39361 -0.39383  4.22604
N  4.40718  2.45839  4.17298
N  4.58445  4.32580 -2.54861
N  4.44872  4.64620  0.28297
N -3.30978 -0.42091 -1.10608
C -2.25202 -0.71920 -0.58876
O -1.20484  0.26509 -0.51151
C -0.08763 -0.04091  0.19543
O  0.06985 -1.07441  0.81056
C  0.90298  1.09553  0.12125
H -1.91996 -1.65666 -0.13725
H  0.68674  1.75297  0.96868
H  0.68942  1.67409 -0.78047
C  2.34597  0.66337  0.14628
C  2.76919 -0.48581 -0.53392
C  3.30635  1.45547  0.77770
C  4.11897 -0.81215 -0.60469
H  2.04085 -1.13737 -1.00094
C  4.65977  1.12633  0.70514
H  3.00425  2.34546  1.31779
C  5.08722 -0.00513  0.00859
H  4.42595 -1.70615 -1.14073
H  5.39048  1.76166  1.19160
C  6.53937 -0.37017 -0.06689
H  6.75725 -1.23065  0.56956
H  6.82330 -0.64197 -1.08379
H  7.18103  0.45136  0.25474
C -5.71109 -0.61619 -0.59700
C -5.79453  0.18696  0.70479
C -4.53227  0.12237  1.56645
C -4.61380 -0.13321 -1.55374
H -6.03324  1.23196  0.46561
H -4.71362  0.49302  2.57323
H -4.75326  0.94623 -1.71037
H -4.73085 -0.61308 -2.52921
H -5.55170 -1.67739 -0.37135
H -6.66518 -0.53407 -1.12266
H -6.64410 -0.19223  1.27794
H -4.17934 -0.90905  1.63907
H -3.71633  0.71282  1.14672
```

(*E*)-**4a**@**1**$_2$

```
C -8.98307 -2.95922 -2.11408
C -8.37574 -1.70485 -2.74002
C -8.39166 -3.21929 -0.73162
H -8.67164 -3.79470 -2.74171
C -8.90735 -0.42929 -2.52914
C -7.24877 -1.79298 -3.56818
C -8.90003 -2.61403  0.42510
C -7.30156 -4.08431 -0.57334
C -8.37874  0.72637 -3.11343
H -9.77039 -0.33040 -1.88145
C -6.70268 -0.68133 -4.20004
O -6.70048 -3.06192 -3.79706
C -8.35854 -2.82755  1.69651
H -9.75845 -1.95909  0.33042
C -6.70319 -4.30403  0.66283
O -6.81313 -4.77360 -1.69559
C -8.96617  2.10803 -2.84358
C -7.27451  0.56524 -3.96059
H -5.84273 -0.77791 -4.85043
C -5.53760 -3.38500 -3.16945
C -8.95194 -2.22494  2.97088
C -7.23372 -3.65850  1.77190
H -5.84502 -4.95646  0.75598
C -5.58946 -4.35998 -2.13138
H -8.64279  2.74179 -3.66986
C -8.38541  2.73876 -1.57747
O -6.74362  1.69225 -4.60519
C -8.37263 -0.84168  3.24794
H -8.62278 -2.86450  3.79035
O -6.66557 -3.86460  3.03391
C -8.91465  2.51338 -0.30374
C -7.28870  3.60732 -1.66085
C -5.54024  2.12862 -4.12532
C -3.32457 -3.25544 -2.89112
C -8.90324  0.30828  2.64938
C -7.27541 -0.66912  4.10103
C -5.50187 -3.21403  3.29646
C -3.36182 -4.29177 -1.96845
C -8.42587  3.14136  0.84963
H -9.74276  1.82129 -0.20178
C -6.78949  4.28424 -0.55721
O -6.70926  3.81619 -2.91520
C -5.53030  3.18212 -3.17168
C -8.36729  1.58410  2.83702
H -9.77405  0.20152  2.01351
C -6.68806  0.57634  4.30136
O -6.73497 -1.78379  4.76812
C -5.52277 -2.18762  4.28379
C -8.99152  2.84339  2.23453
C -7.37680  4.05237  0.68232
H -5.95255  4.96307 -0.65668
C -3.31686  1.99106 -3.96929
C -7.21983  1.67176  3.63727
H -5.81547  0.67938  4.93310
C -3.31851 -2.86599  3.01475
H -8.68156  3.67059  2.87440
O -6.88082  4.74991  1.80012
C -3.32150  2.94288 -2.95588
O -6.63206  2.92971  3.79864
C -3.31235 -1.94754  4.05519
C -5.63307  4.32611  2.15858
C -5.52376  3.24013  3.07573
C -3.42325  4.27587  1.86046
C -3.34735  3.09948  2.59428
C -10.52711  2.80073  2.24723
H -10.89084  2.63330  3.26457
H -10.93819  2.00851  1.61539
C -10.50278  2.10639 -2.83681
H -10.87697  3.12581 -2.71089
H -10.92646  1.49972 -2.03156
C -10.51879 -2.93301 -2.10895
H -10.89473 -2.79747 -3.12654
H -10.93223 -2.12753 -1.49568
C -10.48805 -2.23769  2.96373
H -10.85291 -3.25880  2.82453
H -10.91821 -1.62406  2.16735
H -10.86995 -1.85780  3.91506
H -10.90698 -3.87684 -1.71697
H -10.88150  1.71011 -3.78273
H -10.92895  3.75080  1.88532
C  8.99501 -0.58105 -3.55930
C  8.41950 -1.76983 -2.79097
C  8.41890  0.75123 -3.06542
H  8.65206 -0.69182 -4.58905
C  8.97238 -2.20622 -1.57733
C  7.28925 -2.45555 -3.25704
C  8.99426  1.50607 -2.03687
C  7.27104  1.28591 -3.66483
C  8.42213 -3.23779 -0.81025
H  9.85582 -1.70059 -1.20574
C  6.69610 -3.47891 -2.52704
O  6.72481 -2.12720 -4.50052
C  8.49135  2.75047 -1.63388
H  9.86467  1.11053 -1.52621
C  6.76021  2.53266 -3.33946
O  6.64794  0.54034 -4.66496
C  9.01746 -3.70976  0.52103
C  7.25601 -3.83320 -1.30947
H  5.80976 -3.97797 -2.89606
C  5.50726 -1.51383 -4.40869
C  9.07031  3.53486 -0.46158
C  7.39077  3.25377 -2.33585
H  5.88914  2.92611 -3.84602
C  5.48817 -0.09261 -4.35309
H  8.69093 -4.74280  0.64912
C  8.42982 -2.92768  1.69691
O  6.66635 -4.84883 -0.55723
C  8.42092  3.09179  0.84923
H  8.77723  4.57468 -0.61110
O  6.91899  4.53754 -2.02567
C  8.95259 -1.68772  2.09712
C  7.31331 -3.40202  2.39968
C  5.50735 -4.57159  0.09595
C  3.29382 -1.52988 -4.12964
C  8.91735  2.01957  1.59523
C  7.29746  3.75321  1.36488
C  5.68014  4.57402 -1.46271
C  3.30519 -0.15774 -3.91451
C  8.38208 -0.90885  3.10836
H  9.82950 -1.31064  1.58385
C  6.69513 -2.65462  3.39614
O  6.77992 -4.66885  2.10928
C  5.54944 -4.61086  1.51640
C  8.37644  1.61263  2.82119
H  9.76713  1.47357  1.20359
C  6.74326  3.41673  2.59291
O  6.75902  4.82412  0.64454
C  5.59733  4.63991 -0.04141
C  8.95745  0.42593  3.58864
C  7.22475 -1.41521  3.71199
H  5.81354 -3.02855  3.89957
C  3.31703 -4.17193  0.19717
C  7.29215  2.35558  3.30859
H  5.88816  3.95633  2.98009
C  3.45291  4.50213 -1.58272
H  8.62366  0.54239  4.62055
O  6.62564 -0.64000  4.70533
C  3.33350 -4.34886  1.57381
O  6.72715  2.04266  4.55626
C  3.37562  4.49097 -0.19758
C  5.47554  0.01111  4.40130
C  5.50722  1.43271  4.46066
C  3.29832  0.09947  3.94605
C  3.29756  1.47216  4.15506
C 10.49358  0.42993  3.59840
H 10.86366  1.37207  4.01132
H 10.92792  0.31431  2.60155
C 10.55265 -3.70589  0.51267
H 10.93320 -4.11282  1.45324
H 10.97688 -2.70517  0.39210
C 10.53056 -0.57766 -3.58206
H 10.90074 -1.51461 -4.00661
H 10.97259 -0.46976 -2.58765
C 10.60408  3.48896 -0.40561
H 11.02264  3.87787 -1.33768
H 10.99790  2.47845 -0.26489
H 10.96902  4.10210  0.42265
H 10.89508  0.25083 -4.19508
H 10.92555 -4.32206 -0.30980
H 10.86577 -0.39156  4.21628
C -2.03579  4.65766  1.42723
C -1.93564  2.58674  2.50917
```

C -1.95161 -4.56555 -1.50878
C -1.92589 -2.70826 -2.92726
C 1.91392 -3.83886 -0.22062
C 1.91523 -4.24430 2.07597
C 1.91782 -0.32065 3.53859
C 1.89028 1.97538 3.98061
C 1.87146 -2.00968 -4.04111
C 1.91266 0.27809 -3.55172
C 2.05529 4.42638 -2.14053
C -1.89900 1.54975 -4.19809
C -1.91665 3.08571 -2.43793
C 1.93480 4.32531 0.19036
C -1.90668 -1.42493 4.20884
C -1.94733 -2.91157 2.40986
N -1.18417 -2.05871 3.18128
N -1.22989 3.58780 1.87000
N 1.15405 0.83101 3.61932
N 1.15985 -3.94579 0.92467
N -1.18767 -3.54871 -2.11570
N -1.15282 2.27105 -3.25032
N 1.13812 -0.85986 -3.67522
N 1.23048 4.33253 -0.99888
O -1.51328 1.49985 2.87371
O -1.64595 5.64560 0.85667
O -1.52525 3.76392 -1.50156
O 1.67563 4.43742 -3.28492
O 1.48665 4.15072 1.31225
O 1.44414 3.09136 4.09698
O 1.54965 -1.42772 3.18770
O 1.47643 -4.39414 3.18951
O -1.45523 -0.63843 5.00683
O -1.55029 -1.69158 -3.48496
O -1.52712 -5.44280 -0.79870
O -1.59613 -3.54022 1.42510
O 1.40932 -3.10467 -4.24582
O 1.53407 -3.52197 -1.33849
O 1.55116 1.38749 -3.19109
O -1.44728 0.76842 -5.00105
H -0.21044 3.63869 1.76494
H 0.21001 4.22187 -1.07607
H -0.14483 2.11561 -3.15448
H 0.11596 -0.90800 -3.59899
H -0.17942 -3.45583 -1.93311
H 0.14885 -3.76241 0.97465
H -0.17779 -1.89112 3.04705
H 0.14793 0.88828 3.43725
N -4.42810 1.54571 -4.55330
N -4.42319 3.57207 -2.55132
N -4.38931 2.57970 3.25163
N -4.57832 4.88912 1.59361
N -4.42297 -1.57447 4.69669
N -4.40527 -3.53395 2.62743
N -4.40418 -2.80561 -3.53505
N -4.50162 -4.85168 -1.55592
N 4.39835 -4.30313 -0.57567
N 4.46009 -4.54249 2.26636
N 4.40468 -2.24290 -4.34205
N 4.39952 0.59598 -4.04726
N 4.38108 -0.66557 4.08979
N 4.41271 2.17303 4.37852
N 4.61185 4.52811 -2.24417
N 4.44279 4.57022 0.60352
N -3.30765 0.33325 -1.33103
C -2.32677 -0.19556 -0.75116
O -1.19301 0.56669 -0.60882
C -0.09690 0.01203 0.00407
O -0.04998 -1.13555 0.37516
C 0.95570 1.07517 0.13551
H -2.25155 -1.20208 -0.33375
H 0.70773 1.63374 1.04296
H 0.81651 1.76999 -0.69657
C 2.38212 0.60057 0.17927
C 2.81338 -0.51334 -0.55128
C 3.33164 1.35812 0.86807
C 4.16503 -0.83490 -0.61569
H 2.09349 -1.13626 -1.06633
C 4.68476 1.03216 0.80210
H 3.02019 2.22207 1.44355
C 5.12285 -0.05926 0.05144
H 4.48207 -1.69781 -1.19469
H 5.40794 1.64067 1.33201
C 6.57770 -0.40289 -0.04381
H 6.77233 -1.39132 0.37442
H 6.90658 -0.42293 -1.08377
H 7.19951 0.31557 0.49065
C -5.70492 0.30017 -0.83146
C -5.40498 0.84382 0.56452
C -4.94283 -0.23564 1.53712
C -4.52273 -0.45311 -1.44525
H -4.63777 1.62158 0.47772
H -4.75246 0.18115 2.52520
H -4.70433 -0.62084 -2.50762
H -4.42150 -1.44342 -0.97174
H -6.56489 -0.37784 -0.80125
H -5.98365 1.12844 -1.48453
H -6.30546 1.32659 0.95247
H -5.70458 -1.01558 1.62357
H -4.01884 -0.71135 1.20173

5a-TS@1$_2$

C 8.93280 -2.86992 2.23039
C 8.31373 -1.58749 2.78208
C 8.34903 -3.21131 0.86318
H 8.62650 -3.67141 2.90313
C 8.84059 -0.31653 2.53993
C 7.17053 -1.65742 3.58717
C 8.85095 -2.63120 -0.30694
C 7.28654 -4.11435 0.72289
C 8.29359 0.84949 3.08894
H 9.72122 -0.22962 1.91450
C 6.58943 -0.53503 4.16447
O 6.65960 -2.92837 3.85385
C 8.33933 -2.90522 -1.57711
H 9.67783 -1.93556 -0.22317
C 6.73216 -4.41204 -0.51810
O 6.77016 -4.75718 1.85952
C 8.88899 2.22672 2.82165
C 7.17051 0.70356 3.91216
H 5.71629 -0.62259 4.79797
C 5.49487 -3.29163 3.25818
C 8.93139 -2.29184 -2.84452
C 7.25293 -3.78628 -1.64442
H 5.89507 -5.09357 -0.60013
C 5.54406 -4.31505 2.26899
H 8.55567 2.86819 3.63779
C 8.32127 2.83486 1.54193
O 6.64605 1.84366 4.54547
C 8.34347 -0.90958 -3.10687
H 8.60599 -2.92518 -3.67011
O 6.69297 -4.05216 -2.89965
C 8.85867 2.56958 0.28129
C 7.22770 3.70760 1.59069
C 5.45245 2.28764 4.06764
C 3.27885 -3.22254 3.04734
C 8.89556 0.25654 -2.56395
C 7.20905 -0.76509 -3.91479
C 5.50272 -3.44622 -3.17127
C 3.31203 -4.28126 2.15109
C 8.38527 3.16435 -0.89382
H 9.68250 1.86930 0.20785
C 6.73648 4.35114 0.46313
O 6.64067 3.95099 2.83835
C 5.45836 3.33658 3.10022
C 8.35543 1.52495 -2.79028
H 9.78947 0.17202 -1.95733
C 6.60980 0.46970 -4.14386
O 6.69583 -1.90262 -4.55076
C 5.49848 -2.35530 -4.08451
C 8.98033 2.81127 -2.25253
C 7.33171 4.07876 -0.76606
H 5.90299 5.03829 0.53313
C 3.22641 2.18397 3.91390
C 7.19065 1.58989 -3.56480
H 5.72326 0.55195 -4.75899
C 3.28735 -3.26095 -2.97997
H 8.68275 3.60716 -2.93606
O 6.85549 4.75724 -1.90013
C 3.24630 3.12366 2.89345
O 6.66964 2.86118 -3.81364
C 3.27452 -2.24910 -3.93092
C 5.61366 4.35062 -2.29925
C 5.52500 3.25383 -3.20618
C 3.38563 4.39265 -2.16167
C 3.31394 3.24222 -2.93363
C 10.51525 2.76818 -2.23540
H 10.89530 2.55442 -3.23799
H 10.91623 2.00599 -1.56162
C 10.42539 2.22612 2.83455
H 10.80015 3.24492 2.70544
H 10.86066 1.61349 2.04026
C 10.46837 -2.83259 2.22671
H 10.84073 -2.63965 3.23644
H 10.87778 -2.05887 1.57133
C 10.46741 -2.29921 -2.83364
H 10.83461 -3.32118 -2.70811
H 10.89376 -1.69689 -2.02656
H 10.85115 -1.90468 -3.77835
H 10.86443 -3.79333 1.88745
H 10.79153 1.83823 3.78883
H 10.91211 3.73389 -1.91145
C -8.96137 -0.38639 3.58393
C -8.39122 -1.59619 2.84941
C -8.37420 0.92009 3.04717
H -8.62052 -0.46933 4.61667
C -8.93665 -2.04844 1.64015
C -7.28817 -2.29836 3.35270
C -8.94170 1.64014 1.99132
C -7.22331 1.46257 3.63138
C -8.41568 -3.12972 0.92537
H -9.80216 -1.53197 1.24211
C -6.71195 -3.36078 2.66361
O -6.73555 -1.93876 4.59282
C -8.42804 2.86489 1.54716
H -9.81781 1.23694 1.49684
C -6.69221 2.68705 3.25524
O -6.62238 0.74692 4.66733
C -9.03157 -3.64848 -0.37438
C -7.27247 -3.74188 1.45422
H -5.84095 -3.86761 3.05784
C -5.51253 -1.33832 4.48365
C -9.00887 3.61473 0.35377
C -7.31482 3.37587 2.22365
H -5.81325 3.08698 3.74269
C -5.47408 0.07961 4.38436
H -8.73218 -4.69390 -0.45834
C -8.42888 -2.93053 -1.57762
O -6.71987 -4.80687 0.74207
C -8.35806 3.11899 -0.93587
H -8.71250 4.65788 0.46747
O -6.83197 4.63994 1.85911
C -8.90763 -1.68484 -2.00669
C -7.34714 -3.47586 -2.28108
C -5.56454 -4.59886 0.05922
C -3.30345 -1.39645 4.18691
C -8.84452 2.00872 -1.63102
C -7.23776 3.76372 -1.47644
C -5.60653 4.66474 1.26986
C -3.29475 -0.03022 3.94075
C -8.33424 -0.97172 -3.06241
H -9.75782 -1.25482 -1.48976
C -6.72259 -2.79218 -3.31872
O -6.85857 -4.74892 -1.94267
C -5.62325 -4.70909 -1.35841

```
C -8.29162  1.55070 -2.83184
H -9.70039  1.48228 -1.22571
C -6.64981  3.35368 -2.66691
O -6.73479  4.89236 -0.81921
C -5.55672  4.76286 -0.15043
C -8.88218  0.35795 -3.57807
C -7.21276 -1.54518 -3.67213
H -5.86426 -3.21646 -3.82257
C -3.36149 -4.29518 -0.08337
C -7.18908  2.25639 -3.33184
H -5.79110  3.87594 -3.06962
C -3.37674  4.61321  1.33517
H -8.53775  0.44674 -4.60906
O -6.59377 -0.82496 -4.69626
C -3.39946 -4.54104 -1.44805
O -6.61573  1.87272 -4.55502
C -3.33068  4.68370 -0.05004
C -5.43017 -0.19774 -4.38462
C -5.42024  1.22451 -4.42654
C -3.25663 -0.17854 -3.90250
C -3.21700  1.19649 -4.07930
C -10.41843  0.38351 -3.60288
H -10.77075  1.32264 -4.03771
H -10.86381  0.29312 -2.60838
C -10.56641 -3.60458 -0.36082
H -10.96098 -4.04417 -1.28072
H -10.96431 -2.58898 -0.28486
C -10.49712 -0.37478  3.60413
H -10.87227 -1.29656  4.05666
H -10.93775 -0.29519  2.60652
C -10.54259  3.57124  0.30003
H -10.96038  3.98675  1.22097
H -10.93955  2.55869  0.18628
H -10.90544  4.16283 -0.54469
H -10.85801  0.47370  4.19157
H -10.95109 -4.17331  0.48995
H -10.79610 -0.44456 -4.20857
C  1.98572  4.78513 -1.76879
C  1.89000  2.76799 -2.95461
C  1.88946 -4.59090  1.75115
C  1.86400 -2.75111  3.19138
C -1.94295 -4.00187  0.31044
C -1.98454 -4.52355 -1.96520
C -1.88677 -0.65018 -3.51768
C -1.79805  1.65325 -3.88487
C -1.89197 -1.90166  4.08210
C -1.89901  0.37651  3.56282
C -1.96527  4.51623  1.85096
C  1.79679  1.77641  4.15015
C  1.84999  3.28170  2.36418
C -1.89768  4.59386 -0.48509
C  1.84554 -1.82693 -4.14691
C  1.88249 -3.45748 -2.48682
N  1.10205 -2.66255 -3.30403
N  1.17465  3.76827 -2.30787
N -1.08875  0.48033 -3.58009
N -1.20433 -4.18813 -0.83966
N  1.11868 -3.61990  2.41905
N  1.06396  2.49625  3.18592
N -1.14252 -0.76935  3.70983
N -1.17003  4.51154  0.69007
O  1.48110  1.70208 -3.36935
O  1.60402  5.73647 -1.13075
O  1.50524  3.92193  1.38479
O -1.55650  4.45075  2.98563
O -1.47223  4.54377 -1.62469
O -1.34302  2.77229 -3.94488
O -1.56276 -1.78022 -3.20203
O -1.56496 -4.75471 -3.07243
O  1.39885 -0.96382 -4.86713
O  1.48894 -1.77852  3.82179
O  1.46257 -5.46435  1.03636
O  1.51984 -4.13654 -1.54285
O -1.45680 -3.01189  4.27148
O -1.54863 -3.64823  1.40940
O -1.53280  1.47797  3.18752
O  1.33773  1.00455  4.95599
H  0.15426  3.83975 -2.26612
H -0.15079  4.42809  0.74859
H  0.05030  2.35276  3.10233
H -0.12893 -0.84564  3.58890
H  0.10966 -3.54779  2.26014
H -0.17857 -4.13959 -0.90870
H  0.10472 -2.47700 -3.14870
H -0.07935  0.49884 -3.44251
N  4.33413  1.72404  4.50093
N  4.35908  3.72942  2.47361
N  4.37859  2.65330 -3.48338
N  4.54556  4.94865 -1.79658
N  4.38402 -1.76202 -4.48831
N  4.39744 -3.88826 -2.58938
N  4.36440 -2.71069  3.62835
N  4.45169 -4.83571  1.72759
N -4.43880 -4.33728  0.70471
N -4.54056 -4.72075 -2.12026
N -4.42165 -2.08623  4.42746
N -4.37552  0.74310  4.05781
N -4.35859 -0.90920 -4.06848
N -4.30687  1.93259 -4.30733
N -4.51918  4.59675  2.02447
N -4.41610  4.75859 -0.82338
C -1.81726  0.67614 -0.18440
C -2.19995 -0.48767  0.49453
C -2.81423  1.43712 -0.80362
C -3.54437 -0.84992  0.57987
H -1.45080 -1.11075  0.96673
C -4.15868  1.07326 -0.71384
H -2.54422  2.31987 -1.37056
C -4.54977 -0.07308 -0.01482
H -3.81603 -1.74728  1.12854
H -4.91017  1.69184 -1.19670
C -5.99978 -0.47663  0.06688
H -6.25405 -1.19312 -0.72221
H -6.22875 -0.95333  1.02260
H -6.66387  0.38441 -0.04989
C  4.85980  0.94429  0.49619
C  5.44618 -0.31850 -0.14844
C  4.55903 -1.55856 -0.02621
C  3.53771  1.43595 -0.12206
H  5.65057 -0.11248 -1.20780
H  5.05941 -2.44219 -0.42013
H  3.60784  1.32802 -1.20969
H  3.40813  2.50523  0.08247
H  4.74504  0.79125  1.57675
H  5.58137  1.76024  0.38651
H  6.41990 -0.52078  0.31380
H  4.32191 -1.75908  1.02305
H  3.61241 -1.44293 -0.56010
C -0.37983  1.16982 -0.19491
H -0.22961  1.86567 -1.02363
H -0.15290  1.70217  0.72854
N  2.32448  0.73958  0.27480
C  2.06761  0.03251  1.32137
H  2.70067 -0.10964  2.19922
C  0.62683  0.06620 -0.38551
O  0.72603 -0.73974 -1.25225
O  0.92208 -0.56902  1.26931
```

5a@1$_2$

```
C -8.94983 -3.11462  2.02030
C -8.37373 -3.27550  0.61450
C -8.32906 -1.91250  2.72252
H -8.63711 -3.99534  2.58213
C -8.90210 -2.62017 -0.49988
C -7.26865 -4.10646  0.39258
C -8.83554 -0.61705  2.55465
C -7.21511 -2.06127  3.55727
C -8.40334 -2.78787 -1.79777
H -9.73443 -1.94184 -0.35372
C -6.75646 -4.34037 -0.87493
O -6.70047 -4.74771  1.50030
C -8.26229  0.50564  3.15560
H -9.71825 -0.48392  1.94068
C -6.58866 -0.97321  4.15635
O -6.71856 -3.35166  3.81467
C -8.97770 -2.00756 -2.97787
C -7.33922 -3.68477 -1.95627
H -5.91579 -5.00618 -1.02167
C -5.51583 -4.27588  1.96505
C -8.84800  1.91277  3.04665
C -7.11122  0.28964  3.92246
H -5.70954 -1.10908  4.77213
C -5.51643 -3.62368  3.23371
H -8.66553 -2.53915 -3.87768
C -8.35440 -0.61282 -3.06537
O -6.82963 -3.95015 -3.23911
C -8.26853  2.68232  1.86404
H -8.52559  2.44442  3.94284
O -6.52010  1.40999  4.51111
C -8.91399  0.51888 -2.46351
C -7.17052 -0.42352 -3.78804
C -5.58440 -3.42759 -3.44431
C -3.30351 -4.00375  1.85686
C -8.77439  2.51985  0.57178
C -7.22279  3.59996  2.01900
C -5.40098  1.92074  3.94360
C -3.29047 -3.45848  3.13266
C -8.34851  1.79233 -2.58503
H -9.82867  0.40825 -1.89317
C -6.57182  0.81878 -3.95311
O -6.63211 -1.54455 -4.42270
C -5.49242 -2.09736 -3.94762
C -8.29475  3.22075 -0.53776
H -9.58699  1.81861  0.42233
C -6.68091  4.29433  0.94240
O -6.69249  3.83729  3.30401
C -5.46614  3.26357  3.46509
C -8.92517  3.03313 -1.91443
C -7.18157  1.90955 -3.34748
H -5.66521  0.93291 -4.53216
C -3.36168 -3.49022 -3.26255
C -7.21798  4.09239 -0.32380
H -5.85432  4.97905  1.08620
C -3.23579  1.87164  3.42756
H -8.61269  3.88682 -2.51619
O -6.68232  3.19793 -3.54479
C -3.28823 -2.15449 -3.63333
O -6.69750  4.82959 -1.39809
C -3.26234  3.22759  3.13592
C -5.48473  3.54073 -3.00874
C -5.49026  4.43341 -1.89609
C -3.25913  3.54509 -2.98680
C -3.25887  4.44166 -1.92726
C -10.45991  3.03916 -1.86531
H -10.81611  3.97496 -1.42667
H -10.87354  2.22046 -1.26980
C -10.51297 -1.97444 -2.97064
H -10.88187 -1.44931 -3.85582
H -10.92603 -1.47049 -2.09232
C -10.48551 -3.08385  2.02678
H -10.88003 -3.99403  1.56737
H -10.89900 -2.23326  1.47787
C -10.38475  1.90986  3.04199
H -10.76338  1.39984  3.93200
H -10.80773  1.40749  2.16756
H -10.75877  2.93696  3.04285
H -10.85423 -3.02394  3.05425
H -10.90858 -2.99350 -2.98021
H -10.86749  2.95118 -2.87618
C  9.01014 -3.65498 -0.73931
C  8.41928 -3.34535  0.63223
C  8.37458 -2.75378 -1.79733
H  8.70962 -4.67479 -0.98253
```

C 8.91585 -2.31444 1.44106
C 7.32116 -4.06592 1.11749
C 8.89024 -1.49679 -2.12372
C 7.22799 -3.16549 -2.48903
C 8.34149 -1.96751 2.66718
H 9.78196 -1.76111 1.09749
C 6.69497 -3.74257 2.31396
O 6.83258 -5.15660 0.38219
C 8.33159 -0.67185 -3.10702
H 9.77196 -1.14766 -1.60011
C 6.63578 -2.39171 -3.47891
O 6.71137 -4.43181 -2.20123
C 8.90576 -0.88121 3.58408
C 7.20218 -2.68415 3.05028
H 5.82368 -4.28845 2.64989
C 5.60003 -4.96254 -0.16661
C 8.93595 0.68437 -3.45597
C 7.20071 -1.15577 -3.77718
H 5.75908 -2.74136 -4.00879
C 5.54195 -4.50491 -1.51394
H 8.54618 -1.11488 4.58702
C 8.33995 0.49328 3.23922
O 6.57444 -2.31698 4.24277
C 8.39714 1.79655 -2.55853
H 8.59273 0.91712 -4.46467
O 6.64080 -0.39814 -4.81677
C 8.92216 1.30051 2.25276
C 7.21369 1.00542 3.89661
C 5.42177 -1.60942 4.13253
C 3.37193 -4.88555 -0.05663
C 8.96004 2.12126 -1.32211
C 7.29925 2.56159 -2.96962
C 5.45783 0.21223 -4.51622
C 3.32545 -4.37409 -1.34512
C 8.42132 2.55518 1.89973
H 9.80735 0.93537 1.74614
C 6.65526 2.23483 3.55977
O 6.63238 0.28019 4.95115
C 5.42817 -0.27752 4.63323
C 8.49688 3.17701 -0.52553
H 9.78907 1.52658 -0.95676
C 6.82641 3.64334 -2.24347
O 6.68251 2.22272 -4.17577
C 5.49388 1.56875 -4.08858
C 9.08266 3.45354 0.85472
C 7.25931 2.97944 2.55709
H 5.77506 2.60140 4.07193
C 3.25701 -1.39693 3.65575
C 7.44320 3.94631 -1.03483
H 5.98880 4.22818 -2.60039
C 3.24413 0.20745 -4.23988
H 8.80326 4.47667 1.10967
O 6.75578 4.23689 2.22706
C 3.22816 -0.16016 4.28742
O 6.98207 5.06279 -0.31915
C 3.29867 1.48986 -3.71359
C 5.61466 4.33374 1.49820
C 5.72764 4.89727 0.19554
C 3.40335 4.25759 1.24427
C 3.50257 4.91992 0.02979
C 10.61548 3.37083 0.88074
H 11.03876 4.08077 0.16530
H 10.99598 2.37870 0.62191
C 10.44105 -0.89792 3.62968
H 10.80373 -0.14901 4.33875
H 10.90074 -0.68460 2.66055
C 10.54445 -3.60686 -0.75783
H 10.94838 -4.31431 -0.02879
H 10.94618 -2.61932 -0.51396
C 10.47192 0.65773 -3.48680
H 10.82047 -0.11005 -4.18270
H 10.91621 0.44460 -2.51037
H 10.85515 1.62706 -3.81643
H 10.91701 -3.87777 -1.74931
H 10.79440 -1.88150 3.95040
H 10.98889 3.61713 1.87834
C -1.84258 3.14934 -3.28228
C -1.83314 4.62787 -1.47567
C -1.87179 -3.08559 3.47230
C -1.91934 -3.90507 1.28418
C 1.89031 -1.67954 3.10651
C 1.81525 0.34899 4.26014
C 1.95630 3.97433 1.51743
C 2.10704 5.17002 -0.48191
C 1.95896 -4.98647 0.45881
C 1.90081 -4.07160 -1.68988
C 1.80854 -0.23821 -4.25596
C -1.96473 -3.98464 -3.00386
C -1.85976 -1.70202 -3.51972
C 1.91308 1.89692 -3.30271
C -1.86784 3.65809 2.78669
C -1.85661 1.35456 3.15246
N -1.10712 2.47161 2.85296
N -1.07514 3.86211 -2.35764
N 1.26915 4.60581 0.50067
N 1.10337 -0.64049 3.55571
N -1.14160 -3.39627 2.30625
N -1.15444 -2.84642 -3.17921
N 1.16882 -4.46066 -0.58519
N 1.10161 0.83763 -3.68698
O -1.42199 5.22804 -0.50160
O -1.42659 2.35868 -4.10188
O -1.43159 -0.56837 -3.61609
O 1.34061 -1.27675 -4.65861
O 1.58151 2.92348 -2.74085
O 1.74397 5.72273 -1.49116
O 1.51112 3.30046 2.43147
O 1.34382 1.36530 4.71063
O -1.44481 4.74654 2.48067
O -1.56882 -4.15125 0.14102
O -1.41321 -2.61093 4.48274
O -1.50248 0.18363 3.12920
O 1.54319 -5.41936 1.50534
O 1.54869 -2.60758 2.38958
O 1.49296 -3.55394 -2.71605
O -1.57731 -5.09063 -2.71315
H -0.06248 3.72213 -2.30159
H 0.08303 0.86347 -3.67553
H -0.13293 -2.92380 -3.11031
H 0.14954 -4.38476 -0.49465
H -0.12945 -3.22042 2.24463
H 0.10468 -0.51520 3.35575
H -0.09634 2.52487 2.68333
H 0.25264 4.64402 0.40735
N -4.51920 -4.14589 -3.12901
N -4.34993 -1.43037 -3.99556
N -4.37562 4.90010 -1.35505
N -4.37116 3.08135 -3.55665
N -4.38839 3.94276 3.10875
N -4.29807 1.19304 3.86518
N -4.41170 -4.43437 1.25064
N -4.40265 -3.23823 3.83868
N 4.34801 -2.16010 3.58665
N 4.32365 0.44259 4.75541
N 4.51704 -5.17785 0.56600
N 4.40389 -4.18105 -2.10678
N 4.45391 3.96175 2.01405
N 4.67420 5.22452 -0.53662
N 4.32806 -0.47175 -4.62136
N 4.42212 2.20431 -3.63857
C 1.73114 -0.18470 -0.21428
C 2.33294 0.58325 0.79118
C 2.56912 -0.85395 -1.11135
C 3.72045 0.66884 0.89510
H 1.72030 1.13593 1.48754
C 3.96003 -0.77130 -1.00459
H 2.14059 -1.45436 -1.90494
C 4.56251 -0.00678 -0.00032
H 4.15324 1.27926 1.68343
H 4.57787 -1.30952 -1.71751
C 6.06254 0.11704 0.09757
H 6.41387 1.04415 -0.36981
H 6.39769 0.13759 1.13803
H 6.56924 -0.71142 -0.40397
C -3.51641 -1.09842 0.43857
C -4.88725 -0.53913 0.84939
C -5.91155 -0.49415 -0.28403
C -2.73249 -0.39749 -0.67540
H -5.26761 -1.17948 1.65232
H -6.88872 -0.17893 0.09239
H -2.00070 -1.09937 -1.06099
H -3.38273 -0.10907 -1.50376
H -2.87092 -1.16101 1.32109
H -3.67224 -2.12083 0.07687
H -4.78155 0.45501 1.28424
H -5.61828 0.21544 -1.06072
H -6.03728 -1.48080 -0.74205
C 0.22944 -0.36602 -0.32132
H -0.00587 -0.82190 -1.28821
H -0.12629 -1.07303 0.44106
N -1.99259 0.81433 -0.26867
C -2.73140 1.97186 -0.07642
H -2.11809 2.81774 0.24424
C -0.58334 0.91775 -0.18946
O -0.06337 2.00688 -0.01210
O -3.93535 2.04092 -0.25586

2·7a@1$_2$

C -8.98352 -3.61802 -0.60403
C -8.32489 -2.78275 -1.69902
C -8.39230 -3.24102 0.74862
H -8.69853 -4.65350 -0.79103
C -8.80394 -1.51891 -2.05674
C -7.20050 -3.26004 -2.38555
C -8.86194 -2.14742 1.48700
C -7.31109 -3.95953 1.27391
C -8.23605 -0.74205 -3.07105
H -9.66745 -1.12811 -1.53232
C -6.60453 -2.53604 -3.41560
O -6.71871 -4.52740 -2.05592
C -8.28498 -1.74447 2.69490
H -9.70901 -1.59054 1.10375
C -6.68534 -3.58549 2.45784
O -6.87031 -5.10400 0.59689
C -8.82510 0.60671 -3.47987
C -7.12785 -1.28495 -3.73908
H -5.74823 -2.93246 -3.94690
C -5.55646 -4.63423 -1.34738
C -8.83322 -0.59483 3.54140
C -7.17540 -2.47665 3.13534
H -5.83306 -4.13591 2.83421
C -5.63520 -5.01358 0.02015
H -8.48428 0.78562 -4.50006
C -8.27716 1.76174 -2.64335
O -6.55890 -0.56330 -4.79706
C -8.24615 0.75741 3.14167
H -8.48972 -0.78189 4.55933
O -6.57098 -2.07668 4.33066
C -8.84457 2.16109 -1.42990
C -7.17338 2.49578 -3.09494
C -5.37722 0.06324 -4.52238
C -3.32898 -4.61637 -1.18380
C -8.81216 1.53736 2.12710
C -7.13198 1.29147 3.80336
C -5.39608 -1.40294 4.22036
C -3.40227 -5.06948 0.12599
C -8.36980 3.25110 -0.69106
H -9.68780 1.60240 -1.04104
C -6.67526 3.59952 -2.41466
O -6.58729 2.10789 -4.30449
C -5.39850 1.45396 -4.22100
C -8.31864 2.79239 1.76093
H -9.68132 1.15302 1.60714

```
C  -6.58513  2.52560  3.46159
O  -6.57050  0.57772  4.87369
C  -5.38169 -0.02851  4.59129
C  -8.96308  3.63144  0.66065
C  -7.28865  3.96566 -1.22139
H  -5.82697  4.15224 -2.79715
C  -3.15834  0.06125 -4.25611
C  -7.18073  3.25213  2.43684
H  -5.71663  2.90887  3.98175
C  -3.21291 -1.28878  3.78544
H  -8.67158  4.66515  0.84790
O  -6.83168  5.10223 -0.53967
C  -3.18978  1.40373 -3.90641
O  -6.68271  4.51415  2.10008
C  -3.17798  0.01927  4.24673
C  -5.59706  4.99160  0.03054
C  -5.51881  4.60253  1.39966
C  -3.36533  5.02012 -0.07431
C  -3.29119  4.56914  1.23677
C -10.49706  3.57050  0.68098
H -10.87271  3.88851  1.65718
H -10.88998  2.56838  0.48762
C -10.36167  0.58825 -3.50682
H -10.73853  1.54220 -3.88488
H -10.80593  0.42785 -2.52047
C -10.51670 -3.54403 -0.63496
H -10.88912 -3.85707 -1.61402
H -10.90302 -2.53949 -0.44105
C -10.36961 -0.58069  3.57173
H -10.74476 -1.53950  3.93928
H -10.81629 -0.40862  2.58833
H -10.72402  0.21072  4.23740
H -10.93582 -4.20857  0.12515
H -10.71636 -0.21128 -4.16259
H -10.90659  4.23637 -0.08327
C   8.98983 -2.12113 -2.99349
C   8.41803 -2.85245 -1.78193
C   8.40860 -0.71111 -3.12066
H   8.64518 -2.67038 -3.87091
C   8.95371 -2.67113 -0.49918
C   7.32139 -3.71796 -1.89582
C   8.99739  0.42299 -2.55260
C   7.23442 -0.50812 -3.85591
C   8.43015 -3.27837  0.64532
H   9.81069 -2.01675 -0.38986
C   6.75191 -4.33246 -0.78530
O   6.75845 -3.99333 -3.15324
C   8.47741  1.71281 -2.72450
H   9.89576  0.30141 -1.95870
C   6.69884  0.74847 -4.09200
O   6.62392 -1.62866 -4.41484
C   9.03463 -3.08755  2.03763
C   7.30025 -4.08643  0.46435
H   5.88833 -4.97570 -0.89448
C   5.52873 -3.41561 -3.32071
C   9.07714  2.94175 -2.05132
C   7.34139  1.84329 -3.53103
H   5.80321  0.87067 -4.68599
C   5.48345 -2.09421 -3.84482
H   8.71024 -3.94432  2.62957
C   8.45517 -1.85139  2.72048
O   6.72604 -4.68491  1.58950
C   8.46578  3.14264 -0.66528
H   8.77046  3.80139 -2.64817
O   6.85449  3.13009 -3.79714
C   8.99920 -0.57265  2.53662
C   7.32938 -1.95054  3.54792
C   5.55374 -4.15858  2.03464
C   3.31760 -3.34884 -3.03105
C   8.97421  2.50232  0.46766
C   7.36713  3.99150 -0.46959
C   5.64181  3.42145 -3.25436
C   3.30786 -2.00545 -3.38012
C   8.44804  0.57703  3.10953
H   9.88759 -0.47256  1.92432
C   6.72258 -0.83523  4.11398
```

```
O   6.79884 -3.21706  3.83525
C   5.58110 -3.45583  3.27059
C   8.47234  2.70485  1.75827
H   9.80331  1.81614  0.34163
C   6.84561  4.24795  0.79116
O   6.80960  4.64093 -1.57649
C   5.61984  4.17259 -2.04518
C   9.05717  1.97086  2.96120
C   7.28194  0.40826  3.86612
H   5.83486 -0.93628  4.72408
C   3.35295 -3.82157  1.92189
C   7.41309  3.61144  1.89074
H   6.01158  4.92685  0.91694
C   3.41635  3.30544 -3.21070
H   8.74590  2.53338  3.84249
O   6.71997  1.54793  4.43986
C   3.36537 -3.21633  3.17110
O   6.89706  3.90894  3.16287
C   3.39620  3.99187 -2.00468
C   5.57922  2.05930  3.91385
C   5.65712  3.37391  3.37270
C   3.38432  2.06286  3.54569
C   3.43754  3.39514  3.16076
C  10.59292  1.94744  2.94697
H  10.98156  2.96851  2.91000
H  11.00459  1.40811  2.08925
C  10.57021 -3.09379  2.01576
H  10.95973 -3.01291  3.03408
H  10.99299 -2.26943  1.43457
C  10.52557 -2.12959 -3.01627
H  10.89406 -3.15854 -2.99427
H  10.96829 -1.60366 -2.16563
C  10.61236  2.92351 -2.02020
H  11.00529  2.83864 -3.03693
H  11.01926  2.09110 -1.43925
H  10.99002  3.84864 -1.57682
H  10.89048 -1.64786 -3.92737
H  10.93491 -4.02580  1.57597
H  10.96953  1.46359  3.85219
C  -1.96468  5.13227 -0.61922
C  -1.84813  4.29024  1.56422
C  -1.99757 -5.15745  0.67278
C  -1.90174 -4.25221 -1.47846
C   1.94710 -3.79716  1.39695
C   1.95060 -2.84230  3.52566
C   1.96670  1.58939  3.42149
C   2.03690  3.86474  2.87741
C   1.90197 -3.77415 -2.75105
C   1.91190 -1.47656 -3.20751
C   2.00113  2.98016 -3.59642
C  -1.72458 -0.39309 -4.23311
C  -1.78679  1.84206 -3.59217
C   1.96852  4.09202 -1.54310
C  -1.76911  0.52448  4.12013
C  -1.84339 -1.66121  3.30086
N  -1.06417 -0.54007  3.53767
N  -1.14707  4.66788  0.42652
N   1.23849  2.72128  3.08175
N   1.19656 -3.22664  2.40305
N  -1.18768 -4.66390 -0.36710
N  -0.99387  0.76069 -3.93187
N   1.15345 -2.58385 -2.85918
N   1.22303  3.49417 -2.54281
O  -1.40094  3.79045  2.57683
O  -1.58696  5.52119 -1.69796
O  -1.44384  2.91266 -3.12646
O   1.57993  2.41265 -4.57590
O   1.55970  4.55456 -0.49145
O   1.64480  4.95618  2.54250
O   1.56049  0.44795  3.53498
O   1.51182 -2.33396  4.52961
O  -1.30970  1.59958  4.42847
O  -1.48896 -3.64385 -2.44827
O  -1.61338 -5.54967  1.74733
O  -1.50319 -2.71443  2.79438
O   1.46516 -4.87331 -2.51304
```

```
O   1.55714 -4.17898  0.30596
O   1.54509 -0.32145 -3.31728
O  -1.27646 -1.49922 -4.43030
H  -0.12809  4.63860  0.32333
H   0.19694  3.43340 -2.58106
H   0.00170  0.68003 -3.71935
H   0.13361 -2.61006 -2.80803
H  -0.17795 -4.51917 -0.25105
H   0.17613 -3.09191  2.37926
H  -0.05174 -0.46624  3.41246
H   0.21881  2.78603  3.06329
N  -4.25471 -0.64353 -4.54366
N  -4.30838  2.12747 -3.87955
N  -4.36466  4.36504  2.00456
N  -4.52284  5.22693 -0.70800
N  -4.26718  0.68546  4.63311
N  -4.31917 -2.03245  3.77467
N  -4.39962 -4.40362 -1.95098
N  -4.56067 -5.26189  0.75794
N   4.44238 -4.31757  1.33179
N   4.48634 -3.00278  3.86323
N   4.44037 -4.07140 -2.95223
N   4.38679 -1.35417 -3.82155
N   4.45030  1.36947  3.95081
N   4.58451  4.06670  3.02569
N   4.54150  2.99454 -3.85515
N   4.49642  4.44383 -1.39661
C  -5.59041 -0.74832  0.17027
C   0.55268  0.95699  0.18344
H  -5.04111 -1.10700  1.04130
H   0.29464  1.15127  1.23168
H   0.40495  1.89100 -0.36085
C   2.01141  0.54096  0.11323
C   2.44137 -0.77534 -0.07896
C   2.98655  1.53657  0.26629
C   3.80449 -1.07615 -0.13513
H   1.71388 -1.56808 -0.19664
C   4.34487  1.23124  0.22008
H   2.68099  2.56478  0.42260
C   4.77972 -0.08317  0.01408
H   4.11399 -2.10437 -0.30259
H   5.07539  2.02720  0.33855
C   6.24740 -0.41483 -0.02379
H   6.65267 -0.52869  0.98706
H   6.42929 -1.34922 -0.55682
H   6.82559  0.37299 -0.51470
C  -5.68276  0.78146  0.13283
H  -6.34942  1.06965 -0.68728
H  -6.16775  1.11403  1.05499
N  -4.88393 -1.24715 -1.00431
H  -1.39741 -1.61642  0.14026
C  -0.49988 -0.00782 -0.31636
O  -0.52764 -1.16119  0.35296
O  -1.30353  0.27630 -1.19092
H  -5.32554 -1.13667 -1.90639
C  -3.61967 -1.67979 -0.98671
H  -3.19004 -1.85479 -1.97982
O  -2.98855 -1.91362  0.05826
C  -4.33012  1.47967 -0.04090
H  -3.84643  1.13369 -0.95930
H  -4.51316  2.54553 -0.19378
C  -3.37359  1.27180  1.13130
H  -3.77929  1.68720  2.05762
H  -3.16636  0.20945  1.27605
H  -2.41531  1.75757  0.93987
H  -6.58877 -1.19067  0.19964
```

7a·7a@1$_2$

```
C  -8.91696 -3.58575 -0.22488
C  -8.31750 -2.88511 -1.44113
C  -8.31241 -3.02456  1.05746
```

H	-8.60257	-4.62815	-0.28386
C	-8.82410	-1.68229	-1.94042
C	-7.21116	-3.43166	-2.10277
C	-8.86108	-1.91780	1.71442
C	-7.16885	-3.60352	1.62311
C	-8.29417	-1.03287	-3.06155
H	-9.66772	-1.22876	-1.43284
C	-6.65185	-2.83745	-3.22568
O	-6.69573	-4.64816	-1.64062
C	-8.32160	-1.38669	2.88867
H	-9.75331	-1.46135	1.30259
C	-6.57505	-3.09942	2.77536
O	-6.65963	-4.77385	1.05019
C	-8.88329	0.27624	-3.58457
C	-7.20535	-1.64910	-3.69233
H	-5.80265	-3.28452	-3.72709
C	-5.48573	-4.63240	-1.02039
C	-8.94907	-0.20957	3.63512
C	-7.15956	-1.99340	3.37971
H	-5.69205	-3.56440	3.19417
C	-5.46526	-4.71671	0.40191
H	-8.55209	0.36411	-4.62024
C	-8.31851	1.49619	-2.85557
O	-6.64779	-1.06709	-4.84133
C	-8.37876	1.11115	3.12764
H	-8.63493	-0.30274	4.67538
O	-6.62256	-1.48300	4.56538
C	-8.88168	2.02610	-1.69221
C	-7.18148	2.14471	-3.35307
C	-5.43536	-0.48034	-4.60763
C	-3.25318	-4.65613	-1.03210
C	-8.89803	1.74754	1.99162
C	-7.29903	1.73099	3.76768
C	-5.47090	-0.77383	4.49412
C	-3.23116	-4.76755	0.35158
C	-8.37458	3.16058	-1.04233
H	-9.74412	1.52893	-1.26330
C	-6.67206	3.30262	-2.78570
O	-6.56505	1.60402	-4.48201
C	-5.41366	0.90536	-4.29211
C	-8.36863	2.93113	1.47102
H	-9.75101	1.30030	1.49442
C	-6.71697	2.89253	3.27538
O	-6.77184	1.17519	4.94703
C	-5.53727	0.61894	4.79285
C	-8.96947	3.66435	0.26919
C	-7.28298	3.80259	-1.64066
H	-5.80574	3.79330	-3.20918
C	-3.22940	-0.57168	-4.28245
C	-7.24355	3.45207	2.12210
H	-5.85799	3.33158	3.76483
C	-3.26883	-0.58488	4.23993
H	-8.66174	4.70655	0.36539
O	-6.76853	4.98773	-1.08035
C	-3.23486	0.74592	-3.84362
O	-6.65439	4.59805	1.58600
C	-3.31434	0.74764	4.62089
C	-5.54576	4.80269	-0.49947
C	-5.51077	4.43006	0.87356
C	-3.33920	4.49779	-0.61428
C	-3.34389	3.95361	0.66185
C	-10.50526	3.63476	0.27582
H	-10.88517	4.06148	1.20791
H	-10.91373	2.62447	0.18602
C	-10.41982	0.26234	-3.59258
H	-10.80102	1.18571	-4.03681
H	-10.85156	0.17582	-2.59158
C	-10.45223	-3.56529	-0.22713
H	-10.82949	-4.04045	-1.13645
H	-10.86779	-2.55476	-0.18960
C	-10.48426	-0.24293	3.61349
H	-10.84526	-1.18539	4.03384
H	-10.90126	-0.15005	2.60712
H	-10.88348	0.58048	4.21150
H	-10.83696	-4.11324	0.63722
H	-10.78329	-0.58386	-4.18152
H	-10.89373	4.22230	-0.56019
C	9.00864	-2.43605	-2.64602
C	8.43257	-2.97844	-1.34141
C	8.38019	-1.08140	-2.97065
H	8.69054	-3.12072	-3.43282
C	8.94897	-2.59696	-0.09625
C	7.34458	-3.85927	-1.33601
C	8.91921	0.13282	-2.53313
C	7.20760	-1.01714	-3.73156
C	8.41413	-3.03489	1.11760
H	9.80465	-1.93223	-0.07594
C	6.75422	-4.29941	-0.15721
O	6.82454	-4.32794	-2.55381
C	8.34299	1.36936	-2.84053
H	9.82915	0.11532	-1.94508
C	6.58649	0.18163	-4.05981
O	6.69379	-2.22374	-4.22051
C	9.01646	-2.66917	2.47434
C	7.28387	-3.85888	1.04571
H	5.89300	-4.95387	-0.17972
C	5.59199	-3.83628	-2.86484
C	8.94024	2.69802	-2.38726
C	7.16864	1.35805	-3.60438
H	5.68473	0.20173	-4.65732
C	5.53175	-2.67797	-3.69378
H	8.71124	-3.45682	3.16437
C	8.41381	-1.37493	3.00846
O	6.70648	-4.28155	2.24441
C	8.34101	3.12474	-1.05092
H	8.62270	3.44286	-3.11776
O	6.63837	2.58721	-3.99031
C	8.90212	-0.11842	2.62432
C	7.33354	-1.39571	3.89806
C	5.55909	-3.67612	2.64637
C	3.36712	-3.80576	-2.68866
C	8.82567	2.62939	0.16209
C	7.27260	4.02776	-0.98572
C	5.46598	3.02425	-3.45679
C	3.32124	-2.64209	-3.43960
C	8.33391	1.08140	3.05830
H	9.75857	-0.07843	1.96114
C	6.71061	-0.23020	4.33174
O	6.84293	-2.62669	4.37048
C	5.61414	-2.93108	3.85782
C	8.30595	2.99932	1.40627
H	9.64700	1.92228	0.13836
C	6.71410	4.42433	0.22396
O	6.76324	4.57116	-2.17026
C	5.53137	4.11111	-2.54136
C	8.89308	2.45747	2.70457
C	7.20651	0.98268	3.88130
H	5.85154	-0.27237	4.98791
C	3.36669	-3.27786	2.52838
C	7.23712	3.90489	1.40225
H	5.88313	5.11871	0.24597
C	3.23739	3.09237	-3.35340
H	8.55684	3.13129	3.49352
O	6.59113	2.17298	4.27705
C	3.39832	-2.68794	3.78638
O	6.66484	4.31529	2.62086
C	3.30064	4.18052	-2.49239
C	5.44717	2.51993	3.63328
C	5.46019	3.72174	2.86876
C	3.27850	2.28265	3.17769
C	3.25704	3.52413	2.55700
C	10.42975	2.48016	2.70318
H	10.78807	3.49576	2.51559
H	10.86575	1.83144	1.93837
C	10.55195	-2.64479	2.45030
H	10.93724	-2.44355	3.45339
H	10.95770	-1.87948	1.78284
C	10.54351	-2.40389	-2.65348
H	10.93873	-3.40673	-2.47109
H	10.96341	-1.74223	-1.89078
C	10.47562	2.69347	-2.36794
H	10.86368	2.42207	-3.35354
H	10.89349	1.98965	-1.64260
H	10.84775	3.68833	-2.10954
H	10.90672	-2.05808	-3.62498
H	10.93833	-3.61140	2.11615
H	10.81103	2.14934	3.67305
C	-1.93396	4.44394	-1.14489
C	-1.97535	3.40286	0.93461
C	-1.79221	-4.81646	0.80478
C	-1.83604	-4.60683	-1.52836
C	1.95557	-3.20732	2.00830
C	1.99409	-2.32241	4.17745
C	1.89686	1.70553	3.13123
C	1.83191	3.83497	2.18232
C	1.95924	-4.15954	-2.28979
C	1.90476	-2.16479	-3.50394
C	1.78732	2.78151	-3.62535
C	-1.80721	-1.06907	-4.26098
C	-1.83465	1.10851	-3.42816
C	1.89681	4.63729	-2.19601
C	-1.90321	1.28173	4.61016
C	-1.85611	-0.93917	3.91014
N	-1.11881	0.18570	4.17164
N	-1.20143	3.78630	-0.14095
N	1.10585	2.70112	2.61383
N	1.22443	-2.67136	3.05479
N	-1.05426	-4.76029	-0.38401
N	-1.06241	0.03469	-3.82911
N	1.16722	-3.12961	-2.81947
N	1.08943	3.78977	-2.97101
O	-1.62734	2.71204	1.88365
O	-1.51280	4.85891	-2.19855
O	-1.46710	2.10311	-2.83290
O	1.32418	1.86231	-4.26720
O	1.52274	5.52581	-1.46964
O	1.36038	4.81312	1.66129
O	1.55079	0.58050	3.47205
O	1.57369	-1.85449	5.20805
O	-1.48214	2.37165	4.90119
O	-1.43857	-4.43141	-2.66092
O	-1.34762	-4.86546	1.92867
O	-1.46263	-2.00829	3.45545
O	1.57128	-5.10404	-1.63738
O	1.53782	-3.50223	0.90227
O	1.50623	-1.12839	-3.99151
O	-1.37791	-2.16165	-4.55142
H	-0.20452	3.50706	-0.22527
H	0.07315	3.83417	-2.91777
H	-0.04815	-0.01301	-3.74848
H	0.14780	-3.19213	-2.85216
H	-0.03495	-4.78904	-0.42761
H	0.20900	-2.54069	3.06259
H	-0.10529	0.26312	3.99013
H	0.10697	2.60230	2.39650
N	-4.33967	-1.22389	-4.63504
N	-4.32448	1.51747	-3.84884
N	-4.42394	3.93535	1.44692
N	-4.45126	4.89548	-1.23796
N	-4.45901	1.38425	4.88554
N	-4.33803	-1.37945	4.17482
N	-4.37859	-4.57884	-1.74614
N	-4.33845	-4.78122	1.09896
N	4.44482	-3.80474	1.94072
N	4.52973	-2.47109	4.46047
N	4.51104	-4.41840	-2.36723
N	4.39712	-2.05145	-3.96073
N	4.36742	1.76094	3.74514
N	4.35869	4.25271	2.35938
N	4.31977	2.48879	-3.85218
N	4.44999	4.69991	-2.05389
C	2.87695	0.26222	-1.15432
H	3.20014	1.26966	-0.88597
C	3.53261	-0.78079	-0.24235
H	3.13643	-1.77419	-0.48325
H	3.23536	-0.57951	0.79601
N	1.42303	0.26015	-1.04854
H	0.86926	-0.54346	-1.33828

```
C   0.71458  1.32621 -0.66107
H  -0.36788  1.15037 -0.67942
O   1.19502  2.41959 -0.31944
C  -3.53996 -1.36324  0.26841
H  -4.27289 -1.98345  0.79380
C  -3.80107  0.11931  0.58987
H  -3.37541  0.74411 -0.20575
H  -3.26621  0.38801  1.50693
N  -2.22412 -1.82432  0.68804
H  -2.11787 -2.16093  1.64033
C  -1.10729 -1.70261 -0.04758
H  -0.20937 -2.07888  0.46296
O  -1.06243 -1.24660 -1.19820
C  -5.29443  0.40966  0.76646
H  -5.42606  1.45107  1.07310
H  -5.67492 -0.20217  1.59417
C  -6.12722  0.13438 -0.48371
H  -5.79665  0.76307 -1.31626
H  -6.06013 -0.90951 -0.80568
H  -7.18141  0.35069 -0.30467
C   5.06461 -0.79216 -0.35906
H   5.44700 -1.70142  0.11597
H   5.34492 -0.86061 -1.41751
C   5.75141  0.42563  0.26732
H   5.44578  1.36528 -0.20535
H   5.51529  0.49693  1.33465
H   6.83789  0.35435  0.17204
H   3.15840  0.09384 -2.19994
H  -3.64202 -1.55686 -0.80245
```

3b

```
C -0.32352 -0.00001 -0.40295
N  1.07714 -0.00007 -0.09618
C  2.21998 -0.00008  0.18715
C -0.97376 -1.27470  0.14671
H -0.48791 -2.16570 -0.25811
H -0.89404 -1.30142  1.23725
H -2.03243 -1.29699 -0.12693
C -0.97356  1.27480  0.14670
H -0.48729  2.16569 -0.25785
H -2.03215  1.29747 -0.12725
H -0.89426  1.30133  1.23728
H -0.40672  0.00001 -1.49680
```

2·3b

```
C -1.46284 -2.42919 -0.07107
N -2.52457 -1.62647 -0.61351
C -3.30162 -0.83844 -0.99843
H -2.94706  1.12074 -0.96255
C -0.36379  2.59639  0.27056
C -1.65144  1.78771  0.24869
O -2.24268  1.81137 -0.95415
O -2.05362  1.14220  1.19672
C -0.17131 -2.12829 -0.84059
H -0.29283 -2.33918 -1.90614
H  0.10873 -1.08065 -0.71873
C  0.80209  1.63285  0.15914
C  1.45831  1.43140 -1.05767
C  1.20019  0.87389  1.26752
C  2.49382  0.50121 -1.16225
H  1.15719  2.00500 -1.92976
C  2.23164 -0.05453  1.15815
H  0.68749  1.00968  2.21476
C  2.89545 -0.25947 -0.05937
H  2.99448  0.36320 -2.11695
H  2.52777 -0.63055  2.03130
C  3.98688 -1.29414 -0.17978
H  4.63488 -1.29757  0.70223
H  3.56578 -2.30292 -0.27532
H  4.61148 -1.11584 -1.05931
H -0.35597  3.31426 -0.55153
H -0.32484  3.13601  1.22046
H  0.63930 -2.74950 -0.45025
C -1.33557 -2.14647  1.43035
H -2.26403 -2.39179  1.95173
H -0.52706 -2.75543  1.84482
H -1.11676 -1.08975  1.59510
H -1.74708 -3.47657 -0.22727
```

(*Z*)-4b-TS

```
C -3.57420 -1.49369 -0.31733
N -3.47977 -0.19858 -0.99164
C -2.84610  0.79825 -0.97113
H -2.25396  1.83556 -1.10933
C  0.59960  2.09741  0.88021
C -0.72733  1.82167  0.17594
O -1.13355  2.62971 -0.71094
O -1.35783  0.76326  0.49949
C -3.87920 -1.25744  1.16475
H -3.06399 -0.69184  1.62122
H -4.81296 -0.70299  1.29015
C  1.62180  1.03470  0.53946
C  2.65574  1.29799 -0.36270
C  1.52739 -0.25205  1.08553
C  3.57537  0.30619 -0.70549
H  2.73916  2.28692 -0.80547
C  2.44553 -1.23966  0.74035
H  0.71604 -0.47658  1.77037
C  3.48887 -0.97782 -0.15841
H  4.37117  0.53408 -1.41024
H  2.35402 -2.23212  1.17542
C  4.50197 -2.04246 -0.50126
H  4.97279 -1.84902 -1.46952
H  5.30211 -2.08600  0.24825
H  4.04187 -3.03484 -0.54000
H  0.94914  3.08787  0.58325
H  0.40525  2.10222  1.95844
C -2.26040 -2.25145 -0.53668
H -2.06594 -2.39895 -1.60223
H -1.43218 -1.68908 -0.09917
H -2.32700 -3.23293 -0.05828
H -3.97918 -2.22264  1.66954
H -4.40121 -2.03315 -0.78873
```

(*Z*)-4b

```
C  1.60964  2.39544 -0.15570
N  2.77241  1.56596 -0.51438
C  2.78087  0.31647 -0.39819
H  3.62614 -0.30393 -0.68253
C  0.45224 -2.36400  0.62756
C  1.71562 -1.77881  0.01504
O  2.61597 -2.41370 -0.47665
O  1.70260 -0.41329  0.11193
C  0.38749  2.08613 -1.03344
H  0.65574  2.12931 -2.09327
H -0.02216  1.09869 -0.81524
H  1.92233  3.42335 -0.37394
C -0.77803 -1.51968  0.39229
C -1.40029 -1.51038 -0.86043
C -1.27732 -0.67716  1.38922
C -2.48378 -0.67076 -1.11017
H -1.02417 -2.15383 -1.65189
C -2.36122  0.16163  1.13754
H -0.80384 -0.66556  2.36728
C -2.98219  0.18132 -0.11668
H -2.94706 -0.67220 -2.09325
H -2.72646  0.81556  1.92501
C -4.16695  1.07620 -0.38316
H -5.10719  0.57969 -0.11342
H -4.10812  1.99968  0.20038
H -4.23270  1.34617 -1.44103
H  0.34883 -3.37283  0.22042
H  0.64187 -2.46769  1.70300
C  1.27782  2.32427  1.34167
H  0.86288  1.34985  1.60672
H  2.17281  2.50366  1.94491
H  0.53817  3.09180  1.59196
H -0.39605  2.82847 -0.84994
```

(*E*)-4b-TS

```
C  3.95012 -1.06714 -0.33299
H  4.82970 -1.00079 -0.99533
N  3.06714  0.00794 -0.58691
C  2.26921  0.91216 -0.74596
H  2.20789  1.65614 -1.54411
C -0.83759  2.05222  0.95415
C  0.31533  2.12838 -0.03269
O  0.41662  2.94344 -0.92163
O  1.20311  1.12256  0.20296
C  3.22583 -2.38639 -0.64521
H  2.36281 -2.50693  0.01621
H  2.87388 -2.40111 -1.68003
C -1.75191  0.89180  0.61081
C -1.43299 -0.41403  1.00224
C -2.91003  1.09884 -0.14321
C -2.25771 -1.47959  0.65268
H -0.52887 -0.59215  1.57634
C -3.73389  0.02847 -0.49081
H -3.17059  2.10543 -0.45855
C -3.42136 -1.27824 -0.10214
H -1.99727 -2.48539  0.97271
H -4.63423  0.21206 -1.07147
C -4.29387 -2.44202 -0.50348
H -3.93519 -2.90155 -1.43296
H -4.29549 -3.22306  0.26301
H -5.32794 -2.12838 -0.67187
H -0.42697  1.92898  1.96111
H -1.37476  3.00078  0.90255
H  3.90892 -3.22792 -0.49643
C  4.44300 -1.03449  1.12182
H  5.16155 -1.84235  1.29449
H  4.93460 -0.08304  1.34291
H  3.59917 -1.15412  1.80792
```

(*E*)-4b

```
C  4.12228  0.78034 -0.37975
N  2.94005  0.35291  0.36720
C  2.19984 -0.50306 -0.17913
H  2.32960 -0.98127 -1.15261
C -0.98259 -2.06738  0.72672
C  0.27535 -1.86932 -0.09141
O  0.54667 -2.42578 -1.12713
O  1.06980 -0.91584  0.49938
C  4.01754  2.28717 -0.63329
H  3.12870  2.52146 -1.22636
H  3.94429  2.82489  0.31692
H  4.18528  0.26359 -1.35377
C -1.96039 -0.93668  0.46009
C -2.99304 -1.09719 -0.46731
C -1.81577  0.30088  1.09800
C -3.87065 -0.04852 -0.74047
```

```
H -3.10948 -2.04721 -0.98158
C -2.69378  1.34498  0.82073
H -1.00261  0.44790  1.80225
C -3.73883  1.18810 -0.09974
H -4.66901 -0.19313 -1.46360
H -2.56578  2.29910  1.32549
C -4.70662  2.31347 -0.37025
H -5.49821  2.34494  0.38857
H -4.20374  3.28521 -0.35333
H -5.19127  2.19818 -1.34382
H -1.41196 -3.02776  0.43688
H -0.71290 -2.09919  1.78633
C  5.36819  0.43116  0.43957
H  6.27353  0.76080 -0.07990
H  5.32641  0.92220  1.41656
H  5.43682 -0.64819  0.60377
H  4.89917  2.64748 -1.17280
```

5b-TS

```
C  0.92526  2.07591  1.54381
C  1.59895  1.97471  0.17220
H  2.48794  2.62471  0.16335
N  2.04435  0.60255 -0.02797
C  3.14719  0.19186 -0.55397
H  3.92562  0.82573 -0.99216
C  0.73286 -1.68339 -0.70607
C  1.73635 -1.25789  0.33875
O  1.82721 -1.58698  1.47561
O  3.28992 -1.09213 -0.53326
H  0.03737  1.43698  1.57155
H  1.60460  1.74968  2.33555
C -0.64759 -1.12447 -0.43476
C -1.38695 -0.55725 -1.47518
C -1.21395 -1.15552  0.84695
C -2.65929 -0.03109 -1.24545
H -0.96322 -0.52138 -2.47526
C -2.47986 -0.62236  1.07259
H -0.64911 -1.58347  1.66818
C -3.22524 -0.04896  0.03245
H -3.21542  0.40549 -2.07079
H -2.89897 -0.65119  2.07531
C -4.58983  0.53842  0.29551
H -5.08493  0.83041 -0.63444
H -4.52215  1.42912  0.93114
H -5.23830 -0.17677  0.81288
H  0.72445 -2.78080 -0.67289
H  1.08341 -1.38193 -1.69234
H  0.61823  3.10693  1.74406
C  0.65815  2.41836 -0.95385
H  0.35418  3.45991 -0.80707
H -0.23856  1.79387 -0.97007
H  1.15112  2.33784 -1.92783
```

5b

```
C  2.54421 -1.50925 -1.50110
C  0.00061  1.79091  0.78406
H -0.14140  2.84844  0.54667
H  0.19778  1.72035  1.85746
C -1.23741  0.99190  0.41737
C -2.07395  0.46207  1.40226
C -1.56331  0.77104 -0.92698
C -3.20973 -0.27006  1.05458
H -1.83687  0.61916  2.45172
C -2.69522  0.03748 -1.26898
H -0.92321  1.17574 -1.70598
C -3.53997 -0.49552 -0.28517
H -3.84419 -0.67670  1.83745
```

```
H -2.92792 -0.12717 -2.31792
C -4.77597 -1.27156 -0.66704
H -5.15731 -1.85491  0.17531
H -5.57866 -0.59956 -0.99405
H -4.57453 -1.96023 -1.49358
H  1.61331 -1.31617 -2.04291
H  2.40941 -2.38854 -0.86950
N  1.82776  0.13706  0.21879
C  1.38563 -0.71543  1.22221
H  0.53734 -0.32185  1.79579
C  1.21574  1.37615 -0.04220
O  1.61364  2.09208 -0.94572
O  1.87098 -1.80432  1.45908
C  2.94631 -0.28683 -0.67228
H  3.07315  0.56258 -1.34267
C  4.24265 -0.48570  0.11583
H  4.16808 -1.33393  0.79735
H  5.06310 -0.67007 -0.58467
H  4.48607  0.41232  0.69167
H  3.32690 -1.72208 -2.23579
```

2·(*E*)-4b

```
C -5.28594 -0.67250  0.38916
N -3.86314 -0.78532  0.06035
C -3.49986 -1.70541 -0.72450
H -4.18155 -2.42614 -1.18630
C  0.09836 -1.49039 -1.30550
C -1.11202 -1.54693 -0.40346
O -1.16333 -1.34286  0.77601
O -2.23203 -1.91542 -1.16242
C -5.44816 -0.82881  1.90353
H -5.08095 -1.80340  2.23671
H -4.87868 -0.05454  2.42685
H -5.85737 -1.46893 -0.11578
C  1.40934 -1.70319 -0.59067
C  1.80923 -0.85949  0.45291
C  2.26499 -2.73739 -0.97676
C  3.02836 -1.05886  1.09162
H  1.16985 -0.04159  0.76138
C  3.49261 -2.92705 -0.34043
H  1.97388 -3.39933 -1.78866
C  3.89301 -2.09332  0.70788
H  3.32041 -0.38579  1.89302
H  4.14546 -3.73423 -0.66262
C  5.20819 -2.30032  1.41847
H  5.73962 -1.35266  1.55735
H  5.05746 -2.73336  2.41474
H  5.86241 -2.97593  0.86042
H -0.03676 -2.20657 -2.11973
H  0.04999 -0.48932 -1.75416
C -5.78989  0.68832 -0.10246
H -5.22148  1.49546  0.36913
H -5.67228  0.77846 -1.18606
H -6.84794  0.81462  0.14588
H -6.50148 -0.73661  2.18578
C -0.87372  3.01330  0.00134
C -1.69472  1.75499 -0.24416
O -1.64502  1.12835 -1.29329
O -2.44264  1.40449  0.79388
C  0.60964  2.71201 -0.07101
C  1.19822  2.29895 -1.27472
C  1.41869  2.81739  1.06238
C  2.55350  1.99187 -1.33149
H  0.57788  2.20306 -2.15938
C  2.78125  2.51773  0.99738
H  0.98164  3.13634  2.00480
C  3.36952  2.09169 -0.19615
H  2.98820  1.65956 -2.27072
H  3.39221  2.60766  1.89198
C  4.82246  1.69635 -0.25852
H  5.37586  2.06309  0.61069
```

```
H  4.91691  0.60419 -0.27926
H  5.30740  2.08522 -1.15972
H -1.14997  3.73194 -0.77887
H -1.13693  3.43983  0.97053
H -2.90609  0.53611  0.56706
```

7b-TS

```
C -5.35153 -0.54088 -0.01778
N -4.05144  0.06327  0.29268
C -3.78621  0.64663  1.42449
H -4.59165  0.70538  2.16476
C -0.33157  1.89560  1.42762
C -1.50997  1.46801  0.59521
O -1.95380  1.91102 -0.41561
O -2.67241  1.16532  1.78027
C -5.91152  0.10845 -1.28577
H -6.03692  1.18605 -1.15045
H -5.22835 -0.05385 -2.12504
H -6.02318 -0.33473  0.82574
C  0.96783  1.90801  0.65317
C  2.11226  1.34424  1.22355
C  1.07110  2.49305 -0.61310
C  3.32980  1.37003  0.55103
H  2.04698  0.86782  2.19693
C  2.28940  2.49880 -1.29056
H  0.19154  2.92521 -1.07730
C  3.43787  1.93718 -0.72290
H  4.20566  0.92585  1.01380
H  2.34777  2.94714 -2.27927
C  4.74384  1.89619 -1.47517
H  4.77701  2.65064 -2.26640
H  5.59687  2.06273 -0.80956
H  4.88857  0.91587 -1.94603
H -0.58343  2.90087  1.79208
H -0.25976  1.23572  2.29055
C -5.17033 -2.05435 -0.17308
H -4.43304 -2.26309 -0.95355
H -4.81634 -2.50182  0.76018
H -6.11878 -2.52482 -0.44799
H -6.88146 -0.32923 -1.53919
C -0.15570 -1.92861 -1.18110
C -1.19562 -0.92557 -0.67229
O -0.96741 -0.34265  0.44684
O -2.23024 -0.77594 -1.36244
C  1.21650 -1.89186 -0.55892
C  1.40641 -2.15186  0.80463
C  2.34652 -1.66244 -1.34724
C  2.68547 -2.19551  1.35089
H  0.54106 -2.31697  1.43811
C  3.62838 -1.71167 -0.79950
H  2.22380 -1.45386 -2.40681
C  3.82057 -1.98040  0.55808
H  2.80874 -2.40534  2.41088
H  4.49152 -1.54238 -1.43851
C  5.20338 -2.00133  1.16065
H  5.96272 -2.24598  0.41212
H  5.46871 -1.02382  1.58419
H  5.27689 -2.73308  1.97115
H -0.60895 -2.91905 -1.03591
H -0.09775 -1.79024 -2.26451
H -3.27881 -0.12024 -0.43290
```

7b

```
C -0.69940  0.04907 -0.27108
N  0.35205 -0.66704  0.45575
C  1.67272 -0.41816  0.25104
H  2.32229 -1.01763  0.92059
```

```
O  2.11463  0.35898 -0.58030
C -1.96614 -0.80447 -0.29918
H -1.77609 -1.77948 -0.75732
H -2.35119 -0.96816  0.71523
H -0.31933  0.17571 -1.28960
C -0.93987  1.43838  0.33006
H -1.29685  1.35886  1.36319
H -0.01106  2.01334  0.32287
H -1.69080  1.98505 -0.24970
H -2.75134 -0.30174 -0.87049
H  0.08913 -1.25746  1.23232
```

2·3b@1$_2$

```
C  8.97758 -2.42761  2.79712
C  8.37275 -1.04225  3.04892
C  8.41135 -3.06017  1.52609
H  8.64519 -3.05670  3.62432
C  8.95097  0.15688  2.61739
C  7.17276 -0.93883  3.76267
C  8.92305 -2.72576  0.26461
C  7.34040 -3.96552  1.55303
C  8.38705  1.41117  2.89292
H  9.87264  0.11489  2.04850
C  6.59268  0.27506  4.10034
O  6.59033 -2.12576  4.19574
C  8.40299 -3.21416 -0.93737
H  9.75252 -2.02942  0.21930
C  6.77659 -4.46760  0.38371
O  6.78369 -4.39030  2.77346
C  8.97536  2.72169  2.38141
C  7.21886  1.43518  3.66441
H  5.67948  0.31787  4.67818
C  5.46738 -2.56774  3.57821
C  8.99397 -2.83161 -2.29588
C  7.29936 -4.07395 -0.83958
H  5.93248 -5.14438  0.42825
C  5.53869 -3.85639  2.98247
H  8.64589  3.49680  3.07426
C  8.38974  3.09323  1.01848
O  6.69408  2.67778  4.03689
C  8.40177 -1.51894 -2.80505
H  8.67077 -3.60374 -2.99511
O  6.72100 -4.57890 -2.01002
C  8.90638  2.57127 -0.17020
C  7.32045  3.99267  0.89608
C  5.50183  3.01796  3.47489
C  3.30292 -2.47365  3.06880
C  8.95916 -0.26771 -2.51174
C  7.24669 -1.52973 -3.59612
C  5.52430 -4.04006 -2.38087
C  3.33070 -3.80062  2.66144
C  8.44042  2.93314 -1.43944
H  9.70990  1.84631 -0.10726
C  6.84438  4.41250 -0.33881
O  6.71657  4.49889  2.05346
C  5.51821  3.93917  2.39165
C  8.39603  0.93315 -2.95467
H  9.87083 -0.22943 -1.92721
C  6.62200 -0.36447 -4.02697
O  6.73009 -2.76768 -3.99338
C  5.52841 -3.10640 -3.45372
C  9.02172  2.30309 -2.69989
C  7.41441  3.88428 -1.49287
H  6.02553  5.11801 -0.40174
C  3.28426  2.85099  3.28698
C  7.20155  0.84737 -3.68151
H  5.71628 -0.40424 -4.61712
C  3.30864 -3.81689 -2.23073
H  8.71391  2.93883 -3.53115
O  6.91568  4.31741 -2.73306
C  3.30718  3.69224  2.18181
O  6.65418  2.04074 -4.14899
C  3.31243 -2.92718 -3.29719
C  5.65679  3.83983 -2.98125
C  5.53360  2.55553 -3.58492
C  3.43901  3.91918 -2.74706
C  3.34099  2.60379 -3.17948
C 10.55694  2.26973 -2.68729
H 10.93216  1.85293 -3.62585
H 10.96628  1.66788 -1.87110
C 10.51115  2.72984  2.37374
H 10.87893  3.71229  2.06663
H 10.93976  1.99147  1.69040
C 10.51309 -2.41548  2.81337
H 10.87617 -2.00936  3.76133
H 10.94520 -1.81327  2.00917
C 10.52957 -2.83049 -2.28453
H 10.90238 -3.81198 -1.98029
H 10.95240 -2.09203 -1.59756
H 10.91151 -2.60750 -3.28441
H 10.89477 -3.43354  2.70137
H 10.89166  2.51330  3.37565
H 10.94971  3.28359 -2.57485
C -8.88864  0.24627  3.66303
C -8.33701 -1.07457  3.13328
C -8.33523  1.44938  2.89420
H -8.51374  0.34384  4.68281
C -8.94154 -1.74982  2.06269
C -7.19292 -1.66106  3.69231
C -8.92502  1.95922  1.73311
C -7.19960  2.11880  3.36534
C -8.43943 -2.93836  1.52693
H -9.83661 -1.32285  1.62579
C -6.64397 -2.83503  3.18637
O -6.57748 -1.07348  4.80898
C -8.44732  3.10231  1.07888
H -9.78732  1.44741  1.32127
C -6.71123  3.27891  2.78430
O -6.55095  1.59524  4.48553
C -9.09440 -3.68173  0.35917
C -7.26412 -3.43771  2.10274
H -5.74635 -3.25557  3.62019
C -5.38213 -0.46869  4.53858
C -9.04190  3.60597 -0.23112
C -7.35425  3.76078  1.65287
H -5.84398  3.78262  3.18948
C -5.39384  0.92337  4.25008
H -8.81836 -4.73143  0.47018
C -8.50027 -3.21637 -0.96915
O -6.74423 -4.61832  1.57429
C -8.38149  2.88638 -1.40520
H -8.76324  4.65675 -0.31656
O -6.90493  4.95458  1.07256
C -8.95492 -2.05183 -1.60611
C -7.44418 -3.90357 -1.58224
C -5.61110 -4.57163  0.83124
C -3.18693 -0.50914  4.13636
C -8.86168  1.66710 -1.88923
C -7.25736  3.42544 -2.04593
C -5.66987  4.90360  0.50159
C -3.23547  0.81212  3.71118
C -8.36943 -1.53678 -2.76540
H -9.78951 -1.51740 -1.16726
C -6.80615 -3.41269 -2.71714
O -6.97734 -5.11795 -1.04823
C -5.72024 -4.99924 -0.52230
C -8.29792  0.99580 -2.97976
H -9.72302  1.22478 -1.40281
C -6.66254  2.80596 -3.13886
O -6.76470  4.65626 -1.60162
C -5.59547  4.68458 -0.90451
C -8.89343 -0.30712 -3.50592
C -7.25790 -2.22465 -3.26613
H -5.96339 -3.93763 -3.14715
C -3.41474 -4.33561  0.54865
C -7.19083  1.59945 -3.59041
H -5.79943  3.24510 -3.62320
C -3.44026  4.95116  0.60379
H -8.54150 -0.40443 -4.53378
O -6.60957 -1.69288 -4.38247
C -3.49925 -4.90412 -0.71354
O -6.59965  0.98977 -4.70891
C -3.36875  4.69148 -0.75729
C -5.44206 -1.03316 -4.15954
C -5.41680  0.36191 -4.44014
C -3.27064 -0.95415 -3.66079
C -3.22027  0.37581 -4.05351
C -10.42946 -0.27148 -3.54917
H -10.76863  0.58401 -4.13930
H -10.88340 -0.18936 -2.55771
C -10.62711 -3.59990  0.38651
H -11.04737 -4.19962 -0.42506
H -11.00305 -2.57974  0.26913
C -10.42318  0.26253  3.73403
H -10.78025 -0.57565  4.33845
H -10.89803  0.18739  2.75178
C -10.57461  3.52808 -0.26828
H -10.99692  4.10868  0.55618
H -10.95459  2.50675 -0.17884
H -10.94975  3.93742 -1.21010
H -10.76579  1.19384  4.19270
H -11.00789 -3.98405  1.33666
H -10.81101 -1.18647 -4.01005
C  2.05141  4.42978 -2.46272
C  1.91042  2.16154 -3.03178
C  1.92318 -4.20982  2.31414
C  1.91863 -1.93490  2.85487
C -1.98342 -3.97649  0.81313
C -2.10169 -5.04429 -1.25919
C -1.90095 -1.36563 -3.20207
C -1.80444  0.85666 -3.91116
C -1.76417 -0.98414  4.02557
C -1.87720  1.20056  3.20290
C -2.03670  5.04065  1.14530
C  1.85545  2.47122  3.56454
C  1.90064  3.81670  1.65829
C -1.92333  4.53716 -1.13606
C  1.90478 -2.45800 -3.53329
C  1.89805 -3.93413 -1.72648
N  1.14831 -3.11592 -2.55486
N  1.22196  3.31264 -2.67529
N -1.10125 -0.25891 -3.42710
N -1.27983 -4.47697 -0.26204
N  1.17546 -3.02143  2.43450
N  1.12039  3.12682  2.56603
N -1.07648  0.10360  3.45156
N -1.21611  4.78763  0.02815
O  1.46862  1.03460 -3.14607
O  1.68944  5.53633 -2.14639
O  1.53123  4.39646  0.65205
O -1.65282  5.27005  2.26615
O -1.47301  4.20305 -2.21701
O -1.34252  1.95296 -4.12680
O -1.56979 -2.43881 -2.72658
O -1.72142 -5.53416 -2.29335
O  1.47811 -1.68191 -4.35722
O  1.55223 -0.78100  2.98957
O  1.48542 -5.29494  2.02134
O  1.48987 -4.58652 -0.78299
O -1.27220 -2.03693  4.35232
O -1.54992 -3.34631  1.76461
O -1.55979  2.24924  2.66387
O  1.39947  1.78008  4.44439
H  0.20413  3.40531 -2.62861
H -0.19303  4.73302  0.11168
H  0.11101  2.96396  2.48063
H -0.07249  0.03664  3.25669
H  0.16202 -3.01540  2.26757
H -0.25270 -4.48545 -0.34088
H  0.13277 -2.96331 -2.48565
H -0.09488 -0.21453 -3.27514
N  4.38431  2.48183  3.94463
N  4.42257  4.26250  1.71829
```

N 4.38389 1.90057 -3.63338
N 4.60869 4.54933 -2.59925
N 4.42418 -2.54840 -3.92702
N 4.41290 -4.39407 -1.75116
N 4.36600 -1.83400 3.56182
N 4.46351 -4.50587 2.56707
N -4.46413 -4.17839 1.36044
N -4.66515 -5.21231 -1.29161
N -4.26980 -1.18802 4.52584
N -4.33355 1.56475 3.78237
N -4.37948 -1.69256 -3.71969
N -4.29821 1.07037 -4.42070
N -4.59640 5.05298 1.26328
N -4.44182 4.55685 -1.54168
C 4.68097 1.36056 -0.15400
C 4.99860 0.03849 0.54411
C -0.76832 1.33617 -0.47355
H 4.94333 0.15472 1.63236
H -0.62312 1.70449 -1.49249
H -0.69555 2.20838 0.17944
C -2.14586 0.72843 -0.35968
C -2.44441 -0.36552 0.46032
C -3.20022 1.35852 -1.03120
C -3.76357 -0.78336 0.63578
H -1.64896 -0.90095 0.96229
C -4.51611 0.93724 -0.85708
H -2.99306 2.18212 -1.70492
C -4.82178 -0.13348 -0.01364
H -3.97165 -1.62012 1.29604
H -5.31491 1.44797 -1.38352
C -6.24115 -0.59405 0.16219
H -6.43614 -1.49477 -0.42731
H -6.45003 -0.83698 1.20403
H -6.95394 0.16967 -0.15852
H 3.67792 1.70541 0.09520
H 4.74410 1.21568 -1.23326
C 6.36264 -0.51456 0.14458
H 6.41629 -0.64221 -0.93914
H 7.14481 0.18166 0.45148
H 6.54818 -1.47586 0.62014
H 5.41821 2.10991 0.13719
N 3.98401 -0.91950 0.20056
C 3.08001 -1.59127 -0.10583
H 1.23617 -1.20987 -0.40349
C 0.45261 0.50289 -0.16938
O 0.36816 -0.76854 -0.58477
O 1.44690 0.97334 0.35524

(*Z*)-**4b-TS@1**$_2$

C 9.00101 -2.73805 2.46779
C 8.38675 -1.40346 2.90230
C 8.42899 -3.19552 1.12779
H 8.67893 -3.47427 3.20571
C 8.94270 -0.15492 2.60464
C 7.20458 -1.40093 3.65267
C 8.93968 -2.70328 -0.08197
C 7.34982 -4.08756 1.04446
C 8.38212 1.05102 3.04979
H 9.84639 -0.11581 2.00755
C 6.62960 -0.24288 4.15485
O 6.63312 -2.63712 3.94116
C 8.40360 -3.02431 -1.33144
H 9.78293 -2.02345 -0.04349
C 6.76656 -4.42103 -0.17466
O 6.80958 -4.67285 2.20355
C 8.96064 2.41418 2.68599
C 7.23812 0.96898 3.85273
H 5.73157 -0.28003 4.75661
C 5.49941 -2.99910 3.29133
C 8.98620 -2.49941 -2.64506
C 7.28468 -3.86737 -1.33510
H 5.91209 -5.08439 -0.21173
C 5.56664 -4.18416 2.50773
H 8.62543 3.10490 3.46051
C 8.37503 2.93323 1.37281
O 6.70432 2.14773 4.39053
C 8.39488 -1.14073 -3.01549
H 8.65959 -3.19430 -3.41974
O 6.69149 -4.17364 -2.56333
C 8.90650 2.57845 0.13048
C 7.28740 3.81760 1.36129
C 5.50284 2.52306 3.86719
C 3.32162 -2.85853 2.85219
C 8.94056 0.06377 -2.55086
C 7.26845 -1.04831 -3.84278
C 5.51794 -3.55024 -2.85342
C 3.35123 -4.10552 2.24317
C 8.43381 3.09086 -1.08368
H 9.72816 1.87184 0.10478
C 6.80803 4.38932 0.19064
O 6.67617 4.14715 2.57608
C 5.49859 3.51688 2.84957
C 8.39567 1.31358 -2.85781
H 9.83079 0.02289 -1.93427
C 6.66122 0.16720 -4.14255
O 6.73979 -2.21726 -4.41092
C 5.53119 -2.58424 -3.89818
C 9.02076 2.63562 -2.41467
C 7.39310 4.02559 -1.01866
H 5.97623 5.08172 0.21442
C 3.28943 2.31871 3.66141
C 7.22527 1.32441 -3.62786
H 5.77538 0.20583 -4.76263
C 3.32363 -3.23170 -2.62498
H 8.71904 3.37867 -3.15400
O 6.89918 4.61099 -2.19619
C 3.29941 3.21451 2.60020
O 6.67613 2.56671 -3.94280
C 3.31993 -2.36008 -3.70558
C 5.64855 4.15898 -2.52157
C 5.54629 2.98227 -3.31601
C 3.42923 4.16817 -2.28385
C 3.35201 2.93443 -2.91517
C 10.55584 2.59372 -2.40071
H 10.93308 2.31042 -3.38698
H 10.95855 1.88057 -1.67596
C 10.49662 2.43230 2.68280
H 10.85869 3.44364 2.48032
H 10.93118 1.77129 1.92767
C 10.53620 -2.71184 2.47879
H 10.90013 -2.43551 3.47201
H 10.95726 -1.99952 1.76365
C 10.52216 -2.49934 -2.64159
H 10.89573 -3.50626 -2.43774
H 10.94873 -1.83245 -1.88702
H 10.89935 -2.17861 -3.61626
H 10.92854 -3.70055 2.22704
H 10.87663 2.11517 3.65764
H 10.95326 3.57974 -2.14627
C -8.94301 -0.21506 3.62716
C -8.37755 -1.46606 2.96001
C -8.37203 1.06556 3.01188
H -8.58940 -0.23623 4.65898
C -8.94821 -2.00329 1.79670
C -7.24867 -2.11832 3.47523
C -8.95033 1.71985 1.91928
C -7.22847 1.65739 3.56165
C -8.42547 -3.11446 1.12949
H -9.83043 -1.52232 1.39066
C -6.68006 -3.21714 2.84041
O -6.66470 -1.67971 4.67502
C -8.45417 2.92571 1.40685
H -9.81955 1.27602 1.44716
C -6.72098 2.87079 3.12353
O -6.60200 1.00164 4.62212
C -9.04978 -3.70518 -0.13795
C -7.26467 -3.68062 1.67185
H -5.79441 -3.68998 3.24399
C -5.45399 -1.06692 4.50956
C -9.03974 3.59556 0.16904
C -7.35365 3.49423 2.05741
H -5.84883 3.31038 3.58855
C -5.44547 0.34560 4.34679
H -8.75122 -4.75413 -0.16430
C -8.45399 -3.05755 -1.38673
O -6.71388 -4.78287 1.01766
C -8.38690 3.02910 -1.09055
H -8.75047 4.64583 0.21900
O -6.88608 4.74368 1.62696
C -8.94113 -1.84223 -1.89113
C -7.36335 -3.63039 -2.05463
C -5.56086 -4.60862 0.32232
C -3.24682 -1.09442 4.17976
C -8.87595 1.88528 -1.72668
C -7.26306 3.64037 -1.66339
C -5.65076 4.73873 1.05438
C -3.27363 0.25805 3.86481
C -8.35950 -1.17641 -2.97368
H -9.80032 -1.39278 -1.40631
C -6.73079 -2.99250 -3.11618
O -6.86588 -4.88252 -1.65453
C -5.62551 -4.80026 -1.08550
C -8.32386 1.35990 -2.90055
H -9.73156 1.38131 -1.29307
C -6.68618 3.17289 -2.83745
O -6.74792 4.79127 -1.05891
C -5.57873 4.69651 -0.36749
C -8.91061 0.11935 -3.56923
C -7.22162 -1.76742 -3.53520
H -5.86451 -3.43531 -3.58955
C -3.36574 -4.27360 0.14820
C -7.22688 2.04207 -3.44327
H -5.82612 3.67049 -3.26766
C -3.42196 4.70725 1.16051
H -8.56617 0.14433 -4.60397
O -6.59396 -1.09811 -4.58653
C -3.40596 -4.59665 -1.20072
O -6.65025 1.59534 -4.64366
C -3.35422 4.61094 -0.22221
C -5.43835 -0.43985 -4.31089
C -5.44541 0.97367 -4.47082
C -3.26632 -0.35425 -3.82653
C -3.24267 1.00178 -4.12303
C -10.44687 0.13956 -3.59553
H -10.80152 1.04938 -4.08702
H -10.89176 0.10942 -2.59715
C -10.58423 -3.65953 -0.11761
H -10.98486 -4.14975 -1.00881
H -10.98074 -2.64081 -0.09678
C -10.47856 -0.20654 3.66659
H -10.84570 -1.10505 4.16974
H -10.93181 -0.17732 2.67186
C -10.57318 3.53826 0.12014
H -10.99255 4.00889 1.01340
H -10.96250 2.51763 0.07290
H -10.94177 4.07179 -0.76004
H -10.83409 0.66993 4.21484
H -10.96435 -4.17875 0.76622
H -10.82276 -0.72509 -4.14902
C 2.03404 4.59818 -1.91653
C 1.93105 2.44804 -2.83293
C 1.93913 -4.47085 1.86109
C 1.92795 -2.31030 2.75034
C -1.95447 -3.92228 0.51035
C -1.99440 -4.57080 -1.73043
C -1.89128 -0.77043 -3.39456
C -1.83105 1.49340 -3.96483
C -1.82272 -1.57025 4.08045
C -1.89817 0.67465 3.43273
C -2.01834 4.68687 1.70841
C 1.86724 1.89450 3.90897
C 1.89720 3.32893 2.06611
C -1.91322 4.45598 -0.61483

```
C  1.90548 -1.89281 -3.92998
C  1.92949 -3.30533 -2.07636
N  1.16706 -2.51383 -2.90754
N  1.22386  3.51199 -2.30025
N -1.11199  0.36273 -3.54049
N -1.21900 -4.15550 -0.62916
N  1.18244 -3.32587  2.18923
N  1.12851  2.55588  2.91429
N -1.11201 -0.44524  3.61264
N -1.20350  4.53988  0.56871
O  1.51621  1.34219 -3.12953
O  1.65225  5.63152 -1.42505
O  1.52540  3.96609  1.09581
O -1.62935  4.77347  2.84746
O -1.46780  4.24297 -1.72916
O -1.37267  2.59806 -4.13784
O -1.53987 -1.86695 -2.99498
O -1.57279 -4.85262 -2.82478
O  1.46148 -1.15255 -4.77427
O  1.56012 -1.18694  3.05296
O  1.50641 -5.49712  1.39980
O  1.55848 -3.90462 -1.07917
O -1.34247 -2.64408  4.34843
O -1.55322 -3.50383  1.58570
O -1.55726  1.76384  2.99735
O  1.41662  1.17322  4.76608
H  0.20427  3.57965 -2.22054
H -0.18464  4.43085  0.64095
H  0.11268  2.41468  2.84443
H -0.10569 -0.50533  3.42593
H  0.16660 -3.28497  2.01891
H -0.20131 -4.02766 -0.69681
H  0.15686 -2.33754 -2.79263
H -0.10627  0.43080 -3.37296
N  4.39630  1.93697  4.30093
N  4.40063  3.84412  2.18292
N  4.40583  2.32647 -3.46882
N  4.58862  4.78551 -2.03806
N  4.43092 -2.00228 -4.35224
N  4.41758 -3.85466 -2.18167
N  4.39093 -2.28585  3.41061
N  4.48790 -4.77478  2.01942
N -4.43621 -4.29606  0.94577
N -4.54720 -4.83896 -1.85309
N -4.34736 -1.79367  4.47381
N -4.36719  1.01442  3.96901
N -4.35911 -1.11025 -3.93580
N -4.34044  1.70157 -4.41711
N -4.57599  4.76250  1.82863
N -4.42792  4.61056 -1.01694
C  4.63289  1.36782 -0.13978
C  4.97331  0.03056  0.51367
C -0.71840  1.14084 -0.21142
H  4.99596  0.11907  1.60656
H -0.52986  1.72608 -1.11491
H -0.58421  1.84043  0.61899
C -2.13597  0.62808 -0.21399
C -2.53575 -0.49028  0.52886
C -3.12332  1.35529 -0.88764
C -3.88171 -0.83698  0.62586
H -1.78895 -1.09366  1.02568
C -4.47001  1.00569 -0.79169
H -2.84378  2.21410 -1.48690
C -4.87333 -0.08900 -0.02459
H -4.16713 -1.69846  1.22362
H -5.21473  1.59823 -1.31309
C -6.32276 -0.46891  0.08685
H -6.54950 -1.34190 -0.53144
H -6.58465 -0.72462  1.11475
H -6.97904  0.34255 -0.23595
H  3.65644  1.73360  0.17464
H  4.62010  1.23722 -1.22308
C  6.29514 -0.53944  0.01555
H  6.28066 -0.64357 -1.07241
H  7.10930  0.13724  0.28164
H  6.49494 -1.51375  0.45793
H  5.40098  2.10020  0.11305
N  3.90163 -0.92050  0.24551
C  2.75310 -1.09432  0.06424
H  1.63957 -1.45299 -0.13887
C  0.43276  0.15287 -0.11248
O  0.25204 -1.08720 -0.32190
O  1.57010  0.65325  0.15844
```

(*Z*)-4b@1_2

```
C  8.98946 -2.61669 -2.36987
C  8.38146 -3.02240 -1.02805
C  8.40423 -1.29785 -2.86280
H  8.67714 -3.37675 -3.08681
C  8.89266 -2.56837  0.19018
C  7.28484 -3.89209 -0.96648
C  8.95049 -0.05591 -2.52024
C  7.27654 -1.29123 -3.69146
C  8.38874 -2.96953  1.43322
H  9.72192 -1.87057  0.17323
C  6.75934 -4.33870  0.23717
O  6.73337 -4.36327 -2.16425
C  8.41036  1.15579 -2.96005
H  9.83554 -0.03401 -1.89539
C  6.67745 -0.11565 -4.12785
O  6.76806 -2.52015 -4.12964
C  8.96654 -2.42610  2.73621
C  7.32755 -3.88333  1.42222
H  5.91961 -5.02175  0.25364
C  5.54416 -3.82859 -2.55417
C  9.03162  2.51065 -2.62503
C  7.24955  1.08803 -3.74076
H  5.79375 -0.13948 -4.75141
C  5.56068 -2.88460 -3.62138
H  8.64190 -3.10865  3.52279
C  8.36663 -1.06308  3.08115
O  6.79756 -4.36383  2.63509
C  8.44249  3.05165 -1.32797
H  8.72037  3.19527 -3.41480
O  6.70299  2.29087 -4.19419
C  8.91830  0.15120  2.66522
C  7.19882 -0.99360  3.85038
C  5.55902 -3.84690  2.89407
C  3.32211 -3.66462 -2.48079
C  8.93052  2.65308 -0.07642
C  7.36728  3.94765 -1.34140
C  5.55677  2.74455 -3.62602
C  3.33558 -2.77489 -3.54618
C  8.37458  1.39527  3.01546
H  9.80139  0.13238  2.03728
C  6.64848  0.20601  4.27037
O  6.60630 -2.19707  4.23105
C  5.48350 -2.60420  3.58600
C  8.37472  3.08246  1.13169
H  9.77772  1.97764 -0.04625
C  6.75463  4.37671 -0.16892
O  6.87925  4.43430 -2.56566
C  5.63822  3.95533 -2.88158
C  8.95494  2.70773  2.49815
C  7.25620  1.38603  3.85749
H  5.76102  0.22628  4.88850
C  3.35181 -3.78574  2.58799
C  7.25239  3.91567  1.04049
H  5.90041  5.04009 -0.20137
C  3.35525  2.70186 -3.26729
H  8.62015  3.48207  3.18969
O  6.71437  2.60392  4.30701
C  3.31463 -2.49388  3.09416
O  6.63600  4.31690  2.22973
C  3.41608  3.95569 -2.67375
C  5.50292  2.88586  3.74828
C  5.48380  3.68416  2.56926
C  3.29920  2.59013  3.56471
C  3.30974  3.24705  2.34140
C 10.49099  2.71996  2.49980
H 10.85897  3.70050  2.18640
H 10.92541  1.97554  1.82675
C 10.50244 -2.41301  2.73982
H 10.87372 -2.07221  3.71012
H 10.92629 -1.75616  1.97506
C 10.52508 -2.61458 -2.33926
H 10.89499 -3.60061 -2.04605
H 10.93875 -1.88676 -1.63563
C 10.56669  2.47989 -2.61694
H 10.94081  2.13051 -3.58301
H 10.97886  1.82209 -1.84669
H 10.95844  3.48411 -2.43453
H 10.91913 -2.37541 -3.33072
H 10.88429 -3.42049  2.55497
H 10.86527  2.51251  3.50585
C -9.06750 -3.57088  0.18099
C -8.46791 -3.03833 -1.11766
C -8.43188 -2.88407  1.39119
H -8.78357 -4.62247  0.24263
C -8.93799 -1.85944 -1.71385
C -7.39996 -3.68920 -1.74906
C -8.94702 -1.72305  1.97204
C -7.27361 -3.41634  1.97244
C -8.36766 -1.30532 -2.86242
H -9.77647 -1.34859 -1.25459
C -6.77830 -3.16226 -2.87616
O -6.92520 -4.91333 -1.24940
C -8.38018 -1.11118  3.09938
H -9.82554 -1.26889  1.52867
C -6.69372 -2.87840  3.11088
O -6.73813 -4.57089  1.40457
C -8.90595 -0.05056 -3.54937
C -7.25767 -1.97063 -3.39592
H -5.93267 -3.66493 -3.32701
C -5.67879 -4.81839 -0.69665
C -8.94134  0.18859  3.67012
C -7.26368 -1.73843  3.66845
H -5.81697 -3.33125  3.55521
C -5.59044 -4.48807  0.68490
H -8.56558 -0.10425 -4.58420
C -8.29937  1.22462 -2.97077
O -6.64202 -1.41226 -4.51827
C -8.35147  1.41053  2.96094
H -8.59565  0.24230  4.70341
O -6.69246 -1.22952  4.84765
C -8.85050  1.85415 -1.84845
C -7.17658  1.83066 -3.55004
C -5.45496 -0.78195 -4.32012
C -3.45412 -4.73740 -0.83956
C -8.91372  1.98457  1.81643
C -7.19607  2.02009  3.46442
C -5.47196 -0.64899  4.65121
C -3.38352 -4.26970  0.46514
C -8.33641  3.03103 -1.29736
H -9.72282  1.40792 -1.38557
C -6.61683  2.99568 -3.03432
O -6.60959  1.27510 -4.70727
C -5.42346  0.62793 -4.51328
C -8.39041  3.13169  1.20490
H -9.79125  1.51977  1.38170
C -6.66000  3.17952  2.92672
O -6.58894  1.43811  4.57860
C -5.44159  0.74528  4.36909
C -8.97046  3.70865 -0.08296
C -7.19781  3.57047 -1.91054
H -5.74383  3.43952 -3.49601
C -3.26218 -0.75940 -3.92711
C -7.27629  3.72602  1.80917
H -5.77924  3.63805  3.35569
C -3.26659 -0.74152  4.32547
H -8.66268  4.75427 -0.11950
O -6.66832  4.75751 -1.39575
C -3.21080  0.59445 -4.22587
```

```
O -6.77509  4.92476  1.28286
C -3.27339  0.57879  3.89529
C -5.49377  4.68803 -0.71276
C -5.54613  4.83778  0.70219
C -3.27464  4.52900 -0.58580
C -3.31839  4.73059  0.78649
C -10.50498  3.67614 -0.11900
H -10.91010  4.22424  0.73575
H -10.91309  2.66252 -0.08282
C -10.44193 -0.01483 -3.57202
H -10.78901  0.86355 -4.12264
H -10.88383  0.02613 -2.57266
C -10.60136 -3.50408  0.19919
H -11.00936 -4.06665 -0.64461
H -10.98584 -2.48289  0.12935
C -10.47681  0.21330  3.69647
H -10.85588 -0.64211  4.26193
H -10.92287  0.17285  2.69897
H -10.82792  1.13096  4.17575
H -10.98597 -3.93838  1.12586
H -10.82866 -0.91060 -4.06510
H -10.86923  4.14464 -1.03715
C  1.88415  2.18129  3.86898
C  1.92434  3.20258  1.76801
C  1.91738 -2.37620 -3.84810
C  1.89930 -3.83913 -2.03319
C -1.88097 -1.22472 -3.57038
C -1.77871  1.04370 -4.13070
C -1.85085  4.28671 -0.98580
C -1.90668  4.69269  1.31495
C -2.05020 -4.85511 -1.37223
C -1.94757 -3.96336  0.78083
C -1.85524 -1.25623  4.24056
C  1.95523 -4.17927  2.19672
C  1.92568 -1.95741  2.91606
C -1.90070  0.92494  3.40526
C  2.02019  4.36872 -2.29431
C  1.94689  2.18752 -3.14040
N  1.22677  3.25634 -2.62989
N  1.15298  2.59529  2.73722
N -1.12002  4.40995  0.17989
N -1.07467 -0.10776 -3.73295
N  1.14688 -3.08310 -2.91353
N  1.18883 -3.01393  2.42275
N -1.23776 -4.39270 -0.31763
N -1.12802 -0.18393  3.68150
O  1.55748  3.59838  0.67171
O  1.42392  1.63966  4.84384
O  1.54316 -0.81223  3.11323
O -1.39473 -2.32321  4.56402
O -1.55026  1.95370  2.84663
O -1.49161  4.86444  2.43453
O -1.43663  3.98748 -2.09250
O -1.30196  2.13511 -4.33368
O  1.62525  5.40326 -1.81303
O  1.49689 -4.46121 -1.06491
O  1.49008 -1.60861 -4.68018
O  1.54410  1.05920 -3.35567
O -1.65983 -5.25793 -2.44011
O -1.54867 -2.33724 -3.20549
O -1.51788 -3.42324  1.78951
O  1.52904 -5.22971  1.78574
H  0.13278  2.46341  2.69020
H -0.12387 -0.27363  3.48070
H  0.17588 -3.01783  2.24150
H -0.21733 -4.34462 -0.42317
H  0.12849 -2.95506 -2.86809
H -0.05669 -0.09997 -3.67510
H  0.20633  3.30805 -2.54992
H -0.10901  4.24994  0.24175
N  4.48653 -4.47359  2.43970
N  4.37308 -1.88434  3.63342
N  4.39650  3.82723  1.82693
N  4.40548  2.36839  4.27854
N  4.56660  4.59226 -2.43788
N  4.42107  2.08132 -3.77934
N  4.42287 -4.21291 -1.96287
N  4.45814 -2.35734 -4.13276
N -4.38209 -1.48106 -3.98165
N -4.29630  1.32378 -4.49540
N -4.61108 -4.98728 -1.46004
N -4.44764 -4.15598  1.26521
N -4.35673  4.51106 -1.36703
N -4.46067  4.87987  1.46121
N -4.37813 -1.39476  4.67761
N -4.35365  1.36014  3.93308
C  0.33294 -0.48311 -0.29917
O -0.03286 -1.60530 -0.55587
C -0.53813  0.74842 -0.26171
H -0.27712  1.33843 -1.14685
H -0.23174  1.35344  0.59742
C -2.02007  0.45468 -0.21793
C -2.53815 -0.51838  0.64762
C -2.91886  1.19049 -0.99255
C -3.90967 -0.72511  0.75389
H -1.86803 -1.13045  1.23547
C -4.29663  0.98564 -0.88103
H -2.55137  1.93582 -1.68720
C -4.81618  0.03200 -0.00310
H -4.28040 -1.48851  1.43249
H -4.97254  1.57964 -1.48691
C -6.29758 -0.19724  0.12190
H -6.58228 -1.17127 -0.28666
H -6.61169 -0.18510  1.16841
H -6.87150  0.56477 -0.40842
C  5.99341 -0.22606  0.50244
C  4.50105  0.10336  0.46219
N  3.78830 -1.14533  0.16043
C  2.55183 -1.23473 -0.03932
O  1.64573 -0.17122 -0.01630
H  4.17628  0.42631  1.46268
H  2.07095 -2.18818 -0.21595
H  6.33997 -0.53329 -0.48995
H  6.18937 -1.04924  1.18924
H  6.58139  0.63945  0.81790
C  4.22690  1.21562 -0.55397
H  3.19767  1.57072 -0.50923
H  4.89111  2.06474 -0.37043
H  4.42476  0.83353 -1.55838
```

(*E*)-4b-TS@1_2

```
C  8.99185 -2.24529 -2.81733
C  8.41732 -2.88982 -1.55464
C  8.40010 -0.85995 -3.07083
H  8.66485 -2.87005 -3.64939
C  8.95430 -2.66733 -0.28480
C  7.31302 -3.74916 -1.62467
C  8.94807  0.30720 -2.52154
C  7.27736 -0.70586 -3.89345
C  8.45650 -3.26511  0.88035
H  9.78464 -1.97697 -0.19152
C  6.81369 -4.41135 -0.51323
O  6.70624 -3.95020 -2.86937
C  8.40952  1.57816 -2.74554
H  9.83998  0.22138 -1.91175
C  6.67718  0.52893 -4.11450
O  6.74887 -1.83522 -4.53784
C  9.02375 -2.90338  2.25039
C  7.39246 -4.16449  0.72819
H  5.96506 -5.07728 -0.60186
C  5.53339 -3.29882 -3.09597
C  9.04253  2.86558 -2.21680
C  7.24396  1.64590 -3.51971
H  5.79270  0.61318 -4.73178
C  5.54418 -2.24178 -4.04963
H  8.70321 -3.69149  2.93320
C  8.40584 -1.60604  2.77945
O  6.84927 -4.82704  1.84569
C  8.47006  3.22569 -0.85090
H  8.73513  3.65907 -2.89906
O  6.69433  2.90848 -3.74863
C  8.95422 -0.33651  2.56812
C  7.23065 -1.66386  3.53846
C  5.60638 -4.35751  2.17754
C  3.33752 -3.00309 -2.83819
C  8.94326  2.61342  0.31622
C  7.42448  4.14685 -0.71061
C  5.56676  3.26907 -3.08416
C  3.33391 -2.03578 -3.83389
C  8.39824  0.83225  3.10991
H  9.85287 -0.25061  1.96807
C  6.66480 -0.54950  4.13763
O  6.65724 -2.91838  3.73355
C  5.52946 -3.23403  3.04903
C  8.39970  2.85043  1.58093
H  9.76481  1.91166  0.23095
C  6.83349  4.40183  0.52300
O  6.91737  4.81124 -1.84108
C  5.66835  4.37402 -2.19225
C  8.97640  2.22029  2.85014
C  7.27027  0.68313  3.92530
H  5.77319 -0.63423  4.74415
C  3.39166 -4.26450  1.90640
C  7.30384  3.72317  1.63753
H  5.99114  5.07680  0.60126
C  3.37725  3.16227 -2.66552
H  8.64026  2.84781  3.67646
O  6.73571  1.81460  4.56244
C  3.35238 -3.06648  2.60715
O  6.66905  3.91646  2.86973
C  3.45102  4.33906 -1.93138
C  5.52859  2.20311  4.06301
C  5.51147  3.23124  3.07886
C  3.32455  1.95009  3.82488
C  3.32910  2.86105  2.77684
C 10.51278  2.23580  2.85450
H 10.87734  3.25901  2.73172
H 10.94895  1.63391  2.05239
C 10.55910 -2.87356  2.26637
H 10.92049 -2.66220  3.27639
H 10.97924 -2.11465  1.60045
C 10.52822 -2.24309 -2.81840
H 10.90373 -3.26348 -2.70533
H 10.95586 -1.64491 -2.00887
C 10.57763  2.81925 -2.22286
H 10.94296  2.60067 -3.22988
H 10.98612  2.05873 -1.55160
H 10.98121  3.78531 -1.90831
H 10.90185 -1.83636 -3.76189
H 10.95471 -3.84243  1.95064
H 10.88817  1.84331  3.80334
C -9.06535 -3.63064 -0.41054
C -8.47319 -2.88548 -1.60633
C -8.44411 -3.14322  0.90244
H -8.76261 -4.67300 -0.52020
C -8.96830 -1.63987 -2.02178
C -7.37710 -3.39993 -2.31230
C -8.97084 -2.09284  1.65933
C -7.28358 -3.75095  1.39951
C -8.38892 -0.89319 -3.05195
H -9.82972 -1.23168 -1.50581
C -6.74747 -2.68302 -3.32385
O -6.87606 -4.67622 -2.00743
C -8.40485 -1.65534  2.86631
H -9.84893 -1.57716  1.28897
C -6.70755 -3.39092  2.60791
O -6.72603 -4.79334  0.66053
C -8.94167  0.44365 -3.54783
C -7.24719 -1.43480 -3.65447
H -5.87974 -3.08641 -3.82887
C -5.63586 -4.63931 -1.43379
C -8.96052 -0.45321  3.62583
C -7.28165 -2.35233  3.33224
```

```
H -5.82597 -3.89953  2.97542
C -5.57075 -4.56274 -0.01571
H -8.60301  0.54495 -4.57976
C -8.34477  1.62709 -2.78984
O -6.63161 -0.69081 -4.66139
C -8.37306  0.86303  3.10712
H -8.60914 -0.55466  4.65360
O -6.70560 -2.01850  4.56944
C -8.88382  2.05882 -1.57199
C -7.24587  2.34268 -3.28416
C -5.46711 -0.05865 -4.36643
C -3.41511 -4.44832 -1.53062
C -8.94463  1.60493  2.06781
C -7.21748  1.39456  3.69310
C -5.48315 -1.41751  4.46068
C -3.37153 -4.23612 -0.15942
C -8.37738  3.13948 -0.84490
H -9.73980  1.52731 -1.17373
C -6.69007  3.41380 -2.58998
O -6.68236  1.99537 -4.52272
C -5.47450  1.36326 -4.42234
C -8.43299  2.83849  1.64340
H -9.82034  1.20877  1.56673
C -6.69522  2.63016  3.34461
O -6.59783  0.65601  4.70192
C -5.44973  0.00285  4.38854
C -9.01485  3.60900  0.46265
C -7.25070  3.78363 -1.37431
H -5.82849  3.93766 -2.98426
C -3.28156 -0.01120 -3.93917
C -7.32345  3.34138  2.33204
H -5.81632  3.02235  3.83838
C -3.27430 -1.46318  4.14623
H -8.71311  4.64877  0.59376
O -6.71178  4.86861 -0.67554
C -3.26171  1.36283 -4.13769
O -6.83809  4.61431  1.99874
C -3.27549 -0.09349  3.91573
C -5.54277  4.68651 -0.00250
C -5.60617  4.62629  1.41930
C -3.32318  4.50789  0.12044
C -3.37907  4.51547  1.50691
C -10.54919  3.57337  0.41828
H -10.95866  3.97814  1.34765
H -10.95105  2.56391  0.29422
C -10.47766  0.47087 -3.56260
H -10.83186  1.41473 -3.98544
H -10.91665  0.37109 -2.56616
C -10.59990 -3.59238 -0.38606
H -10.99942 -4.01146 -1.31333
H -11.00028 -2.57992 -0.28378
C -10.49571 -0.43190  3.66276
H -10.87265 -1.35935  4.10206
H -10.94605 -0.33049  2.67140
H -10.84464  0.40805  4.26928
H -10.97745 -4.18166  0.45382
H -10.86038 -0.35055 -4.17414
H -10.91545  4.17761 -0.41594
C  1.91196  1.48317  4.04254
C  1.93680  2.93953  2.21550
C  1.91937 -1.55949 -4.02642
C  1.93728 -3.14485 -2.31625
C -1.89766 -0.45942 -3.56812
C -1.84361  1.84138 -3.98765
C -1.89331  4.32601 -0.29250
C -1.97365  4.41067  2.04095
C -2.00307 -4.40705 -2.05687
C -1.95053 -3.95016  0.22942
C -1.86063 -1.96215  4.01491
C  1.98277 -4.62171  1.50866
C  1.94676 -2.53807  2.56457
C -1.89268  0.31829  3.50121
C  2.05795  4.72310 -1.51192
C  1.95874  2.66038 -2.61144
N  1.25266  3.66736 -1.97856
N  1.17575  2.12262  3.02798
N -1.17229  4.30184  0.88525
N -1.11852  0.68324 -3.65011
N  1.17631 -2.27794 -3.07502
N  1.21121 -3.51810  1.93408
N -1.21847 -4.12348 -0.92264
N -1.12721 -0.82396  3.61485
O  1.55749  3.58311  1.25022
O  1.45968  0.75204  4.88941
O  1.55299 -1.45254  2.96368
O -1.40294 -3.06013  4.21191
O -1.53116  1.41961  3.11348
O -1.57214  4.41703  3.17815
O -1.46314  4.17342 -1.42408
O -1.38471  2.95164 -4.11017
O  1.67066  5.70543 -0.92765
O  1.55871 -3.85451 -1.39902
O  1.47636 -0.74782 -4.80413
O  1.53384  1.58425 -2.99379
O -1.58918 -4.58634 -3.17583
O -1.54203 -1.57801 -3.24303
O -1.53890 -3.62137  1.33209
O  1.55937 -5.61578  0.97472
H  0.15756  1.99094  2.96435
H -0.12166 -0.88897  3.41787
H  0.19510 -3.47285  1.76645
H -0.20164 -3.98635 -0.98777
H  0.16230 -2.13506 -2.97785
H -0.10649  0.72603 -3.51682
H  0.23188  3.70986 -1.87834
H -0.15696  4.15532  0.93480
N  4.53144 -4.91254  1.64145
N  4.41618 -2.53831  3.21852
N  4.41702  3.53654  2.39619
N  4.43197  1.58242  4.47095
N  4.60667  4.94653 -1.64814
N  4.42958  2.61956 -3.28368
N  4.43254 -3.66402 -2.45490
N  4.44361 -1.62005 -4.44605
N -4.38023 -0.75587 -4.07204
N -4.36637  2.08398 -4.34612
N -4.55740 -4.62372 -2.20158
N -4.44429 -4.31122  0.63273
N -4.39931  4.60329 -0.66480
N -4.52727  4.56245  2.18557
N -4.38936 -2.15993  4.38720
N -4.35739  0.67472  4.05964
C  0.20604 -0.17446 -0.20230
O -0.03546 -1.33721 -0.45239
C -0.79407  0.95754 -0.18411
H -0.56363  1.58721 -1.04855
H -0.57119  1.57178  0.69382
C -2.24466  0.55179 -0.18517
C -2.70360 -0.54581  0.55510
C -3.18536  1.33447 -0.85926
C -4.06354 -0.82607  0.64135
H -1.99577 -1.19048  1.05768
C -4.54850  1.05265 -0.76978
H -2.85885  2.18312 -1.44857
C -5.00984 -0.02553 -0.01273
H -4.39590 -1.67923  1.22663
H -5.25915  1.68518 -1.28997
C -6.47269 -0.34359  0.08384
H -6.73054 -1.18707 -0.56116
H -6.74709 -0.62157  1.10181
H -7.09276  0.50324 -0.21521
C  5.99344 -0.94898  0.10064
C  5.00066  0.12335  0.55608
N  3.65692 -0.28398  0.38245
C  2.53057 -0.65038  0.11295
O  1.44460  0.29762  0.08915
H  5.13523  0.29148  1.63375
H  2.15242 -1.64906 -0.11093
H  5.89581 -1.11151 -0.97677
H  5.80396 -1.89599  0.60402
H  7.01982 -0.63960  0.30553
C  5.26091  1.42768 -0.20127
H  4.59109  2.22509  0.12435
H  6.29435  1.74203 -0.05736
H  5.10451  1.24665 -1.26608
```

(*E*)-**4b@1**$_2$

```
C  8.96594 -2.68392 -2.52074
C  8.35860 -3.13902 -1.19395
C  8.39896 -1.33407 -2.94706
H  8.63765 -3.40550 -3.26949
C  8.86149 -2.70556  0.03463
C  7.27215 -4.02315 -1.14993
C  8.96419 -0.11222 -2.55550
C  7.25963 -1.28214 -3.75699
C  8.36158 -3.13498  1.27046
H  9.68080 -1.99591  0.03256
C  6.76352 -4.51290  0.04498
O  6.67723 -4.42174 -2.35510
C  8.41563  1.12327 -2.91386
H  9.86915 -0.12762 -1.95941
C  6.65075 -0.08386 -4.10827
O  6.73288 -2.48717 -4.24779
C  8.94292 -2.59756  2.57386
C  7.31510 -4.06712  1.24276
H  5.92586 -5.19841  0.04796
C  5.50699 -3.79965 -2.67255
C  9.05261  2.47166 -2.56578
C  7.22834  1.09274 -3.65639
H  5.75164 -0.06985 -4.70922
C  5.52789 -2.83750 -3.72290
H  8.61692 -3.28139  3.35882
C  8.35091 -1.23260  2.92747
O  6.76741 -4.56424  2.44030
C  8.49124  3.03537 -1.26373
H  8.74655  3.16137 -3.35394
O  6.66444  2.31686 -4.00628
C  8.92753 -0.01117  2.56475
C  7.17537 -1.17649  3.68522
C  5.53065 -4.03838  2.70385
C  3.31372 -3.45061 -2.43927
C  8.97387  2.60622 -0.02063
C  7.46103  3.98416 -1.24912
C  5.56057  2.76003 -3.35472
C  3.31949 -2.58281 -3.52276
C  8.39566  1.22413  2.96155
H  9.82752 -0.01816  1.96080
C  6.62906  0.01263  4.14035
O  6.58965 -2.39273  4.03345
C  5.46601 -2.79204  3.39087
C  8.44465  3.03160  1.19980
H  9.78663  1.88886 -0.00653
C  6.88825  4.42739 -0.06144
O  6.94098  4.49375 -2.45356
C  5.68091  4.01607 -2.69620
C  9.00508  2.55880  2.54067
C  7.25898  1.19783  3.77779
H  5.73239  0.02309  4.74502
C  3.31637 -3.96852  2.42478
C  7.36048  3.91810  1.13884
H  6.05808  5.12168 -0.07638
C  3.38989  2.73177 -2.85501
H  8.67719  3.28946  3.28103
O  6.74100  2.40860  4.25577
C  3.29368 -2.66847  2.91298
O  6.72963  4.29972  2.32750
C  3.47242  4.03824 -2.39133
C  5.54460  2.75377  3.69646
C  5.55019  3.67493  2.61466
C  3.33811  2.52347  3.47787
C  3.35804  3.34015  2.35416
C 10.54114  2.54228  2.55417
H 10.92860  3.53496  2.31026
```

```
H 10.96729  1.83795  1.83407
C 10.47904 -2.59450  2.57366
H 10.85379 -2.26006  3.54479
H 10.90578 -1.93719  1.81095
C 10.50153 -2.71024 -2.49461
H 10.85502 -3.71476 -2.24771
H 10.92883 -2.02247 -1.75954
C 10.58755  2.41795 -2.56913
H 10.95053  2.06305 -3.53750
H 10.99381  1.75419 -1.80070
H 10.99532  3.41597 -2.38873
H 10.89721 -2.43326 -3.47551
H 10.85432 -3.60362  2.38441
H 10.90447  2.26059  3.54618
C -9.07761 -3.68066  0.04976
C -8.47606 -3.10635 -1.23293
C -8.44827 -3.02818  1.28533
H -8.78864 -4.73232  0.08000
C -8.95652 -1.91764 -1.80418
C -7.38682 -3.71883 -1.86795
C -8.96554 -1.88396  1.89893
C -7.28479 -3.56741  1.84967
C -8.36608 -1.31062 -2.91608
H -9.81646 -1.43922 -1.34947
C -6.74507 -3.13787 -2.95666
O -6.89881 -4.95390 -1.40769
C -8.38395 -1.28564  3.02710
H -9.85088 -1.42441  1.47502
C -6.68996 -3.04361  2.98747
O -6.74351 -4.70438  1.25021
C -8.90577 -0.04452 -3.58251
C -7.22637 -1.93275 -3.43879
H -5.88045 -3.61010 -3.40392
C -5.65633 -4.85267 -0.84644
C -8.94037  0.00163  3.63005
C -7.25087 -1.91044  3.56676
H -5.80298 -3.49702  3.41005
C -5.58941 -4.57880  0.54788
H -8.55816 -0.07797 -4.61602
C -8.31122  1.22558 -2.97892
O -6.59120 -1.31903 -4.51861
C -8.36522  1.24706  2.94860
H -8.58375  0.03197  4.66067
O -6.65799 -1.41031  4.73760
C -8.86473  1.81387 -1.83550
C -7.20804  1.87291 -3.55144
C -5.43073 -0.65643 -4.27997
C -3.43540 -4.67269 -0.97418
C -8.93933  1.84280  1.82097
C -7.22282  1.86690  3.46939
C -5.44755 -0.80719  4.54015
C -3.39344 -4.26724  0.35186
C -8.37038  2.98421 -1.25462
H -9.72669  1.33923 -1.38168
C -6.66278  3.02991 -3.00066
O -6.62857  1.36539 -4.72635
C -5.42780  0.74575 -4.52194
C -8.44151  3.01989  1.24625
H -9.80664  1.37294  1.37115
C -6.71656  3.05730  2.97145
O -6.59313  1.26491  4.55919
C -5.43774  0.59612  4.31126
C -9.02383  3.61987 -0.02853
C -7.23970  3.55767 -1.85256
H -5.79833  3.50001 -3.45251
C -3.25616 -0.55968 -3.80823
C -7.34585  3.62435  1.87237
H -5.84707  3.52179  3.41680
C -3.24012 -0.85061  4.21128
H -8.73384  4.67119 -0.03672
O -6.71948  4.73204 -1.30040
C -3.22217  0.77556 -4.18778
O -6.87455  4.85074  1.38164
C -3.26605  0.48471  3.82802
C -5.55607  4.66569 -0.59766
C -5.63592  4.80748  0.81788
C -3.33477  4.55833 -0.42968
C -3.40789  4.76288  0.94055
C -10.55733  3.56187 -0.07612
H -10.97690  4.08437  0.78763
H -10.94796  2.54081 -0.06402
C -10.44169 -0.01443 -3.61561
H -10.78794  0.87071 -4.15571
H -10.89076  0.01096 -2.61893
C -10.61156 -3.62082  0.06653
H -11.01622 -4.15584 -0.79658
H -10.99954 -2.59920  0.03115
C -10.47570  0.02179  3.67352
H -10.84696 -0.84853  4.22121
H -10.93211  0.00563  2.67991
H -10.82370  0.92631  4.17927
H -10.99619 -4.08751  0.97725
H -10.82179 -0.90422 -4.12450
H -10.92411  4.04376 -0.98630
C  1.92189  2.09937  3.73821
C  1.95823  3.42117  1.81179
C  1.91579 -2.08441 -3.74028
C  1.92434 -3.47630 -1.86995
C -1.88535 -0.95748 -3.34611
C -1.80603  1.26622 -4.06296
C -1.89619  4.35216 -0.79971
C -2.00635  4.77149  1.49559
C -2.02317 -4.68579 -1.50429
C -1.98211 -3.89788  0.69080
C -1.81784 -1.33453  4.13126
C  1.90273 -4.36522  2.08610
C  1.90191 -2.12679  2.75305
C -1.89159  0.87232  3.36110
C  2.08171  4.51900 -2.08402
C  1.96409  2.27199 -2.72319
N  1.26485  3.39635 -2.33031
N  1.18649  2.69362  2.69880
N -1.18764  4.51887  0.37457
N -1.09624  0.15799 -3.56345
N  1.17089 -2.68534 -2.71508
N  1.15213 -3.19087  2.30439
N -1.24480 -4.20954 -0.42892
N -1.10689 -0.23714  3.60240
O  1.57015  4.00949  0.81563
O  1.46440  1.42497  4.62993
O  1.53198 -0.97751  2.93976
O -1.33915 -2.39459  4.45107
O -1.54786  1.92675  2.84815
O -1.62054  4.94780  2.62446
O -1.45232  4.04027 -1.89268
O -1.33745  2.35198 -4.30696
O  1.69806  5.60914 -1.73943
O  1.54464 -4.04574 -0.86057
O  1.48524 -1.33189 -4.58255
O  1.52721  1.14376 -2.88787
O -1.60389 -5.03414 -2.57988
O -1.54370 -2.02407 -2.86494
O -1.58304 -3.40278  1.73412
O  1.46237 -5.43083  1.73436
H  0.17054  2.56789  2.64660
H -0.10222 -0.30875  3.40718
H  0.13735 -3.16244  2.13365
H -0.22308 -4.10511 -0.49642
H  0.16578 -2.49277 -2.60239
H -0.08984  0.21645 -3.40045
H  0.24467  3.47630 -2.25884
H -0.16560  4.41977  0.45148
N  4.44907 -4.66254  2.26719
N  4.36235 -2.06300  3.43766
N  4.45932  3.94997  1.91199
N  4.43548  2.19518  4.15725
N  4.63152  4.69034 -2.25849
N  4.42953  2.07239 -3.37615
N  4.40157 -4.08933 -2.00172
N  4.43209 -2.24490 -4.17525
N -4.35566 -1.31183 -3.86798
N -4.31639  1.46593 -4.51687
N -4.57922 -4.94487 -1.61097
N -4.46307 -4.23384  1.15034
N -4.40322  4.51344 -1.23037
N -4.56548  4.87820  1.59521
N -4.34074 -1.53450  4.53907
N -4.36012  1.24474  3.89664
C  0.31775  0.06617 -0.14256
O  0.26148 -1.13060 -0.30723
C -0.78617  1.07901 -0.28434
H -0.58280  1.60552 -1.22061
H -0.64166  1.81960  0.50748
C -2.20350  0.57447 -0.26024
C -2.60518 -0.48887  0.55567
C -3.18129  1.26530 -0.98225
C -3.95279 -0.81992  0.67526
H -1.86566 -1.06145  1.09875
C -4.52851  0.93121 -0.86162
H -2.89506  2.08046 -1.63622
C -4.93734 -0.10720 -0.02246
H -4.24358 -1.63807  1.32792
H -5.27050  1.49191 -1.41924
C -6.38791 -0.46670  0.11413
H -6.61379 -1.38925 -0.42607
H -6.65497 -0.63064  1.15843
H -7.03748  0.31656 -0.28199
C  5.01342 -1.44077 -0.02941
C  4.97993 -0.03721  0.57366
N  3.68335  0.64627  0.57736
C  2.65555  0.03319  0.19680
O  1.46791  0.73278  0.18929
H  5.27835 -0.09285  1.62805
H  2.55411 -0.99503 -0.14248
H  4.73410 -1.40061 -1.08735
H  4.34974 -2.15204  0.46861
H  6.02867 -1.83380  0.02954
C  5.97774  0.85502 -0.16420
H  5.92748  1.87951  0.20187
H  6.99600  0.48246 -0.03590
H  5.74899  0.86165 -1.23326
```

5b-TS@1_2

```
C  9.00025 -2.32871 -2.80315
C  8.38878 -2.89619 -1.52357
C  8.41486 -0.95129 -3.09877
H  8.67749 -2.98242 -3.61387
C  8.89061 -2.57213 -0.25932
C  7.28999 -3.76401 -1.56636
C  8.96982  0.23222 -2.59570
C  7.26117 -0.83596 -3.88370
C  8.36399 -3.08853  0.92818
H  9.73154 -1.89101 -0.19869
C  6.73053 -4.31049 -0.41750
O  6.75554 -4.11657 -2.81287
C  8.40772  1.48982 -2.83465
H  9.87795  0.16926 -2.00771
C  6.64459  0.38524 -4.12818
O  6.74587 -1.99553 -4.47268
C  8.94002 -2.72568  2.29353
C  7.27810 -3.96608  0.81235
H  5.88396 -4.98241 -0.47832
C  5.55319 -3.55424 -3.12132
C  9.01881  2.80118 -2.33890
C  7.22582  1.52141 -3.58479
H  5.74280  0.44528 -4.72235
C  5.54795 -2.43867 -4.00455
H  8.62805 -3.51687  2.97593
C  8.32420 -1.43496  2.82865
O  6.74995 -4.54592  1.97303
C  8.42048  3.19968 -0.99250
H  8.71370  3.56951 -3.05058
O  6.67589  2.77716 -3.83924
```

C	8.84867	-0.17200	2.54442
C	7.19822	-1.48121	3.66011
C	5.53309	-4.07800	2.37098
C	3.32998	-3.41944	-2.97374
C	8.89438	2.64037	0.20118
C	7.36956	4.12049	-0.88900
C	5.54434	3.15158	-3.19852
C	3.32532	-2.34240	-3.84978
C	8.31819	1.01054	3.07229
H	9.71477	-0.10513	1.89654
C	6.63185	-0.33965	4.21516
O	6.67751	-2.73732	3.97936
C	5.49990	-3.09603	3.40087
C	8.35377	2.93837	1.45303
H	9.72100	1.94132	0.14844
C	6.77128	4.42233	0.33160
O	6.87347	4.76522	-2.03686
C	5.62846	4.31458	-2.38026
C	8.91873	2.37435	2.75380
C	7.20862	0.89088	3.91794
H	5.77124	-0.40819	4.86829
C	3.30266	-4.01914	2.26445
C	7.25720	3.80809	1.47521
H	5.93538	5.10807	0.38025
C	3.35688	3.06127	-2.80044
H	8.58883	3.04682	3.54616
O	6.68143	2.04912	4.51788
C	3.27669	-3.03782	3.24551
O	6.66235	4.07806	2.71411
C	3.41010	4.28754	-2.15218
C	5.48671	2.45884	4.00989
C	5.48931	3.45085	2.98398
C	3.26701	2.29328	3.81634
C	3.29142	3.15640	2.72987
C	10.45515	2.36578	2.76738
H	10.83576	3.37664	2.59863
H	10.88692	1.71967	1.99812
C	10.47574	-2.69174	2.29815
H	10.84273	-2.48751	3.30757
H	10.89027	-1.92624	1.63628
C	10.53536	-2.34187	-2.78018
H	10.89736	-3.36122	-2.62248
H	10.95868	-1.71823	-1.98787
C	10.55395	2.77416	-2.31863
H	10.93838	2.52848	-3.31223
H	10.96057	2.04075	-1.61680
H	10.94056	3.75467	-2.02868
H	10.92806	-1.97666	-3.73302
H	10.87176	-3.65719	1.97234
H	10.81883	2.01355	3.73630
C	-9.00563	-3.57515	-0.27540
C	-8.40048	-2.88881	-1.49532
C	-8.39033	-3.02392	1.01035
H	-8.70744	-4.62266	-0.33230
C	-8.89089	-1.66630	-1.97311
C	-7.30631	-3.44675	-2.16913
C	-8.90638	-1.91555	1.68569
C	-7.25844	-3.63181	1.56792
C	-8.32250	-0.99056	-3.05583
H	-9.74832	-1.22626	-1.47702
C	-6.68636	-2.80024	-3.23242
O	-6.81964	-4.70394	-1.78134
C	-8.36808	-1.43012	2.88467
H	-9.76451	-1.40497	1.26462
C	-6.70082	-3.21194	2.76688
O	-6.72025	-4.73527	0.90497
C	-8.87700	0.31779	-3.61644
C	-7.19517	-1.57710	-3.64134
H	-5.82325	-3.23608	-3.71819
C	-5.59155	-4.67061	-1.18430
C	-8.94135	-0.19858	3.57872
C	-7.27229	-2.12224	3.41667
H	-5.83990	-3.71670	3.18581
C	-5.54809	-4.57864	0.23488
H	-8.53536	0.37416	-4.65062
C	-8.28069	1.52910	-2.90594
O	-6.59567	-0.89862	-4.70432
C	-8.35169	1.09121	3.00683
H	-8.60678	-0.25048	4.61559
O	-6.72625	-1.72674	4.64954
C	-8.83389	2.02563	-1.71990
C	-7.16525	2.20454	-3.41944
C	-5.41869	-0.27156	-4.45254
C	-3.36218	-4.57262	-1.24478
C	-8.91091	1.77907	1.92609
C	-7.20501	1.65155	3.58199
C	-5.50209	-1.13197	4.52783
C	-3.33202	-4.37392	0.12737
C	-8.33116	3.14314	-1.04873
H	-9.70155	1.52343	-1.30879
C	-6.60920	3.30621	-2.77678
O	-6.60212	1.79561	-4.63854
C	-5.40445	1.14949	-4.52656
C	-8.39612	2.99117	1.44790
H	-9.78100	1.35881	1.43508
C	-6.67759	2.86767	3.17768
O	-6.60353	0.96212	4.63499
C	-5.45946	0.28114	4.36917
C	-8.97353	3.69354	0.22381
C	-7.19587	3.75106	-1.59914
H	-5.74176	3.80629	-3.18828
C	-3.22584	-0.25435	-4.07473
C	-7.29302	3.52748	2.12234
H	-5.80329	3.28341	3.66025
C	-3.29223	-1.20550	4.24415
H	-8.66963	4.73845	0.29422
O	-6.67613	4.87863	-0.95517
C	-3.18582	1.11770	-4.28027
O	-6.79394	4.77887	1.72888
C	-3.28117	0.14538	3.92561
C	-5.50784	4.72925	-0.27752
C	-5.56404	4.74332	1.14544
C	-3.29387	4.52106	-0.16057
C	-3.33882	4.59555	1.22415
C	-10.50776	3.65866	0.17683
H	-10.91959	4.11357	1.08163
H	-10.91144	2.64502	0.10531
C	-10.41296	0.34096	-3.63738
H	-10.76783	1.26655	-4.09839
H	-10.85587	0.27856	-2.63968
C	-10.54045	-3.52973	-0.26356
H	-10.93507	-3.98866	-1.17396
H	-10.93805	-2.51268	-0.20897
C	-10.47733	-0.18611	3.58950
H	-10.85544	-1.09465	4.06564
H	-10.91163	-0.13463	2.58736
H	-10.84136	0.67873	4.15055
H	-10.92558	-4.08008	0.59900
H	-10.79116	-0.50479	-4.21780
H	-10.86961	4.21829	-0.68982
C	1.84357	1.85638	4.03745
C	1.91322	3.20580	2.13603
C	1.90493	-1.88272	-4.02711
C	1.91547	-3.66946	-2.52248
C	-1.84286	-0.73337	-3.75700
C	-1.74938	1.56351	-4.19123
C	-1.87324	4.31708	-0.58203
C	-1.92896	4.49616	1.74640
C	-1.93977	-4.58481	-1.74644
C	-1.90812	-4.16036	0.55022
C	-1.88453	-1.72243	4.16327
C	1.87792	-4.32332	1.87268
C	1.84986	-2.64928	3.50540
C	-1.88573	0.52310	3.51829
C	2.00652	4.67943	-1.77569
C	1.94637	2.55368	-2.74608
N	1.21931	3.58319	-2.17875
N	1.12485	2.46422	2.98906
N	-1.14211	4.31852	0.58915
N	-1.03741	0.38566	-3.89901
N	1.15495	-2.73205	-3.21682
N	1.10543	-3.47461	2.67505
N	-1.16111	-4.34886	-0.59686
N	-1.12956	-0.61184	3.73852
O	1.57952	3.73895	1.08770
O	1.37814	1.14035	4.88926
O	1.45303	-1.77958	4.25359
O	-1.46363	-2.83063	4.39108
O	-1.51598	1.59489	3.06654
O	-1.51135	4.55735	2.87652
O	-1.46178	4.14127	-1.71726
O	-1.27386	2.66569	-4.32693
O	1.59977	5.68948	-1.25512
O	1.52363	-4.48472	-1.71638
O	1.48101	-0.97279	-4.70806
O	1.54560	1.45082	-3.07429
O	-1.52348	-4.75951	-2.86583
O	-1.51055	-1.85343	-3.41762
O	-1.52251	-3.85334	1.66401
O	1.46284	-5.12074	1.06444
H	0.11946	2.26859	2.87907
H	-0.11558	-0.67248	3.63739
H	0.08738	-3.46818	2.59337
H	-0.13987	-4.32731	-0.64340
H	0.14026	-2.61923	-3.13420
H	-0.01923	0.36658	-3.85517
H	0.19429	3.62683	-2.11117
H	-0.12362	4.20144	0.64382
N	4.43534	-4.54866	1.79543
N	4.37224	-2.54860	3.82902
N	4.39732	3.76944	2.30421
N	4.37089	1.90133	4.45728
N	4.55931	4.91865	-1.88930
N	4.41865	2.47465	-3.35816
N	4.44394	-4.04791	-2.59095
N	4.43518	-1.82454	-4.37887
N	-4.33760	-0.98214	-4.17234
N	-4.28354	1.85458	-4.47246
N	-4.50006	-4.69682	-1.93445
N	-4.42040	-4.38432	0.90042
N	-4.37189	4.59328	-0.94305
N	-4.48100	4.69631	1.90785
N	-4.41229	-1.88247	4.51033
N	-4.36095	0.92563	4.00566
C	-0.17182	0.51164	-0.39902
H	0.07378	0.76284	-1.43541
H	0.15131	1.35238	0.21858
C	-1.66822	0.26378	-0.25941
C	-2.20627	-0.74715	0.54359
C	-2.55717	1.10795	-0.93580
C	-3.58890	-0.90274	0.66576
H	-1.55338	-1.42544	1.07672
C	-3.93785	0.95481	-0.80670
H	-2.17117	1.90170	-1.56370
C	-4.48157	-0.05671	-0.00550
H	-3.97633	-1.70080	1.29351
H	-4.59828	1.63243	-1.34165
C	-5.97364	-0.24973	0.10139
H	-6.33869	-0.93697	-0.67043
H	-6.25502	-0.67228	1.06892
H	-6.51353	0.69295	-0.02602
C	4.58104	1.30686	-0.02412
C	3.81682	0.07593	-0.52900
H	3.59298	0.21583	-1.59207
H	4.89764	1.17046	1.01550
H	3.96794	2.20934	-0.07608
H	5.48638	1.46357	-0.61809
C	4.65216	-1.20239	-0.36105
H	4.08153	-2.08843	-0.64547
H	5.55889	-1.15536	-0.97215
H	4.96296	-1.32404	0.68344
N	2.51494	-0.07120	0.12916
C	2.29036	-0.16638	1.39602
H	3.01992	-0.02035	2.19854
C	0.65835	-0.69236	-0.05622
O	0.56945	-1.82372	-0.39518
O	1.07333	-0.48133	1.69792

5b@1$_2$

```
C  8.86209  2.70712  2.54427
C  8.29659  3.14329  1.19316
C  8.26622  1.37599  2.98807
H  8.52682  3.44990  3.26884
C  8.84571  2.73919 -0.02586
C  7.19003  3.99871  1.13074
C  8.81123  0.15130  2.58133
C  7.14329  1.33042  3.82248
C  8.37009  3.18556 -1.26556
H  9.67932  2.04639 -0.01360
C  6.70219  4.50436 -0.06483
O  6.58850  4.37650  2.33820
C  8.27303 -1.08007  2.95932
H  9.69633  0.16183  1.95635
C  6.54437  0.13131  4.19718
O  6.61779  2.53535  4.32810
C  8.95732  2.67092 -2.57600
C  7.31225  4.10230 -1.24989
H  5.86225  5.18665 -0.07940
C  5.41850  3.77499  2.66771
C  8.89228 -2.42357  2.58099
C  7.11286 -1.05016  3.74195
H  5.66255  0.12211  4.82410
C  5.41961  2.89815  3.79459
H  8.64825  3.37366 -3.35057
C  8.33002  1.32467 -2.93749
O  6.83629  4.64014 -2.45858
C  8.30883 -2.94949  1.27460
H  8.59151 -3.12775  3.35749
O  6.58032 -2.28289  4.12104
C  8.88651  0.09903 -2.55704
C  7.14227  1.28362 -3.67915
C  5.58642  4.19260 -2.78153
C  3.22267  3.44541  2.45090
C  8.78650 -2.49999  0.04060
C  7.28367 -3.90249  1.24878
C  5.45183 -2.73505  3.52275
C  3.20159  2.70858  3.62657
C  8.31875 -1.12855 -2.91250
H  9.80108  0.10043 -1.97601
C  6.54193  0.09326 -4.07099
O  6.61091  2.50666 -4.09977
C  5.47829  2.98254 -3.52770
C  8.29713 -2.95474 -1.18588
H  9.58493 -1.76687  0.03482
C  6.74100 -4.36694  0.05649
O  6.78275 -4.42399  2.45915
C  5.54465 -3.95285  2.78309
C  8.90424 -2.47576 -2.50283
C  7.15101 -1.09382 -3.68192
H  5.63466  0.08990 -4.65985
C  3.36527  4.23969 -2.56449
C  7.24520 -3.87940 -1.14249
H  5.92733 -5.08098  0.06114
C  3.25408 -2.71782  3.17183
H  8.59108 -3.19216 -3.26272
O  6.64704 -2.32181 -4.11669
C  3.27970  2.98630 -3.15419
O  6.70205 -4.36447 -2.34450
C  3.32054 -3.98685  2.61512
C  5.46469 -2.75858 -3.61867
C  5.49035 -3.85702 -2.70725
C  3.24038 -2.73916 -3.51842
C  3.25782 -3.82454 -2.65485
C 10.43981 -2.47943 -2.47134
H 10.80569 -3.48290 -2.23876
H 10.85488 -1.79869 -1.72284
C 10.49214  2.63800 -2.56647
H 10.86741  2.30287 -3.53718
H 10.90244  1.96878 -1.80508
C 10.39823  2.70856  2.56032
H 10.77411  3.70173  2.30049
H 10.83468  1.99725  1.85370
C 10.42793 -2.38463  2.56488
H 10.80690 -2.05622  3.53664
H 10.83118 -1.70773  1.80655
H 10.82275 -3.38235  2.35613
H 10.76232  2.44823  3.55779
H 10.88465  3.63892 -2.36849
H 10.83635 -2.18153 -3.44598
C -9.04202  3.71234 -0.00895
C -8.45280  3.11275  1.26372
C -8.39351  3.07219 -1.23574
H -8.75192  4.76350 -0.02008
C -8.95212  1.93130  1.82733
C -7.35754  3.71035  1.89903
C -8.88520  1.90502 -1.82512
C -7.25747  3.64436 -1.82156
C -8.38475  1.32796  2.95283
H -9.81721  1.46553  1.36958
C -6.73637  3.13544  3.00053
O -6.87282  4.93795  1.42176
C -8.31419  1.32101 -2.96179
H -9.75909  1.43487 -1.38964
C -6.65034  3.11040 -2.95110
O -6.76173  4.82657 -1.26222
C -8.95435  0.07173  3.61209
C -7.24778  1.94344  3.48867
H -5.86951  3.59769  3.45335
C -5.64311  4.88004  0.83822
C -8.89981  0.06186 -3.59241
C -7.19157  1.95667 -3.50924
H -5.77985  3.58075 -3.39013
C -5.59035  4.75534 -0.57964
H -8.61307  0.09150  4.64786
C -8.37169 -1.19627  2.99594
O -6.63633  1.32990  4.58414
C -8.35440 -1.20731 -2.94063
H -8.54636  0.04791 -4.62406
O -6.60977  1.44888 -4.68129
C -8.92356 -1.77105  1.84406
C -7.26488 -1.84311  3.56081
C -5.48451  0.65237  4.35120
C -3.41410  4.77996  0.91841
C -8.92580 -1.79686 -1.81057
C -7.23908 -1.85035 -3.48902
C -5.42117  0.80314 -4.49676
C -3.37262  4.59800 -0.45646
C -8.41529 -2.92706  1.24852
H -9.79413 -1.30111  1.40176
C -6.69752 -2.97692  2.98669
O -6.70948 -1.35930  4.75849
C -5.49986 -0.75172  4.58475
C -8.45191 -2.99121 -1.25260
H -9.77317 -1.30854 -1.34307
C -6.75207 -3.05433 -3.00357
O -6.61865 -1.24952 -4.58628
C -5.44500 -0.61030 -4.34054
C -9.05255 -3.57501  0.02069
C -7.27352 -3.48984  1.83323
H -5.83050 -3.44776  3.43204
C -3.31119  0.52885  3.88318
C -7.37593 -3.61507 -1.89523
H -5.90027 -3.53724 -3.46394
C -3.21090  0.77590 -4.18345
H -8.76590 -4.62702  0.04477
O -6.75526 -4.64991  1.25588
C -3.29306 -0.80538  4.26779
O -6.90731 -4.85036 -1.42190
C -3.26236 -0.58094 -3.89610
C -5.59460 -4.57823  0.55502
C -5.66911 -4.79368 -0.85074
C -3.37597 -4.45012  0.39077
C -3.44050 -4.75863 -0.96001
C -10.58656 -3.51275  0.04266
H -10.99366 -4.04583 -0.82059
H -10.97440 -2.49105  0.01032
C -10.49049  0.06846  3.63432
H -10.85533 -0.81361  4.16721
H -10.93329  0.05610  2.63469
C -10.57569  3.65468 -0.04796
H -10.99143  4.17301  0.82017
H -10.96664  2.63372 -0.03978
C -10.43576  0.08011 -3.63313
H -10.78659  0.97314 -4.15718
H -10.89060  0.08073 -2.63874
H -10.80537 -0.80230 -4.16221
H -10.94633  4.14115 -0.95413
H -10.85895  0.96170  4.14574
H -10.96885 -3.98164  0.95330
C  1.81631 -2.31868 -3.72982
C  1.83743 -4.11632 -2.24530
C  1.78954  2.25540  3.87450
C  1.85038  3.41472  1.84776
C -1.93276  0.91176  3.43458
C -1.87654 -1.29811  4.18523
C -1.93735 -4.25198  0.76746
C -2.03258 -4.84310 -1.48895
C -1.99988  4.73842  1.43957
C -1.95241  4.37494 -0.86816
C -1.77584  1.22083 -4.09403
C  1.97579  4.67704 -2.18107
C  1.87092  2.48905 -2.99638
C -1.88500 -1.04508 -3.52309
C  1.92423 -4.40333  2.24065
C  1.84613 -2.21892  3.05268
N  1.12198 -3.29333  2.58524
N  1.06365 -3.22099 -2.97571
N -1.22242 -4.55040 -0.37403
N -1.14855 -0.18777  3.71822
N  1.06894  2.73059  2.75874
N  1.16526  3.57066 -2.49728
N -1.21678  4.48221  0.29588
N -1.07527  0.06201 -3.71898
O  1.44999 -4.90429 -1.40433
O  1.38982 -1.39097 -4.38296
O  1.46396  1.35309 -3.16292
O -1.29899  2.31242 -4.29631
O -1.56198 -2.14547 -3.11685
O -1.64072 -5.09847 -2.60148
O -1.51480 -3.87915  1.84943
O -1.41484 -2.38412  4.44167
O  1.52937 -5.42097  1.72632
O  1.50741  3.84809  0.75778
O  1.32830  1.61351  4.78656
O  1.45794 -1.07358  3.24105
O -1.57889  4.88895  2.56033
O -1.59074  1.95463  2.90033
O -1.54285  4.11090 -1.98594
O  1.59745  5.72189 -1.70989
H  0.05349 -3.09589 -2.87637
H -0.06133  0.05378 -3.63045
H  0.14477  3.61417 -2.38647
H -0.20169  4.34452  0.35323
H  0.05403  2.56568  2.68400
H -0.14532 -0.27870  3.53831
H  0.11273 -3.31921  2.39857
H -0.20275 -4.55681 -0.45495
N  4.53007  4.85434 -2.33703
N  4.32773  2.34697 -3.67946
N  4.38336 -4.40200 -2.22516
N  4.34230 -2.18336 -4.02178
N  4.47425 -4.61412  2.37363
N  4.31345 -2.07723  3.66823
N  4.32559  4.01208  1.95681
N  4.30613  2.39527  4.30662
N -4.40191  1.29408  3.93881
N -4.39719 -1.48454  4.58786
N -4.55681  4.91299  1.59693
N -4.45400  4.58802 -1.23710
N -4.44837 -4.36171  1.18161
N -4.59435 -4.91075 -1.61556
```

```
N -4.29744  1.50436 -4.45233
N -4.37647 -1.30732 -3.98419
C -1.11851  0.13470 -0.01947
C -1.82307 -0.84141  0.69562
C -1.85720  1.05710 -0.76411
C -3.21652 -0.88704  0.66438
H -1.28558 -1.57675  1.27484
C -3.25391  1.01402 -0.79594
H -1.33801  1.81693 -1.33526
C -3.96086  0.03624 -0.08598
H -3.73270 -1.65552  1.23441
H -3.79491  1.74693 -1.38846
C -5.46829 -0.05028 -0.15286
H -5.78738 -0.82205 -0.86483
H -5.90168 -0.31263  0.81792
H -5.91313  0.89531 -0.47655
C  4.14927  0.38640  1.38424
C  3.36929  0.29989  0.07005
H  2.64191  1.11055  0.04562
H  3.49717  0.25928  2.25016
H  4.63754  1.36153  1.43931
C  0.38895  0.27332  0.04663
H  0.72051  0.96454 -0.73445
H  0.68613  0.72233  1.00331
N  2.57830 -0.96305 -0.00917
C  3.26781 -2.16984  0.01360
H  2.60379 -3.03852 -0.02433
C  1.16603 -1.02811 -0.09909
O  0.61410 -2.10373 -0.25968
O  4.48240 -2.26288  0.07917
H  4.92630 -0.37446  1.42375
C  4.28177  0.44555 -1.14978
H  4.75784  1.42943 -1.11644
H  5.06738 -0.30946 -1.14173
H  3.71795  0.36501 -2.08076
```

2·7b@1$_2$

```
C -8.88822  2.15411  2.93634
C -8.30020  0.74822  3.08714
C -8.32390  2.88942  1.72089
H -8.55274  2.71476  3.81002
C -8.88603 -0.40314  2.54970
C -7.12319  0.56927  3.82447
C -8.85156  2.68991  0.44105
C -7.25692  3.79337  1.82618
C -8.35352 -1.68479  2.74499
H -9.79428 -0.30190  1.96696
C -6.56789 -0.67838  4.07366
O -6.53270  1.70923  4.36655
C -8.36417  3.31786 -0.71002
H -9.67073  1.98896  0.33066
C -6.72785  4.43862  0.71259
O -6.68760  4.07350  3.07866
C -8.96678 -2.94227  2.13998
C -7.20262 -1.79009  3.53457
H -5.66616 -0.78118  4.66195
C -5.40224  2.18410  3.78369
C -8.97251  3.02857 -2.08261
C -7.27481  4.18645 -0.54102
H -5.89468  5.12247  0.82076
C -5.45635  3.49588  3.24028
H -8.65002 -3.77240  2.77195
C -8.39320 -3.22017  0.75185
O -6.70723 -3.07113  3.81472
C -8.38355  1.74252 -2.66105
H -8.65173  3.83854 -2.73852
O -6.72278  4.84853 -1.64868
C -8.89276 -2.58459 -0.38683
C -7.34460 -4.13090  0.55671
C -5.51863 -3.38782  3.23212
C -3.23378  2.09007  3.28538
C -8.95871  0.47708 -2.48479
C -7.19535  1.80685 -3.39801
C -5.50675  4.38612 -2.06893
C -3.24975  3.42593  2.90622
C -8.43116 -2.84178 -1.68332
H -9.67455 -1.84448 -0.26016
C -6.88277 -4.45934 -0.71045
O -6.73926 -4.72330  1.67246
C -5.53877 -4.20123  2.06347
C -8.37830 -0.69019 -2.99550
H -9.89687  0.40047 -1.94762
C -6.56281  0.67914 -3.90638
O -6.68119  3.07657 -3.66531
C -5.49084  3.41934 -3.11180
C -9.00347 -2.08081 -2.87673
C -7.43020 -3.81376 -1.81676
H -6.07294 -5.16717 -0.83466
C -3.29702 -3.22858  3.07270
C -7.16299 -0.55089 -3.67705
H -5.63679  0.75831 -4.45944
C -3.27223  4.28863 -1.97630
H -8.69444 -2.63043 -3.76709
O -6.90835 -4.13485 -3.08312
C -3.32307 -3.94825  1.88506
O -6.61335 -1.70888 -4.21979
C -3.26840  3.31528 -2.96814
C -5.64404 -3.63048 -3.24650
C -5.51303 -2.27794 -3.67115
C -3.43142 -3.73167 -2.97615
C -3.33515 -2.36500 -3.20517
C -10.53895 -2.04861 -2.86305
H -10.91337 -1.53668 -3.75372
H -10.94773 -1.53469 -1.98843
C -10.50284 -2.92098  2.13859
H -10.89056 -3.87048  1.76035
H -10.91897 -2.12508  1.51469
C -10.42450  2.15253  2.95074
H -10.79120  1.67515  3.86338
H -10.85894  1.61804  2.10110
C -10.50748  3.03239 -2.05777
H -10.87327  3.99822 -1.69985
H -10.92770  2.26150 -1.40583
H -10.89913  2.86348 -3.06454
H -10.79944  3.17879  2.91911
H -10.87561 -2.77293  3.15571
H -10.93229 -3.06837 -2.85682
C  8.93394 -0.59874  3.57642
C  8.37908  0.76114  3.16057
C  8.37723 -1.73697  2.71623
H  8.56532 -0.77933  4.58709
C  8.98435  1.53500  2.15954
C  7.22718  1.28615  3.76224
C  8.95419 -2.13676  1.50636
C  7.25723 -2.46065  3.14286
C  8.47432  2.76315  1.73101
H  9.88558  1.15750  1.69152
C  6.66935  2.49607  3.36176
O  6.62142  0.59440  4.82272
C  8.48063 -3.22421  0.76071
H  9.80319 -1.58030  1.12632
C  6.77471 -3.57185  2.46890
O  6.61088 -2.04622  4.30990
C  9.12666  3.61214  0.63602
C  7.29154  3.20045  2.34204
H  5.76795  2.86997  3.82928
C  5.43052  0.00342  4.51168
C  9.06325 -3.59997 -0.59701
C  7.40440 -3.94495  1.29006
H  5.91854 -4.11848  2.84065
C  5.44640 -1.36725  4.13444
H  8.85192  4.64700  0.84576
C  8.52500  3.27360 -0.72691
O  6.77535  4.42660  1.92808
C  8.39022 -2.77396 -1.69107
H  8.78462 -4.63858 -0.77826
O  6.95004 -5.08572  0.61283
C  8.96617  2.16635 -1.46722
C  7.47187  4.02156 -1.27052
C  5.63954  4.47153  1.18881
C  3.23415  0.05897  4.12596
C  8.87054 -1.51957 -2.07540
C  7.24981 -3.24743 -2.35394
C  5.70479 -4.98020  0.07069
C  3.28071 -1.23581  3.62527
C  8.36928  1.75939 -2.66344
H  9.79751  1.58905 -1.07952
C  6.82592  3.63946 -2.44153
O  7.01370  5.18485 -0.62726
C  5.75425  5.03072 -0.11581
C  8.28652 -0.74953 -3.08750
H  9.74663 -1.12798 -1.57272
C  6.63494 -2.52960 -3.37292
O  6.76317 -4.51260 -2.01194
C  5.60658 -4.60886 -1.30180
C  8.87750  0.59291 -3.51014
C  7.26339  2.50050 -3.09574
H  5.98527  4.20653 -2.81881
C  3.43666  4.31919  0.89771
C  7.16122 -1.28966 -3.72448
H  5.75851 -2.91900 -3.87528
C  3.47665 -5.03516  0.20674
H  8.51036  0.77575 -4.52084
O  6.59604  2.07273 -4.24566
C  3.53063  5.00456 -0.30343
O  6.55185 -0.57859 -4.77010
C  3.38383 -4.62119 -1.11387
C  5.42577  1.40682 -4.04983
C  5.38150  0.03503 -4.42361
C  3.26549  1.31032 -3.50667
C  3.19513  0.00850 -3.98227
C 10.41268  0.55966 -3.57877
H 10.74230 -0.24783 -4.23794
H 10.88069  0.40177 -2.60310
C 10.65922  3.52618  0.64732
H 11.07606  4.20248 -0.10354
H 11.03311  2.52216  0.42729
C 10.46889 -0.62106  3.63620
H 10.83084  0.16708  4.30194
H 10.93729 -0.47008  2.65961
C 10.59547 -3.51625 -0.63913
H 11.02474 -4.17591  0.11966
H 10.97585 -2.50853 -0.45018
H 10.96325 -3.82858 -1.62021
H 10.81346 -1.58559  4.01827
H 11.04589  3.81436  1.62855
H 10.78881  1.50743 -3.97301
C -2.04367 -4.27064 -2.75290
C -1.90734 -1.94283 -2.98615
C -1.84086  3.82688  2.55776
C -1.84929  1.55221  3.07598
C  1.99616  3.98181  1.14465
C  2.13404  5.24191 -0.81796
C  1.90908  1.70404 -2.99623
C  1.77614 -0.46796 -3.84692
C  1.81409  0.54041  4.04958
C  1.91507 -1.59566  3.11023
C  2.08050 -5.18379  0.75710
C -1.86804 -2.87906  3.38853
C -1.91737 -4.01046  1.34564
C  1.93525 -4.41880 -1.44781
C -1.85787  2.84819 -3.17960
C -1.84956  4.48555 -1.51211
N -1.09914  3.60785 -2.29390
N -1.21959 -3.13102 -2.78824
N  1.09567  0.61683 -3.26984
N  1.29859  4.61796  0.13515
N -1.09013  2.65062  2.73162
N -1.13618 -3.40570  2.31256
N  1.11750 -0.51523  3.43115
N  1.24360 -4.78817 -0.30739
O -1.46653 -0.81075 -2.94409
O -1.67797 -5.41057 -2.60120
```

```
O -1.54321 -4.48508  0.28670
O  1.71152 -5.55354  1.84429
O  1.47271 -3.97094 -2.48213
O  1.29876 -1.53850 -4.14250
O  1.59653  2.75007 -2.45375
O  1.76592  5.83458 -1.80124
O -1.44657  1.99162 -3.93283
O -1.50058  0.38650  3.15577
O -1.40448  4.90176  2.22392
O -1.42497  5.22663 -0.65126
O  1.33008  1.58173  4.42382
O  1.56066  3.26921  2.03215
O  1.58748 -2.60907  2.51212
O -1.40733 -2.29788  4.34153
H -0.20079 -3.22827 -2.73967
H  0.22043 -4.75311 -0.21472
H -0.12223 -3.26097  2.25623
H  0.10750 -0.43268  3.26778
H -0.08174  2.65622  2.55565
H  0.27932  4.72179  0.08856
H -0.08682  3.46105 -2.21701
H  0.08642  0.60341 -3.13579
N -4.39712 -2.91917  3.75995
N -4.44193 -4.46333  1.36578
N -4.37179 -1.61248 -3.58697
N -4.59938 -4.38223 -2.94445
N -4.37479  2.86263 -3.55775
N -4.39550  4.83235 -1.49626
N -4.30413  1.44477  3.75078
N -4.37456  4.14295  2.83318
N  4.48550  4.05499  1.68321
N  4.70225  5.33972 -0.85599
N  4.31580  0.71793  4.54801
N  4.38368 -1.98472  3.63893
N  4.37896  2.04280 -3.54325
N  4.25813 -0.66649 -4.42222
N  4.64426 -5.21296  0.82985
N  4.44277 -4.40529 -1.89902
C -6.27235  0.55626  0.14041
C -5.07652 -0.13874 -0.49533
C  0.77655 -1.22882 -0.43875
H -5.18812 -0.12924 -1.58529
H  0.61154 -1.56428 -1.46652
H  0.68901 -2.12114  0.18627
C  2.16934 -0.66129 -0.32061
C  2.51885  0.36870  0.56100
C  3.18836 -1.26850 -1.06333
C  3.85097  0.74969  0.71744
H  1.75187  0.88947  1.11842
C  4.51808 -0.88670 -0.90475
H  2.94218 -2.03747 -1.78620
C  4.87369  0.12257 -0.00783
H  4.09489  1.54246  1.41848
H  5.28749 -1.37633 -1.49111
C  6.30698  0.54392  0.14824
H  6.46298  1.55416 -0.23988
H  6.60621  0.55230  1.19693
H  6.98287 -0.12480 -0.38901
H -6.42053  1.56631 -0.23974
H -6.15358  0.60015  1.22583
C -4.96764 -1.57913 -0.01812
H -4.88437 -1.59837  1.07220
H -5.85324 -2.13873 -0.30961
H -4.09031 -2.08007 -0.43498
H -7.18126 -0.00644 -0.08114
N -3.80722  0.52001 -0.16714
H -1.14825  1.41393 -0.10221
C -0.42894 -0.38325 -0.11374
O -0.29098  0.91591 -0.30704
O -1.47582 -0.91924  0.25174
H -2.97222 -0.07692 -0.05769
C -3.58855  1.81794 -0.05080
H -4.46822  2.47141 -0.09933
O -2.45470  2.32047  0.12101
```

7b·7b@1$_2$

```
C  8.96576 -1.92939 -3.05551
C  8.35339 -2.66976 -1.86572
C  8.39545 -0.52090 -3.18502
H  8.64673 -2.47091 -3.94683
C  8.87990 -2.59362 -0.57372
C  7.23031 -3.48841 -2.03800
C  8.92717  0.56623 -2.47673
C  7.30545 -0.26281 -4.02493
C  8.35483 -3.30911  0.51023
H  9.73255 -1.94795 -0.39968
C  6.70164 -4.25943 -1.01419
O  6.64188 -3.53194 -3.30850
C  8.39466  1.85614 -2.54998
H  9.79643  0.39893 -1.85191
C  6.70923  0.99207 -4.09847
O  6.78725 -1.29633 -4.82613
C  8.91800 -3.15151  1.91866
C  7.27323 -4.16074  0.24985
H  5.83863 -4.89025 -1.18100
C  5.50644 -2.81077 -3.49058
C  9.01279  3.06703 -1.84879
C  7.24827  2.01776 -3.33770
H  5.84499  1.15942 -4.72738
C  5.56018 -1.72222 -4.40813
H  8.59386 -4.02970  2.47799
C  8.30090 -1.94429  2.62538
O  6.72910 -4.92914  1.29104
C  8.42217  3.25212 -0.45608
H  8.71187  3.94032 -2.42911
O  6.67819  3.29037 -3.38849
C  8.83086 -0.65389  2.53843
C  7.16167 -2.10556  3.42340
C  5.50239 -4.49639  1.71442
C  3.33461 -2.39919 -3.18411
C  8.89846  2.51824  0.63902
C  7.36336  4.13654 -0.21849
C  5.55034  3.53032 -2.67644
C  3.35410 -1.44901 -4.19588
C  8.29511  0.43968  3.23171
H  9.70344 -0.49281  1.91611
C  6.60284 -1.06382  4.14941
O  6.60481 -3.38496  3.50764
C  5.45218 -3.60273  2.82052
C  8.33635  2.59848  1.91464
H  9.73773  1.85026  0.48435
C  6.75093  4.23231  1.02670
O  6.86480  4.94269 -1.26271
C  5.62539  4.54135 -1.67362
C  8.89159  1.83907  3.11869
C  7.18754  0.19437  4.05343
H  5.72788 -1.22206  4.76600
C  3.28809 -4.30889  1.48521
C  7.22451  3.43828  2.05955
H  5.90241  4.88664  1.17759
C  3.37086  3.33032 -2.26499
H  8.55331  2.38435  4.00061
O  6.64947  1.24095  4.82104
C  3.26256 -3.35012  2.48974
O  6.59626  3.48523  3.30465
C  3.40866  4.43615 -1.42923
C  5.44463  1.68825  4.35599
C  5.43974  2.77905  3.44527
C  3.23657  1.48545  4.10350
C  3.26478  2.43728  3.09257
C 10.42796  1.83050  3.14173
H 10.80944  2.85495  3.12750
H 10.86477  1.30461  2.28805
C 10.45370 -3.12665  1.94463
H 10.80982 -3.07431  2.97687
H 10.87678 -2.27316  1.40732
```

```
C 10.50161 -1.94958 -3.02785
H 10.86210 -2.98114 -2.99850
H 10.92026 -1.43040 -2.16122
C 10.54815  3.02270 -1.84309
H 10.92607  2.94237 -2.86583
H 10.94946  2.17822 -1.27587
H 10.94553  3.93739 -1.39550
H 10.89838 -1.46690 -3.92496
H 10.85101 -4.03531  1.48445
H 10.78541  1.33783  4.04983
C -8.95006 -3.54281 -0.93804
C -8.35433 -2.67159 -2.03803
C -8.36079 -3.18516  0.42825
H -8.62999 -4.56434 -1.14733
C -8.85527 -1.38913 -2.30282
C -7.26628 -3.10092 -2.80752
C -8.91278 -2.22464  1.28012
C -7.22236 -3.85501  0.89292
C -8.28978 -0.52787 -3.24527
H -9.71930 -1.04853 -1.74446
C -6.65024 -2.27171 -3.73854
O -6.74323 -4.39904 -2.63362
C -8.40610 -1.95670  2.55989
H -9.77772 -1.66714  0.94067
C -6.70614 -3.66570  2.16532
O -6.62971 -4.78531  0.03546
C -8.86752  0.84850 -3.57295
C -7.14987 -0.99113 -3.91494
H -5.78051 -2.60751 -4.28754
C -5.52115 -4.39033 -2.02472
C -9.00089 -0.87456  3.45493
C -7.31958 -2.72818  2.99046
H -5.84432 -4.22318  2.50733
C -5.48682 -4.43068 -0.59962
H -8.52277  1.08767 -4.57977
C -8.29952  1.93213 -2.66202
O -6.52465 -0.12723 -4.81453
C -8.40001  0.49701  3.14435
H -8.69260 -1.11735  4.47265
O -6.82251 -2.56218  4.29484
C -8.84585  2.19756 -1.39899
C -7.21023  2.71575 -3.06315
C -5.39472  0.50624 -4.39699
C -3.30795 -4.08830 -2.05170
C -8.92728  1.35377  2.17485
C -7.28548  0.96150  3.85462
C -5.59846 -1.95616  4.32036
C -3.31223 -3.95709 -0.67107
C -8.34072  3.17783 -0.54060
H -9.70627  1.62188 -1.07805
C -6.64851  3.67966 -2.23297
O -6.65776  2.53867 -4.34458
C -5.43679  1.92484 -4.31188
C -8.41020  2.63045  1.91960
H -9.77525  1.01477  1.59160
C -6.76714  2.23590  3.68253
O -6.69981  0.09748  4.78682
C -5.55524 -0.53644  4.42367
C -8.97273  3.51357  0.81086
C -7.21327  3.88154 -0.98236
H -5.78413  4.24761 -2.55082
C -3.23455  0.56628 -3.84951
C -7.33808  3.05566  2.71500
H -5.91092  2.57128  4.25254
C -3.38409 -1.98908  4.03561
H -8.66340  4.53284  1.04493
O -6.67110  4.84516 -0.12770
C -3.23513  1.95164 -3.94994
O -6.80286  4.33925  2.52632
C -3.37156 -0.60274  3.96550
C -5.51828  4.51498  0.51404
C -5.57428  4.34441  1.92493
C -3.32354  4.12695  0.57316
C -3.35777  4.07024  1.96076
C -10.50799  3.49675  0.76020
H -10.91803  3.79936  1.72747
```

```
H -10.91802  2.51106  0.52218
C -10.40364  0.84545 -3.59990
H -10.77324  1.82831 -3.90394
H -10.84819  0.61348 -2.62786
C -10.48592 -3.53212 -0.93557
H -10.86269 -3.85404 -1.90995
H -10.90698 -2.54394 -0.73032
C -10.53678 -0.86045  3.42943
H -10.92442 -1.84084  3.71890
H -10.94672 -0.62175  2.44399
H -10.91626 -0.11412  4.13230
H -10.86514 -4.21629 -0.17180
H -10.76575  0.10057 -4.31360
H -10.86493  4.19336 -0.00299
C  1.80902  1.05078  4.29684
C  1.89128  2.53700  2.50246
C  1.95620 -0.91611 -4.37318
C  1.96028 -2.41936 -2.58702
C -1.86151  0.12668 -3.44367
C -1.82221  2.43523 -3.76974
C -1.91448  3.88146  0.12296
C -1.95102  3.87005  2.45976
C -1.88702 -3.92628 -2.53065
C -1.93569 -3.57658 -0.22770
C -1.97548 -2.48451  3.84137
C  1.88073 -4.50315  0.98304
C  1.86610 -2.81916  2.60737
C -1.98847 -0.16376  3.57777
C  1.99784  4.73769 -0.99330
C  1.97193  2.79768 -2.27783
N  1.22331  3.70471 -1.57671
N  1.09642  1.74803  3.30136
N -1.18516  3.73444  1.28675
N -1.09156  1.26439 -3.48236
N  1.21466 -1.55103 -3.35717
N  1.12988 -3.54405  1.68686
N -1.15488 -3.64018 -1.35292
N -1.23235 -1.32050  3.56789
O  1.55056  3.18196  1.51914
O  1.32606  0.31192  5.11893
O  1.46394 -1.94196  3.34849
O -1.53923 -3.60868  3.89554
O -1.62770  0.96217  3.27987
O -1.52447  3.85089  3.58866
O -1.49311  3.83576 -1.01972
O -1.35843  3.54317 -3.87628
O  1.57131  5.64135 -0.32176
O  1.58060 -3.04250 -1.60809
O  1.52345 -0.12951 -5.17895
O  1.60246  1.73419 -2.77037
O -1.42360 -4.02211 -3.63802
O -1.49760 -1.00432 -3.14349
O -1.59588 -3.22821  0.89892
O  1.44317 -5.29842  0.18706
H  0.09046  1.59306  3.17692
H -0.20807 -1.37039  3.45231
H  0.12314 -3.42891  1.54015
H -0.14395 -3.44254 -1.36851
H  0.20212 -1.38705 -3.22926
H -0.09831  1.31286 -3.23353
H  0.19922  3.65822 -1.43492
H -0.16667  3.58262  1.31051
N  4.41672 -4.88496  1.06235
N  4.33712 -2.99532  3.19462
N  4.35791  3.12715  2.76322
N  4.33577  1.06743  4.73195
N  4.55023  5.03847 -1.08237
N  4.43339  2.86482 -2.92768
N  4.40008 -3.12082 -2.83123
N  4.47965 -1.06683 -4.80458
N -4.30401 -0.18845 -4.10764
N -4.34731  2.66057 -4.14820
N -4.42617 -4.27084 -2.76063
N -4.39254 -4.14726  0.08977
N -4.39767  4.37352 -0.17725
N -4.49040  4.15595  2.66274
N -4.50947 -2.69508  4.17524
N -4.44903  0.15319  4.18744
C -5.36320 -0.76980 -0.84902
C -4.80844 -0.47412  0.54530
H -5.25986 -1.16889  1.26208
H -5.07834 -1.76286 -1.19471
H -4.97154 -0.04413 -1.56112
C -5.14423  0.95964  0.95654
H -4.77661  1.65293  0.19995
H -6.22837  1.06618  1.03628
H -4.69637  1.21373  1.91776
H -6.45416 -0.70383 -0.84715
N -3.36144 -0.74271  0.62005
H -3.05246 -1.59600  1.06842
C -2.41317 -0.03661 -0.03518
H -1.40132 -0.45245  0.13055
O -2.60936  0.95423 -0.73545
C  5.11856 -1.37205  0.02780
C  4.81974 -0.05175 -0.68360
H  5.28514 -0.06338 -1.67506
H  4.67635 -2.21908 -0.49783
H  4.72143 -1.33725  1.04228
C  5.38664  1.12122  0.11756
H  4.95934  1.11665  1.11972
H  6.47288  1.03567  0.20042
H  5.15018  2.07943 -0.34471
H  6.19996 -1.51851  0.07749
N  3.37840  0.11519 -0.94213
H  3.08636  0.43593 -1.85739
C  2.42813  0.13505  0.02101
H  1.42521  0.35208 -0.39247
O  2.61452 -0.06456  1.21917
```
